# Two-Photon-Driven Photoprotection Mechanism in Echinenone-Functionalized Orange Carotenoid Protein

**DOI:** 10.1021/jacs.4c13341

**Published:** 2025-01-21

**Authors:** Stanisław Niziński, Elisabeth Hartmann, Robert L. Shoeman, Mirosław Tarnawski, Adjélé Wilson, Jochen Reinstein, Diana Kirilovsky, Michel Sliwa, Gotard Burdziński, Ilme Schlichting

**Affiliations:** 1 Max Planck Institute for Medical Research, Jahnstr. 29, Heidelberg 69120, Germany; 2 CEA, CNRS, Institute for Integrative Biology of the Cell (I2BC), Université Paris-Saclay, Gif-sur-Yvette 91198, France; 3 CNRS UMR 8516 LASIRE Laboratoire de Spectroscopie pour les Interactions, la Réactivité et l’Environnement, Univ. Lille, Lille 59 000, France; 4 LOB, CNRS, INSERM, École Polytechnique, Institut Polytechnique de Paris, Palaiseau 91120, France; 5 Quantum Electronics Laboratory, Faculty of Physics and Astronomy, Adam Mickiewicz University, Poznań, Uniwersytetu Poznańskiego 2, Poznań 61-614, Poland

## Abstract

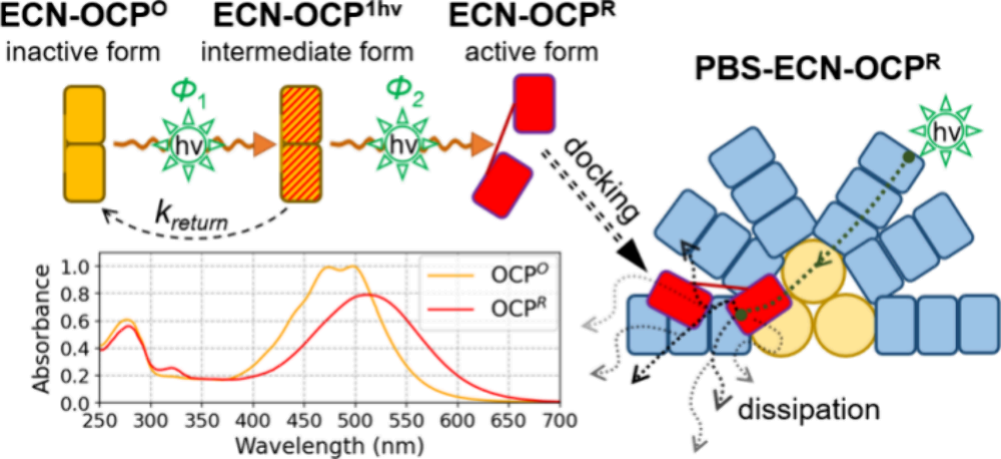

Orange carotenoid protein (OCP) is a photoactive protein that mediates photoprotection in cyanobacteria. OCP binds different ketocarotenoid chromophores such as echinenone (ECN), 3′- hydroxyechinenone (hECN), and canthaxanthin (CAN). In the dark, OCP is in an inactive orange form known as OCP^O^; upon illumination, a red active state is formed, referred to as OCP^R^, that can interact with the phycobilisome. Large gaps still exist in the mechanistic understanding of the events between photon absorption and formation of the OCP^R^ state. Recent studies suggested that more than one photon may be absorbed during the photocycle. Using a two-pulse excitation setup with variable time delays between the pulses, we demonstrate that canthaxanthin-functionalized OCP^O^ forms the OCP^R^ signature after absorption of a single photon. By contrast, OCP^O^ complexed with hECN or ECN does not photoconvert to OCP^R^ upon single photon absorption. Instead, OCP^R^ is formed only upon absorption of a second photon arriving roughly one second after the first one, implying the existence of a metastable light-sensitive OCP^1hv^ intermediate. To the best of our knowledge, a sequential 2-photon absorption mechanism in a single biological photoreceptor chromophore is unique. It results in a nonlinear response function with respect to light intensity, effectively generating a threshold switch. In the case of OCP, this prevents down regulation of photosynthesis at low light irradiance.

## Introduction

**Figure 1:**
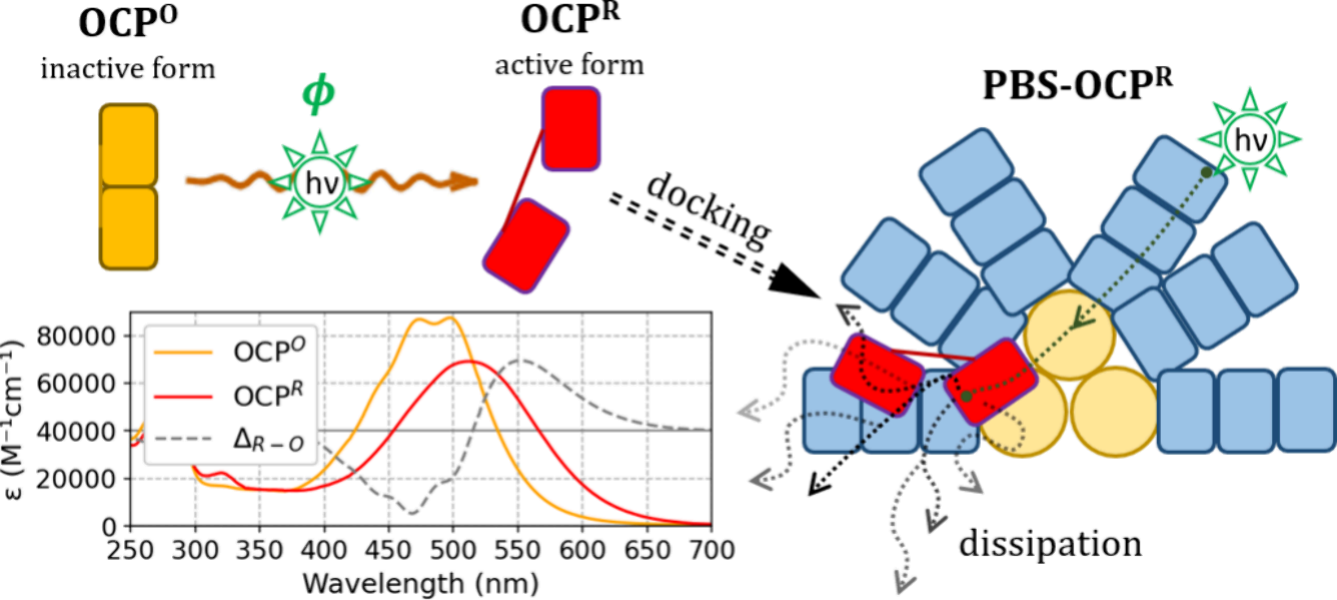
**Properties of OCP.** Upon illumination with blue-green light, the dark orange form of OCP (OCP^O^), which can be monomeric (shown) or dimeric, transforms into the biologically active monomeric red form (OCP^R^) that can bind to the phycobilisome (PBS, the antenna complex of cyanobacteria), inducing nonphotochemical quenching (NPQ). Presented here are UV–vis stationary spectra of echinenone-functionalized OCP^O^ from *Synechocystis* (recorded in the dark, with the sample kept at 11 °C) and after full photoconversion to OCP^R^ (452 nm irradiation of 3.2 mW/cm^2^ until full red form saturation was reached). The absorption is scaled to the molar absorption coefficient reported by Maksimov et al. 2020.^13^

Sunlight is essential for photosynthesis, but high light intensity can result in photodamage. In cyanobacteria, Orange Carotenoid Protein (OCP) plays a crucial role in a photoprotective process referred to as nonphotochemical quenching (NPQ).^1−8^ Under strong illumination conditions, OCP interacts with the antenna complex, the phycobilisome, thereby reducing the energy transfer to the photosystems and thus minimizing the accumulation of generated reactive singlet oxygen species. While this process protects the photosynthetic apparatus, it also reduces photosynthesis yield. Thus, for cyanobacteria to thrive in changing light conditions, the interaction between OCP and the phycobilisome must be tightly regulated as a function of light intensity. The regulation is rooted in OCP’s intrinsic light-sensitivity. In the dark or at low light intensity, the protein exists as an orange form, OCP^O^, with low affinity to the phycobilisome. Under strong illumination conditions, OCP^O^ switches to its metastable active red form, OCP^R^, that binds to the phycobilisome, initiating NPQ (Figure 1). The lifetime of OCP^R^ is usually significantly longer than 1 min.^3^ In order to fulfill its biological function, the yield of the photoproduct OCP^R^ must depend strongly on light intensity.

The photocycle of OCP is initiated by absorption of a green-blue photon, resulting in formation of a number of electronic ground-state intermediates (P_1_, P_2_, P_3_, P_N_, P_M_, P_X_ ···) on the ps to μs time-scale.^3,9−28^ Most studies performed with visible and infrared transient absorption spectroscopy focused on the early events of the reaction. However, it was not verified whether the observed dynamics do indeed result in formation of OCP^R^. Proving this is not trivial, because most time-resolved spectroscopy techniques involve high-repetition rate lasers (usually 1 kHz) – restricting the temporal observation window–while OCP^R^ formation occurs on a much longer time scale. Moreover, the quantum yield of the final OCP^R^ product is extremely low,^3^ which renders even normally routine experiments challenging.

Recently, we observed a surprising property of the OCP^O^ → OCP^R^ photoconversion process when measuring the speed of accumulation of OCP^R^-like absorbing species upon continuous irradiation.^29^ Starting from the dark-adapted state, and analyzing the first time-points of the reaction after switching on the irradiation light, one can calculate a so-called differential quantum yield ϕ_*d*_^30^ (see section 1.3 in Supporting Information) representing the effective probability of OCP^R^ formation after photon absorption by OCP^O^. This simplistic approach allows minimizing the number of *a priori* assumptions concerning the photoconversion mechanism. The dark-adapted sample was irradiated by a 452 nm LED source, while being probed continuously by weak 550 nm light. Within the first seconds after switching on the irradiation light, the absorbance grows linearly, because OCP^R^ relaxation processes occur on time scales that are much longer. This experiment was repeated separately for various irradiation light intensities. We found that ϕ_*d*_ increases with the irradiation intensity for echinenone-binding OCP, resulting in a nonlinear dependence.^29^ This is very unexpected, as the quantum yield is a measure of the events per photon absorbed and thus “normalized” with respect to the irradiation intensity. We concluded that we were not observing a reaction that yields the final photoproduct OCP^R^ after absorption of only one photon, but a sequential process, where one of the reaction intermediates absorbs another photon in order to form OCP^R^. In such a case it is much more appropriate to define two (or more) quantum yields describing two (or more) subsequent light-dependent steps. Interestingly, similar conclusions were drawn in a recent report by Rose et al. which showed that OCP photoactivation involves two light-driven reactions mediated by a metastable reaction intermediate.^31^ Moreover, Tsoraev et al.^32^ reported that repeated photoexcitation within at least 30 s results in a significant increase of the quantum yield of the photoproduct OCP^R^.

OCP can bind different carotenoid chromophores; 3′-hydroxyechinone (hECN) is the natural chromophore of OCP in *Synechocystis*.^1,33^ Due to difficulties of generating enough hECN when overexpressing OCP (even in native strains), the community has studied predominantly OCPs complexed with echinenone (ECN) and canthaxanthin (CAN), respectively, since the proteins involved in their biosynthesis can be overexpressed in *E. coli*. All three chromophores are photoactive and afford OCP^R^ formation.^2,3,34,35^ The photoconversion efficiency of CAN-functionalized OCPs is higher than that of their ECN counterparts,^10^ implying a significant influence of the additional carbonyl group in the β2 ionone ring in CAN compared to ECN.^17,36^

Here we investigate the photoconversion mechanism of OCP on the millisecond to minute time scale, addressing specifically the number of light-sensitive steps occurring along the reaction coordinate. We analyze the time-evolution of photoexcited OCP from *Synechocystis* (Syn) and *Planktothrix* (Plk) cyanobacteria complexed with different carotenoid chromophores (hECN, ECN, CAN). To this end, we use an experimental setup involving one or two submillisecond laser pulses with a variable time delay between pulses. We show that for ECN- and hECN-complexed OCPs the spectral signature associated with OCP^R^ formation is only observed when two excitation pulses separated by a time-delay of about 1 s are employed. Therefore, OCP functionalized with ECN or hECN exhibits a two-photon mechanism  involving a light-sensitive intermediate OCP^1hν^, resulting in a strictly nonlinear irradiation intensity dependent response. By contrast, for CAN complexed OCP, the spectral signature associated with OCP^R^ is observed already when using only a single light pulse.

## Results

### Setup for the Detection of a Sequential Two-Photon Photoconversion Mechanism

**Figure 2:**
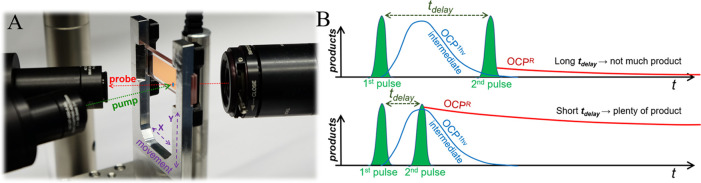
**Dedicated visible broadband transient absorption spectroscopy setup for pump–pump–probe experiments on OCP**. A) Geometry of the experiment. The sample is contained in a flat cell with 100 μm optical path length. A translation stage displaces the sample each time after the kinetics has been recorded. B) Experimental concept. In a two-photon mechanism, the first irradiation pulse populates an intermediate photoproduct OCP^1hv^, while the second pulse can photoconvert it to OCP^R^. A stable and temporally continuous broadband probe light (470–850 nm) is used to detect nascent products. The OCP^R^ yield is low if the lifetime of the one-photon generated intermediate OCP^1hv^ is much shorter than the time delay ***t*_*delay*_** between the two light pulses (shown in green, upper trace). By contrast, if the peak of the OCP^1hv^ intermediate concentration coincides with the second light pulse, the OCP^R^ yield is high (lower trace). Thus, fine-tuning of ***t*_*delay*_** is necessary to observe significant formation of OCP^R^.

The most straightforward approach to investigate the number of photon absorption events required to drive the OCP^O^ → OCP^R^ photoconversion is to utilize multiple excitation pulses. Such pump–pump–probe techniques are used to characterize nonlinear photochromic materials^37^ and have demonstrated, for example, the increase of photoproduct formation in experiments applying two femtosecond excitation pulses delayed by few picoseconds.^38^ Here we investigated the dynamics of OCP^R^ formation on the millisecond-to-second time-scale using two submillisecond pump pulses (200 μs duration, FWHM) and a temporally continuous visible light probe. If the OCP^O^ → OCP^R^ photoconversion process is single-photon driven, one excitation pulse should be sufficient to obtain a proper OCP^R^ signature, otherwise another pulse is required. However, observing OCP^R^ is far from trivial, not only because of its low quantum yield (between 0.1% and 1%,^3,16,25,27,29^ note the reported values are inconsistent and were determined using different methods), but also due to the fact that most time-resolved experiments operate at high repetition rates and do not allow to check whether or not the process initiated by the excitation laser pulse terminates in the expected way on a long time scale (>100 ms). Moreover, it is challenging to ensure that for each repetition of the experiment a fresh aliquot of the sample, free of any unrelaxed intermediates, is probed. This is complicated even more by the requirement that acquisition of seconds-long kinetics requires a nonexchanging (still) sample without diffusion processes mixing an irradiated sample aliquot in the beam focus and the rest of the sample. In order to overcome these challenges, we developed a dedicated visible broadband transient absorption spectroscopy setup for pump–pump–probe experiments probing in the millisecond–second time regime. The OCP solution was mounted in a quartz cuvette with a 100 μm light path, which greatly limits diffusive sample exchange due to capillary forces (see Figure 2A). The laser irradiation pulses were focused to a small ≈190 μm spot (FWHM), so that one cuvette could be probed at multiple places. Each kinetics was measured continuously for 20 s with ≈3 ms temporal resolution and then the cuvette was translated. This way, after each measurement, a fresh and completely dark-adapted sample aliquot is probed, with no memory effects of previous pulses.

We recorded transient absorption kinetics after a single irradiation pulse (denoted as 1*h*ν) and after a pair of pulses that are temporarily separated by *t*_*delay*_ (denoted as 2*h*ν), respectively. In both cases, the total absorbance difference Δ*A* is determined with respect to the absorbance recorded without irradiation pulses, i.e. Δ*A* represents the cumulative change in absorption caused by a single or two excitation pulses, respectively. As illustrated in Figure 2B, a variable *t*_*delay*_ allows to probe the formation and decay time of a OCP^1hv^ intermediate, should it exist. If the OCP^O^ → OCP^R^ photoconversion is a purely single-photon initialized process, then the 2*h*ν kinetics should be *t*_*delay*_-independent and simply resemble the rescaled 1*h*ν-kinetics.

### Kinetics Obtained for ECN and hECN OCPs after a Single Pump Pulse and Two Pump Pulses

**Figure 3:**
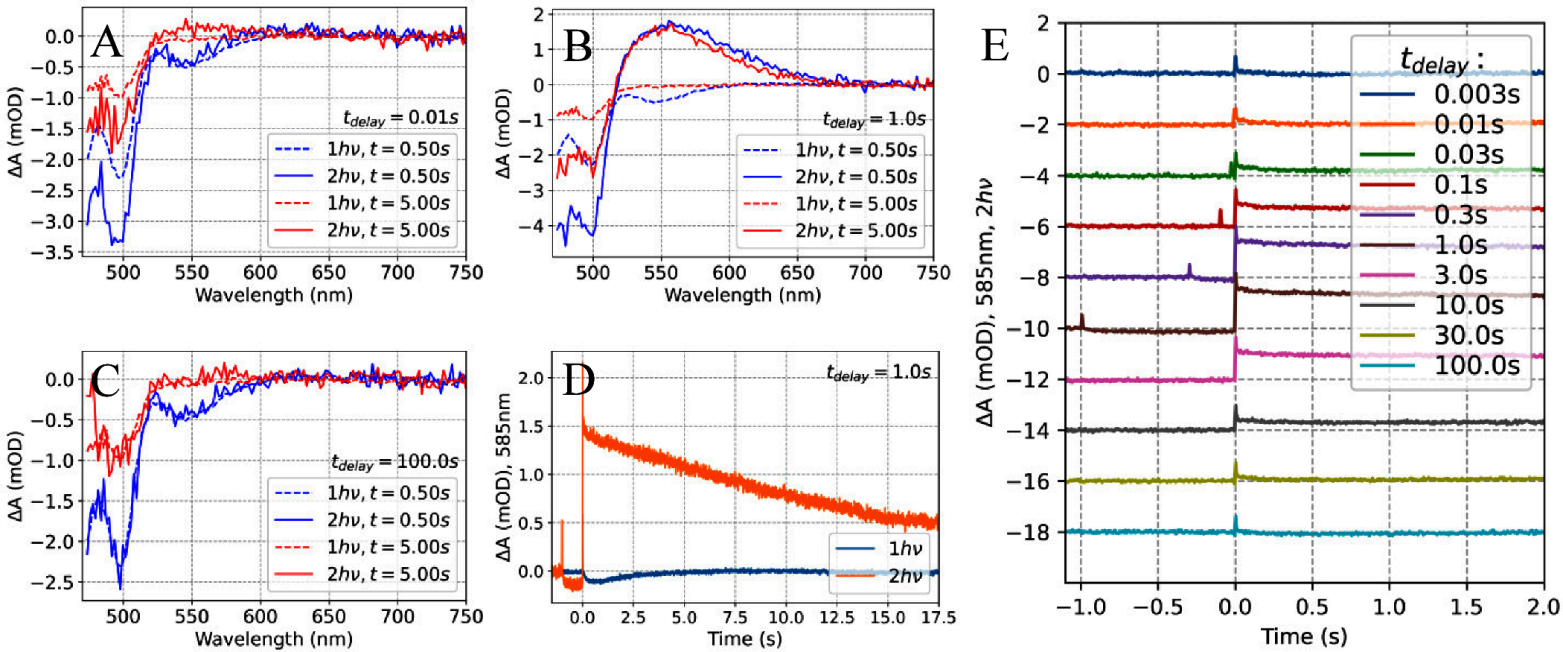
**Two-pulse excitation experiment analyzing ECN-functionalized OCP from*****Synechocystis*****without His-tag.** Transient absorption spectra obtained after two 512 nm excitation pulses (50 mJ/cm^2^) separated by *t*_*delay*_ of A) 10 ms, B) 1 s, C) 100 s, compared to the ΔA spectrum obtained after only one pulse. D) Kinetics at 585 nm measured after excitation with a single pulse (1*h*ν) and a pair of pulses (2*h*ν) separated by 1s. E) Kinetics at 585 nm recorded after two pulses separated by various delays. Time zero is set at the 2^nd^ pulse, and ΔA is set to zero before the 1^st^ excitation pulse.

We used this experimental setup (Figure 2) to investigate hECN and ECN-functionalized OCPs from *Synechocystis* (and *Planktothrix,* see Supporting Information and below) cyanobacteria. The 6-His affinity tags used for purification were removed proteolytically, to avoid effects of tagging.^10,12^ The experimental results are shown in Figure 3, demonstrating how the ECN-functionalized OCP reacts to single-pulse and two-pulse excitations. OCP^R^ is characterized by a red-shifted spectrum compared to OCP^O^ (Figure 1). The kinetics probed at 585 nm (Figure 3D) are especially well suited to track the presence of the OCP^R^ product because they take into account mostly absorption of the OCP^R^ form and minimize contributions from the bleached OCP^O^ population and intermediate forms populated after the first pulse, respectively. For ECN-OCP no measurable positive transient absorption signal at 585 nm is observed after one laser pulse (1*h*ν labeled kinetics). The only spectral feature observed is a pair of negative bands at ≈495 nm and ≈545 nm, of which only the first one lives longer than a few seconds (Figure 3A-C, dashed spectra). A recent report by Chukhutsina et al. describes a similar peculiar spectral signature with a dominating negative ΔA contribution occurring on the μs-ms time scale.^17^ This feature may indicate a *trans*-*cis* isomerization of the conjugated double bond system of the carotenoid, which is usually associated with a hypochromic effect and a change in vibrational structure.^39,40^ Importantly, our data clearly show that even after a strong short laser pulse (about 50 mJ/cm^2^) the protein cannot photoconvert to a species resembling the OCP^R^ protein form, for which a large band at 550 nm is expected. Such an intense laser pulse was used intentionally, because it demonstrates that even multiround excitation of an OCP molecule does not result in OCP^R^ formation if the re-excitation occurs within a short period of time (below single milliseconds), see also reference.^11^ An OCP^R^-like spectral signature appears only after a properly delayed second pulse (Figure 3B, continuous line), with maximal yield for a *t*_*delay*_ of about 1 s. For a 10 ms or 100 s delay, the positive transient absorption band is negligible (Figures 3A and 3C, respectively). *t*_*delay*_ must be roughly between 10 ms and 10 s in order to re-excite OCP^1hν^ and yield OCP^R^. This peculiar timing dependence is apparent in Figure 3E, showing that the OCP^R^ yield depends strongly on *t*_*delay*_, peaking at *t*_*delay*_ = 1s. Experiments performed on hECN-functionalized OCP show very similar kinetics as for ECN-functionalized protein (see Figure S9 in the Supporting Information).

**Figure 4:**
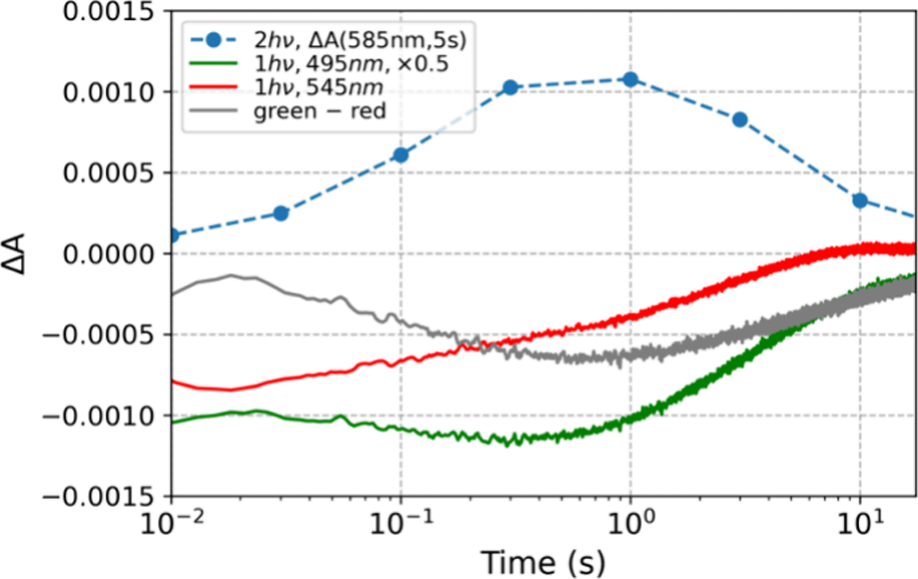
**Temporal evolution of spectroscopic signatures.** The green and red curves show the transient absorption kinetics probed at 495 and 545 nm, respectively, obtained after pumping with only one pulse, plotted as a function of time (scaled by a factor of 0.5 and 1, respectively). Only the 495 nm signal lives long enough to be considered as OCP^1hν^. This suggests that the OCP^1hν^ intermediate is associated exclusively with the negative ΔA peak at 495 nm, while an earlier intermediate form OCP^X^ is associated with both negative ΔA peaks at 495 and 545 nm, respectively. If so, one should be able to obtain the OCP^1hν^ concentration profile by subtracting the 545 nm kinetics from the 495 nm kinetics (with proper weights). Indeed, the difference between the green (495 nm) and red (545 nm) curves plotted as the gray curve, mirrors the temporal dependence of the blue curve, which represents the OCP^R^ product yield (ΔA observed at 585 nm, 5s after the second of two excitation pulses) plotted as a function of *t*_*delay*_. The data show ECN-functionalized OCP from *Synechocystis* (50 mJ/cm^2^ pump).

We conclude that the ≈495 nm negative ΔA feature represents a reaction intermediate (which we refer to as OCP^1hν^), which turns into OCP^R^ after re-excitation. Of note, the *t*_*delay*_ value resulting in the rise of this positive OCP^R^-like spectral signature (maximum at 550 nm) must be shorter than the decay of the negative ≈495 nm feature visible after only one excitation pulse (Figure 4, see also decomposition in Supporting Information, section 3). However, if *t*_*delay*_ value is very short (<10 ms), no OCP^R^ signature is observed either. This suggests that the OCP^1hν^ intermediate is formed after the decay of an earlier intermediate form dubbed OCP^X^ that is characterized by two negative transient absorption bands at ≈495 nm and ≈545 nm: . Note that OCP^1hν^ is characterized solely by the ≈495 nm negative ΔA band (Figure 4).

### Kinetics Obtained for CAN-OCPs after Single and Double Pump Pulses

**Figure 5:**
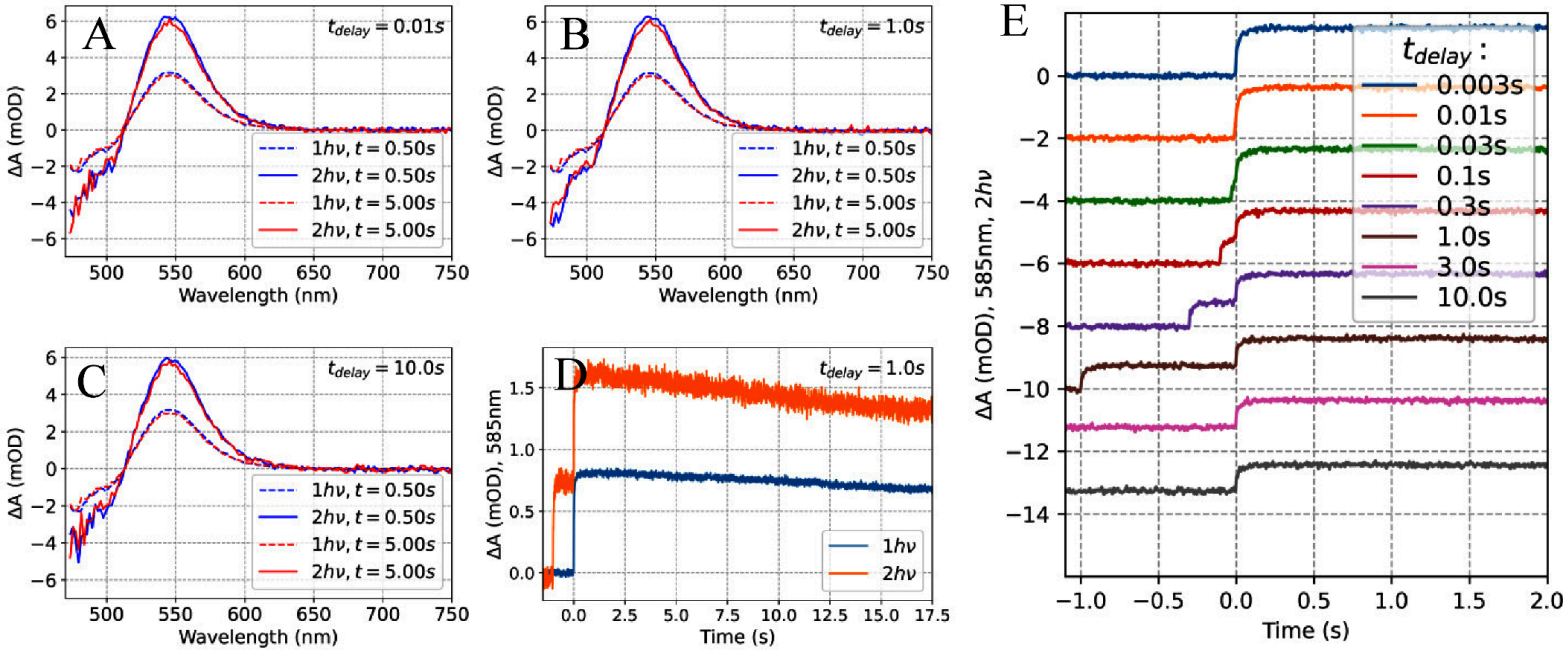
**Two-pulse excitation experiment analyzing CAN-functionalized OCP from *Synechocystis*without His-tag.** Transient absorption spectra obtained after two 512 nm excitation pulses (3 mJ/cm^2^) spaced by *t*_*delay*_ of A) 10 ms, B) 1 s, C) 10 s, compared to the ΔA spectrum obtained after only one pulse. D) Kinetics at 585 nm measured after excitation with a single pulse (1*h*ν) and a pair of pulses (2*h*ν) separated by 1s. E) Kinetics at 585 nm recorded after two pulses separated by various delays. Time zero is set at the 2^nd^ pulse, and ΔA is set to zero before the 1^st^ excitation pulse.

CAN-functionalized OCP reacts very differently than ECN-complexed OCP (see Figure 5). Already after one excitation pulse, a long-living transient absorption signal ΔA appears (see Figure 5D) that resembles the OCP^R^ spectral signature (Figure 5A-C, dashed spectra). In order to obtain signals of comparable magnitude as for ECN-OCP, a much weaker pump pulse of 3 mJ/cm^2^ (Figure 5) is sufficient (see Supporting Information for the results of a laser energy titration). The growth kinetics of the 585 nm absorbance resembles a Heaviside (step) function (Figure 5D). After a second pulse, another ΔA step is observed that has the same height as the first one and decays equally fast (Figure 5D and Figure S19A,D in the Supporting Information). The spectra obtained after two pulses with *t*_*delay*_ values of 10 ms, 1 and 10 s do not show significant variability (Figures 5A-C). In contrast to ECN-OCP, the 585 nm transient absorption signal is invariant of *t*_*delay*_ for CAN-OCP (Figure 5E). In conclusion, in CAN-OCP the OCP^R^ form is populated after only one laser pulse; use of two pulses leads to an additive effect (see the comparison in the Supporting Information, Figure S19A, D). These results explain not only the higher photoconversion efficiency of CAN-functionalized OCPs compared to their ECN counterparts observed previously,^10^ but also the observation that upon photoexcitation with a ns laser pulse CAN-OCP^O^ converts to OCP^R^ whereas ECN-OCP^O^ does not.^10^ We note that for higher pump pulse energies (see Figures S8 and S17 in Supporting Information) the second pulse leads to a faster decay and slightly broader spectral signature with an additional 550–630 nm flank (see also the direct comparison in Figure S19 in the Supporting Information), similar to observations by Rose et al.^31^ and by Wilson et al.^10^ (Figure 2B in reference (10)) upon a longer period of continuous irradiation. Since these effects are absent after weaker irradiation pulses (3 mJ/cm^2^), they must have a different origin than a two-photon photoconversion process.

### Chromophore Type Determines 1*h*ν or 2*h*ν-Driven Photoconversion Mechanism

**Figure 6:**
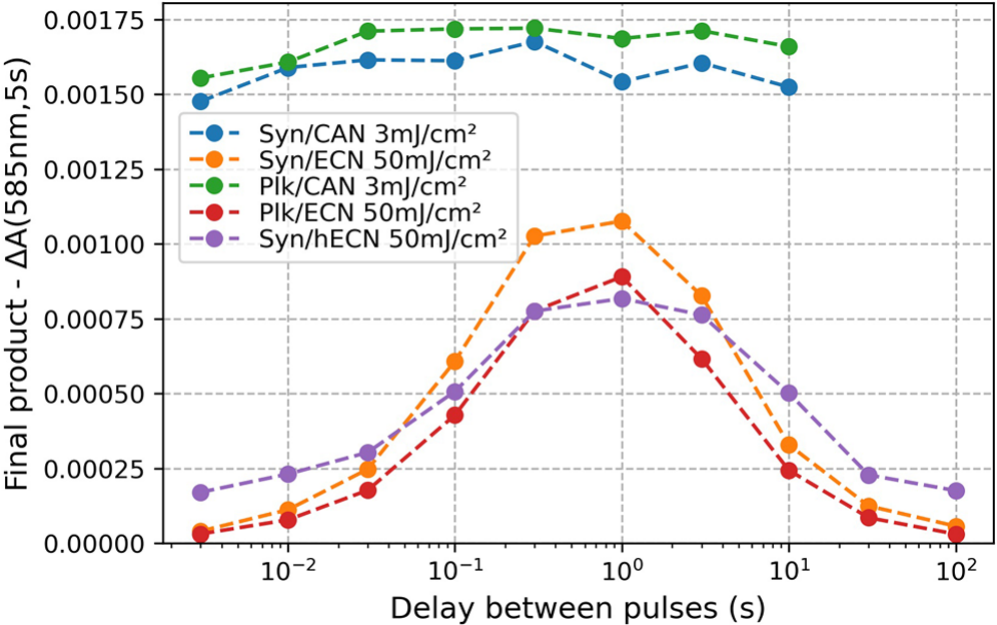
**Comparison of CAN- and ECN-functionalized OCP from*Synechocystis* and *Planktothrix***. The change in absorption at 585 nm recorded 5 s after the second pulse, representing the yield of OCP^R^, is plotted against the time delay *t*_*delay*_ between the two laser excitation pulses. It is striking that the photoresponse of CAN-functionalized OCPs differs strongly from ECN and hECN complexed OCPs. Stationary spectra of the samples used are shown in Figure S5B in the Supporting Information.

We tested whether the difference in the reaction mechanism observed for CAN- and ECN-functionalized OCP from *Synechocystis* also applies to OCP from *Planktothrix* (see Supplementary Figures S7 and S11). The dependence of the transient absorption signal at 585 nm associated with an OCP^R^-like product on the time delay *t*_*delay*_ between the two excitation pulses is shown in Figure 6. A strong maximum at *t*_*delay*_ = 1 s is observed for ECN-functionalized proteins from both cyanobacterial strains, implying that the OCP^1hν^ population is peaking. By contrast, the transient absorption signal at 585 nm does not depend on *t*_*delay*_ in CAN-functionalized proteins from both strains. For higher excitation pulse energies, there is a minor dependence of the OCP^R^ signal on *t*_*delay*_ (see Figure S20 in the Supporting Information). However, its absence in the spectra obtained after the weakest excitation pulse (3 mJ/cm^2^) makes it very unlikely that it is a manifestation of a two-photon mechanism.

Since most studies on OCP are performed using His-tagged variants we also tested whether these proteins behave analogously to the untagged proteins. Indeed, ECN-OCPs from *Synechocystis*, His-tagged at the N- and C- termini, respectively, photoconvert according to the two-photon mechanism (see Figures S21 and S22 in Supporting Information, respectively). However, we note that the spectral signatures of their OCP^1hν^ intermediates show additional positive contributions compared to the tag-free variant, resembling OCP^R^ more. In conclusion, the photoconversion mechanism is preserved among different OCP variants and depends only on the bound chromophore.

### Comparison with Kinetics Obtained upon Continuous Light Irradiation

**Figure 7:**
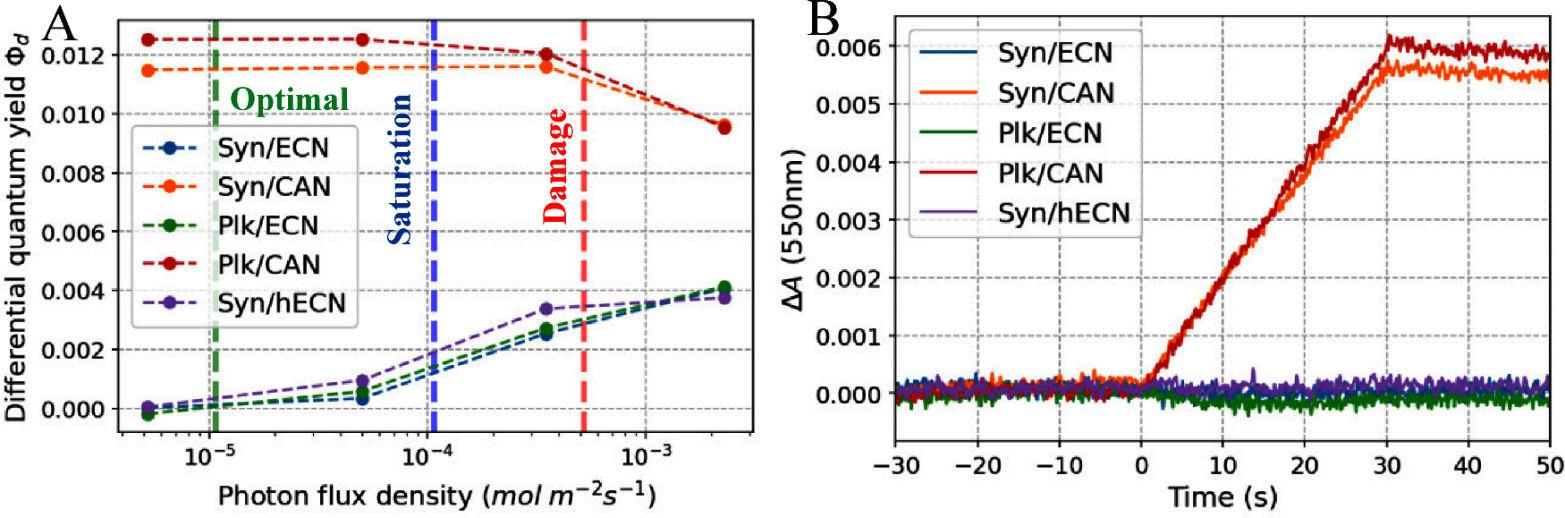
**Differential quantum yields of CAN, hECN, and ECN complexed OCP exposed to stationary irradiation.** A) We used the method described previously to derive the differential quantum yield ϕ_*d*_^29^ as a function of the photon flux density at 472 nm. Vertical lines mark “optimal”, “saturation” and “damage” photon flux densities that correspond to photosynthetic photon flux densities (PPFD) of 30, 300, and 1460 μmol photons s^–1^ m^–2^ discussed in the context of cyanobacterial photosynthesis. Note, since OCP absorbs only a fraction of the photosynthetically active radiation (PAR) the values were recalculated using a conversion factor of 2.8, more details are in the Experimental section in the Supporting Information. B) Difference absorbance kinetics at 550 nm observed upon weak irradiation using a 472 nm LED (photon flux density 5.2 × 10^–6^ mol m^–2^ s^–1^). The irradiation light is switched on at *t* = 0 and off at *t* = 30 s, respectively, the sample absorbance is A = 0.62 (10 mm path length, absorption maximum).

We wondered how these results correlate with kinetic measurements performed using continuous light irradiation. Previously we had proposed the need for a 2-photon mechanism of the OCP^O^ → OCP^R^ photoconversion^29^ based on the intensity dependence of the differential quantum yield^30^ ϕ_*d*_ of ECN-complexed OCP from *Synechocystis*. Here, we plot ϕ_*d*_ versus irradiation photon flux density for CAN, ECN and hECN-functionalized OCPs (Figure 7A). For CAN, the ϕ_*d*_ value is constant, except for high irradiation intensity where it decreases due to fast saturation of the OCP^R^ population, resulting in an early plateau (as modeled in our previous report^29^). Thus, the differential quantum yield for CAN-OCPs is invariant of the irradiation intensity, allowing to describe the OCP^O^ → OCP^R^ photoconversion by a single, well-defined quantum yield equal to ϕ_*d*_. By contrast, for ECN-functionalized OCP ϕ_*d*_ is negligible for weak continuous irradiation intensity, whereas it grows significantly for stronger continuous irradiation (Figure 7A). Strikingly, at low irradiation intensity no photoactivity is observed for ECN and hECN-OCPs compared to significant OCP^R^ accumulation for CAN-OCPs (Figure 7B). The finding that ECN-OCPs are photoactive only above a specific light intensity threshold is in line with the two pulse excitation results.

### Investigation of the Monomeric R27L Mutant

**Figure 8:**
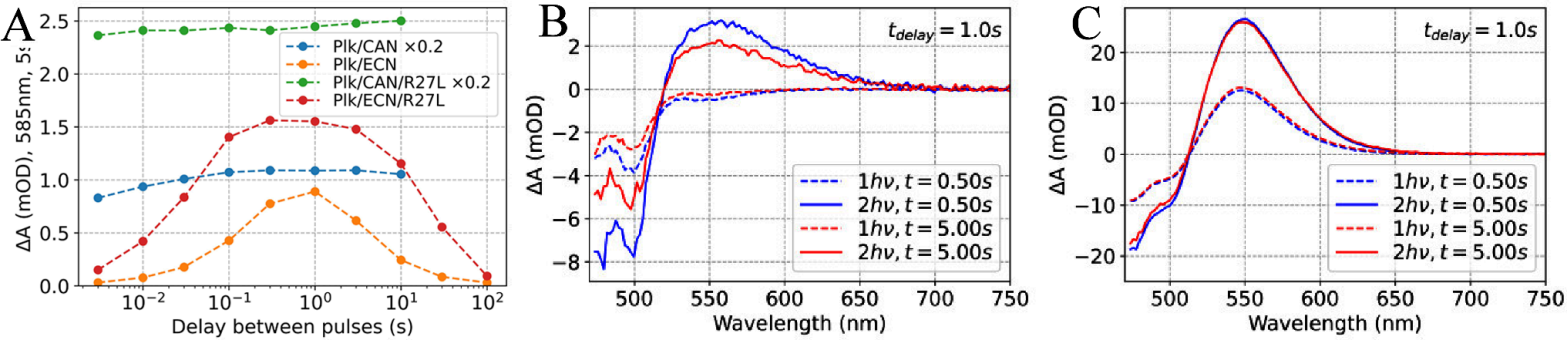
**Two-pulse excitation experiment analyzing R27L mutants without His-tag.** The transient absorption spectra were obtained after one or two 512 nm excitation pulses (50 mJ/cm^2^). A) The change in absorption at 585 nm recorded 5 s after the second pulse, representing the yield of OCP^R^, is plotted against the time delay *t*_*delay*_ between the two laser excitation pulses. The results for R27L mutants and equivalent WT proteins are shown. B) and C) Transient absorption spectra obtained after one and two excitation pulses spaced by *t*_*delay*_ = 1 s, for the Plk/ECN/R27L and Plk/CAN/R27L mutants, respectively.

OCP exists in a monomer–dimer equilibrium, with a dissociation constant of ≈14 μM.^11,41^ Since our experiments were performed at an OCP concentration of ≈60 μM and thus with mainly dimeric protein, it is conceivable that the two-photon mechanism derived for ECN-functionalized OCP is rooted in the absorption of one photon by each monomer instead of the absorption of two photons by a single monomer and thus chromophore. To distinguish between these possibilities, we investigated the R27L mutant of OCP which had been shown to be monomeric.^41^ Our size exclusion chromatography (SEC) results show a monomeric state for both ECN and CAN-functionalized R27L mutants of OCP (see Section 1.2 in the Supporting Information, Figure S30). The CAN-complexed R27L mutant photoconverts according to a single-photon mechanism, like its WT (wild-type) counterpart (Figure 8C). Compared to CAN/WT the spectral response of the CAN/R27L mutant to an irradiation pulse more closely resembles a step function. As shown in Figure 8B, the ECN-complexed R27L mutant clearly shows the distinct spectral features of the OCP^1hν^ intermediate, with OCP^R^ formation peaking for *t*_*delay*_ = 1s as observed for WT OCP (Figure 8A). Interestingly, for both chromophores the R27L mutant exhibits about 2-fold higher ΔA amplitudes after the first and second excitation pulse, respectively, compared to WT (see Figures 8A, and S27, S28 in Supporting Information for detailed data overview). In case of ECN-OCP this indicates that the presence of OCP^O^ dimers decreases the quantum yield of the OCP^1hν^ formation in the first light-induced step, while the OCP^R^ formation yield in the second step is not significantly affected. Thus, one can hypothesize that dimer dissociation into monomers must occur in order for OCP^1hν^ to be formed, if OCP^O^ dimer is irradiated. However, the requirement of two photons for photoconversion does not stem from the necessity of the dimer to dissociate, as evidenced by the data obtained for the R27L mutant. In monomeric ECN-OCP two absorption events are still required in order to yield OCP^R^. Thus, the two-photon feature is not directly associated with the monomerization reaction as proposed by Rose et al.^31^

## Discussion

**Figure 9:**
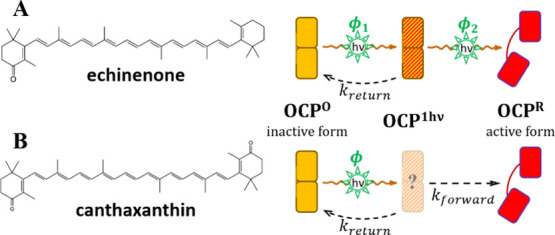
**Simplified illustration of the two-photon and single-photon photoconversion mechanisms in OCP.** A) ECN (and hECN) functionalized OCP displays a sequential two-photon photoconversion mechanism, involving two absorption events, characterized by a quantum yield ϕ_1_ and ϕ_2_, respectively. Upon absorption of the first photon by OCP^O^ a metastable intermediate OCP^1hν^ is formed with a probability ϕ_1_. Upon absorption of a second photon by OCP^1hν^, OCP^R^ is formed with a probability ϕ_2_. If the light intensity is too low to “provide” another photon within the lifetime of OCP^1hν^ it reverts thermally to OCP^O^. We note that the ϕ_1_, ϕ_2_ and ϕ quantum yields quantify the final outcome of specific cascades of thermally decaying intermediate states to OCP^1hν^ and OCP^R^, respectively. These earlier intermediates are not shown here, including an OCP^X^ form that precedes OCP^1hν^. The cascade of prior intermediates needs some finite time to decay and then form OCP^1hν^ (resulting in the bell-shaped curve in Figure 6). B) By contrast, CAN functionalized OCP is converted to OCP^R^ upon absorption of a single photon. We cannot exclude formation of an OCP^1hν^ intermediate with an absorption spectrum identical to OCP^R^ which decays thermally to OCP^R^. For simplicity, only monomeric OCP is depicted, despite the fact that OCP can be also dimeric. By analyzing the monomeric R27L mutant we show that the need for two distinct photon absorption events for the photoconversion of ECN-OCP^O^ to OCP^R^ does not depend on the oligomerization state.

We investigated the millisecond to second kinetics of the OCP^O^ → OCP^R^ photoconversion using a pump–pump–probe transient absorption setup and found that CAN functionalized OCP forms OCP^R^ upon absorption of a single photon whereas ECN and hECN functionalized OCP requires the sequential absorption of two photons. In the latter two cases the OCP^R^ yield depends strongly on the time delay between the two light pulses, peaking for *t*_*delay*_ = 1 s. This is due to formation of a metastable OCP^1hν^ intermediate that reverts thermally to OCP^O^ if no photon is available within its lifetime or, alternatively, forms OCP^R^ upon absorption of a photon with ϕ_2_ quantum yield (see Figure 9). It is thus not surprising that OCP is such an enigmatic system since the  photoconversion of ECN and hECN-functionalized OCPs is “by design” insensitive to single, short light pulses that are the standard temporally well-defined tool used in most spectroscopic experiments.

Rose et al.^31^ recently proposed a two-photon photoconversion mechanism for CAN-functionalized OCP that starts with photodissociation of dimeric OCP^O^ to yield two monomeric intermediates one of which absorbs a photon to form OCP^R^. These intermediates have been reported to have an absorption spectrum almost identical to OCP^R^, except for a 550–630 nm flank that is exclusive to the OCP^R^ form.^31^ This interpretation is not supported by our observations that favor a single photon mechanism for CAN-OCP. In our data, a species possessing a broader absorption band with a 550–630 nm flank is only observed at high pump energy density, but not at 3 mJ/cm^2^ (Figure S19, Supporting Information). Moreover, at low pump energy density the spectral properties are independent of the time delay *t*_*delay*_ between the first and the second excitation pulse. Therefore, we conclude that there is no experimental evidence to assign the occurrence of the 550–630 nm flank to the photoconversion of the OCP^1hν^ form to the OCP^R^ form. The response after only one excitation pulse has both the spectral and temporal properties sufficient to ascribe it to OCP^R^ formation. The appearance of the 550–630 nm flank is very small and can be related to nondirect effects of the irradiation intensity such as OCP^R^-OCP^R^ dimerization or light-induced changes of the equilibrium between subpopulations coexisting in the OCP^O^ or OCP^R^ forms. The situation is clearly different for ECN-functionalized OCP which does exhibit a clear sequential two-photon mechanism, involving an OCP^1hν^ intermediate with completely different spectral properties than OCP^R^. It is also clear that dimer dissociation is not a prerequisite for the two-photon mechanism since both CAN and ECN functionalized monomeric R27L-OCPs show the same mechanistic features as the corresponding WT complexes (Figure 8). Importantly, the data on the mutant show that both sequentially occurring light-induced photoconversion steps are triggered by photon absorption by the same chromophore.

Chukhutsina et al.^17^ recently reported a transient absorption study of OCP containing CAN and ECN chromophores, covering a time regime from 1 μs up to a few milliseconds. Interestingly, they identified intermediate forms characterized by a negative ΔA peak in the bleaching region, that resembles the OCP^1*hν*^ signature, with no positive peak resembling OCP^R^-like products formation for both chromophores. Figure S29 in the Supporting Information shows transient absorption spectra for the millisecond delays recorded in our study. We observed a different spectral evolution than Chukhutsina et al.; however, due to limited temporal overlap between the Chukhutsina et al. study and this work, as well as differences in sample preparation protocols, it is hard to judge whether the differences between these results are significant.

Stepwise two-photon absorption mechanisms in single chromophore molecules have been described for photochromic materials,^37,42,43^ but–to the best of our knowledge–not for biological photosensory molecules. The mechanism described here for OCP differs from the one reported for dimeric BLUF-domain containing photosensors that also require two photon absorption events for high enzymatic output yield.^44^ In the latter case, each monomer absorbs a single photon resulting in a structural change that is transmitted to the respective enzymatic output domain via the α3 helix that is part of the dimer interface. Thus, like interacting gear wheels the two α3 helices introduce cooperativity, requiring both sensors to be activated for optimal transduction and thus output. The requirement common to both two-photon mechanisms is that the transient one-photon induced species needs to be long-lived and have a large absorption cross section, so that sunlight is capable of completing both steps. Two-photon absorption mechanisms result in a nonlinear response of the overall reaction to the light intensity, generating a threshold switch. In case of OCP, it prevents the down regulation of photosynthesis at low light irradiance.

The OCP^R^ forms of both CAN and ECN-complexed OCPs (*Synechocystis* and *Planktothri*x) bind to the phycobilisome and induce fluorescence quenching.^10,45^ However, in CAN-OCPs (which accumulate OCP^R^ faster than ECN-OCP), fluorescence quenching is slightly slower and less efficient and the detachment from the phycobilisome is significantly faster than for ECN-OCPs.^10^ This suggests that the structures of the red forms of CAN- and ECN-OCPs may differ slightly, which affects the interaction with the phycobilisome. The expectation that the CAN-OCP^R^ and ECN-OCP^R^ forms are similar allows to speculate that the chromophore-mediated difference in the OCP^O^ → OCP^R^ photoreaction mechanism is rooted in the difference in energy barrier between the one-photon generated intermediate OCP^1hν^ and OCP^R^. If true, this barrier is low enough for CAN-OCP so that it can be crossed thermally, without the need for a second photon (see Figure 9).

CAN, hECN and ECN differ in the β2 ionone ring. Previously, it was reported that the presence of a β2 carbonyl group accelerates the photocycle kinetics of OCP^17^ and influences transient absorption kinetics in the milliseconds time regime.^10^ Here we demonstrate that it affects the mechanism of the photoreaction in a fundamental way. Since the carbonyl group in the β2 ionone ring in CAN is very close to Leu37 (Supplementary Figure S26A), it is conceivable that steric effects may play a role in the mechanistic differences. Therefore, we investigated the L37V and L37A mutants of OCP complexed with ECN and CAN. However, we found only a decrease in the OCP^R^ yield, without alteration of the number of photons required to form OCP^R^ (Figures S26B, S23, S24, S25 in Supporting Information). Another property that may play a significant role in the reaction mechanism is the charge distribution along the carotenoid polyene chain: carbonyl groups have an electron-withdrawing property; having one on either side of the chromophore (CAN) instead of only one (ECN) results in a different charge distribution. In the latter case the charge distribution is asymmetric (often associated with the presence of an intramolecular charge transfer (ICT) excited state). Further studies are needed to address this hypothesis and to characterize the structural features of OCP^1hν^.

In general, little is known about which carotenoid is bound to natively expressed OCPs in vivo. Only three OCPs were isolated from native cyanobacteria cells. *Arthrospira maxima*(46) and *Synechocystis* 6803 bind hECN.^3^ However, when the OCP concentration increases, for example by overexpression, these proteins also bind ECN in addition to hECN.^34,47^ By contrast, the *Tolypothrix* (*Fremyella*) OCP, which was isolated from a strain overexpressing OCP, binds CAN.^48^ When OCPs are expressed in an *E. coli* heterologous expression system in the presence of CAN and low concentrations of ECN, they all bind CAN and ECN but in different ratios: *Anabaena* OCP binds around 97% CAN, *Synechocystis* around 70–75% CAN and *Arthrospira* 50% CAN.^49^ This suggests that the affinity of OCPs for the various ketocarotenoids differs. Thus, which carotenoid(s) is bound to OCP in cyanobacteria *in vivo* depends on the strain specific carotenoids^50^ and their OCP affinities. It was also demonstrated that the OCP2 protein expressed in *Fischerella thermalis* cyanobacterium can bind another ketocarotenoid, astaxanthin, and be photoconvertible.^51^

Cyanobacteria are diverse organisms that inhabit various environments, including deep ocean layers (down to ≈150 m where there is still some residual sunlight^52^), turbid lake waters (where light can sometimes penetrate only a few millimeters through blooming waters^53^), deserts and even the atmosphere.^54^ Consequently, they need responsive photoprotective mechanisms to adapt to these highly diverse and variable conditions. The optimal photosynthetic photon flux density (PPFD) for cyanobacteria is about 30 μmol photons s^–1^ m^–2^, while oxygen production grows linearly with photon flux density up to 100 μmol photons s^–1^ m^–2^ and saturates at about 300 μmol photons s^–1^ m^–2^.^55−58^ Above these photosynthetic photon flux densities (defined as photon flux density within 400–700 nm, referred to as photosynthetically active radiation - PAR), the photosynthetic apparatus receives an excessive amount of harvested light energy that would lead to damage without photoprotective mechanisms. The corresponding values (marked in Figure 7A, for calculation details see Experimental Section in the Supporting Information) overlap very well with the “threshold” regime identified here where the two-photon OCP photoconversion mechanism becomes active. Interestingly, it was found that *Synechocystis* cyanobacteria exhibit photoinhibition above 800 μmol photons s^–1^ m^–2^ which is reversible up to 1460 μmol photons s^–1^ m^–2^.^59^ (As a reference, full sunlight PPFD at the equatorial equinox corresponds to 2200 μmol photons s^–1^ m^–2^.^60^) Therefore, it seems that ECN and hECN-functionalized OCPs are perfectly evolved to moderate the photoprotective function in cyanobacteria. However, it is conceivable that the various carotenoids modulate the “dynamic range” of photoprotection performed by OCPs, thereby extending the range of ecosystems available for cyanobacteria.

The possibility of selecting between a two or a single-photon photoconversion mechanism in OCP by using the proper carotenoid bears implications for potential artificial applications of OCP. It has already been shown that OCP can be utilized as an illumination intensity-controlled energy transfer modulator between two dyes interconnected by a DNA scaffold serving as an artificial antenna system.^61^ In another report, OCP2 deprived of the linker connecting both protein domains was shown to control gene transcription in chloroplasts operated by the plastid-encoded polymerase (PEP).^62^ OCP can also be used as photochromic material when immobilized in a mesoporous silica matrix.^63^ In principle, one could envision a parallel application of both two-photon and one-photon triggered photoswitches, where use of a sufficiently short light pulse will selectively trigger only the single-photon response. This way, it would be possible to jointly activate both effectors or only one of them, depending on the irradiation pulse pattern.

## Conclusions

The photoprotective process mediated by OCP is necessary in harsh irradiation conditions because it leads to safe dissipation of excessive excitations which otherwise would flood the photosystems. This, in turn, due to their inability to utilize these excitations productively, would result in large amounts of toxic reactive oxygen species. From an evolutionary point of view, it makes sense to optimize a sensor-protein like OCP such that its efficiency is low, and nonzero only if the triggering light is sufficiently intense and persistent for a long enough time to induce damage. In conditions of weak irradiation, activation of the photoprotective mechanism mediated by OCP is disadvantageous because the presence of the active OCP^R^ species dissipating scarce light energy could lead to starvation. In consequence, the OCP photoactivation mechanism should be as selective as possible with respect to the irradiation intensity. The linear response observed in case of CAN-functionalized OCP will lead to some percentage of active OCP^R^ even in low light conditions, when the dissipation mechanism is generally biologically unfavorable. By contrast, the two-photon photoconversion mechanism associated with hECN and ECN fulfills the selectivity requirement significantly better. As one can see from the continuous irradiation kinetics (Figure 7B), at low light conditions no OCP^R^ is formed, simply because the absorption events are so rare that the probability of the intermediate OCP^1hν^ form absorbing a second photon is extremely low before it relaxes back to OCP^O^. Only beyond a certain photon flux density threshold does this probability increase enough to obtain a significant OCP^R^ population. We show that ECN-OCP and hECN-OCP effectively photoconvert only above a certain photon flux density that agrees with the light conditions where cyanobacteria require photoprotection (Figure 7A). Our data demonstrate clearly that any scheme attempting to fully describe the OCP^O^ → OCP^R^ photoconversion of ECN or hECN-functionalized OCPs must incorporate at least two reaction quantum yields. It is striking that even after a strong laser pulse of 50 mJ/cm^2^ energy density, allowing for multiple reexcitations, the system cannot undergo an observable photoconversion to the final OCP^R^ product. Remarkably, the latter only occurs upon two excitation pulses delayed by approximately 1 s. To the best of our knowledge, the finding of a sunlight-induced two-photon triggered biologically relevant process occurring in a monomeric protein molecule is unprecedented.

## Data Availability

Data will be provided upon reasonable requests. The custom written code has been deposited at https://github.com/Stahux/MultipulseTransientAbsorption.

## Supporting Information Available

Experimental section, a general overview of the data discussed in the main text, and also control data (PDF)

## Abbreviations:

NPQ: nonphotochemical quenching
OCP: Orange Carotenoid Protein
OCP^O^: Orange form of the Orange Carotenoid Protein
OCP^R^: Red form of the Orange Carotenoid Protein
OCP^1hv^: Intermediate form of the Orange Carotenoid Protein
ECN: echinenone
CAN: canthaxanthin
hECN: 3′-hydroxyechinenone
PPEF: Photosynthetic Photon Flux Density
PAR: Photosynthetically Active Radiation
WT: wild-type
FWHM: Full Width at Half Maximum
