# Supplementary material for: Two-Photon-Driven Photoprotection Mechanism in Echinenone-Functionalized Orange Carotenoid Protein
Source: J Am Chem Soc. 2025 Jan 21;147(5):4100–10. doi: 10.1021/jacs.4c13341 (PMC11803624; doi:10.1021/jacs.4c13341)
Supplement: Supplementary file 1 — ja4c13341_si_001.pdf [file ja4c13341_si_001.pdf]

Supporting Information

Two-photon driven photoprotection mechanism in echinenone-functionalized Orange Carotenoid Protein

Stanisław Niziński[1]*, Elisabeth Hartmann[1], Robert L. Shoeman[1], Mirosław Tarnawski[1], Adjélé Wilson[2], Jochen Reinstein[1], Diana Kirilovsky[2], Michel Sliwa[3,4], Gotard Burdziński[5], Ilme Schlichting[1]

[1]Max Planck Institute for Medical Research, Jahnstr. 29, 69120 Heidelberg, Germany
[2]Université Paris-Saclay, CEA, CNRS, Institute for Integrative Biology of the Cell (I2BC), 91198 Gif-sur-Yvette, France
[3]Univ. Lille CNRS UMR 8516 LASIRE Laboratoire de Spectroscopie pour les Interactions, la Réactivité et l'Environnement, Lille, 59 000 France
[4]LOB, CNRS, INSERM, École Polytechnique, Institut Polytechnique de Paris, 91120 Palaiseau, France
[5]Quantum Electronics Laboratory, Faculty of Physics and Astronomy, Adam Mickiewicz University, Poznań, Uniwersytetu Poznańskiego 2, Poznań 61-614, Poland

*Corresponding author: stanislaw.nizinski@mr.mpg.de

Table of Contents

1. Experimental section 2
 1.1 Sample preparation 2
 1.2 Size exclusion chromatography 2
 1.3 Multi-pulse millisecond transient absorption spectroscopy setup 3
 1.4 Absorbance changes upon continuous irradiation determined in a stationary UV-vis spectrometer 6
 1.5 Conversion between PAR and monochromatic photon flux density 9
 1.6 Sample heating by the pump laser pulses 10
2. Synopsis of the transient absorption spectra shown in the Supplementary Information 11
 2.1 Influence of pump pulse parameters – energy density, wavelength, number of pulses 11
 2.2 Results obtained on modified proteins – tags, mutations 13
3. Decomposition of the experimental datasets 53
4. Supplementary References 54

1. Experimental section

1.1 Sample preparation

CAN and ECN functionalized OCP from *Synechocystis* and *Planktothrix* was expressed and purified as described recently[1]. hECN functionalized OCP was expressed as C-terminal His-tagged OCP in *Synechocystis* PCC 6803 and purified as described[1]. The sample was stored at -80 °C for more than 15 years, with two thawing events during this time. Nevertheless, the protein is functional. To allow removal of the N-terminal poly-histidine tag we introduced a TEV cleavage site right after the last histidine of the tag (protein sequence MGSSHHHHHHENLYFQ↓SSFTV), resulting in an additional serine residue before the N-terminal start of the native sequence (SSFTV). The genes for the wildtype constructs containing the additional residues introducing a TEV cleavage site after the poly-histidine tag and the L37V and L37A mutant variants were ordered from Eurofins and cloned into a pCDFDuet™ vector. We ordered the entire genes for the *Synechocystis* variants. For the *Planktothrix* variants, we exchanged the wildtype sequence between the Nco and Hind III restriction sites of the OCP gene with the synthesized (modified) parts, coding for the additional residues for the TEV cleavage site (see above) and the L37V and L37A mutations, respectively. The correctness of the new constructs was verified by sequencing. All samples used in the two-pulse experiments were concentrated to 20 mg/mL in a buffer containing 40 mM Tris-HCl pH 8.0, 25 mM NaCl. An extinction coefficient of ε_{500nm} ≈119,000 M^{-1} cm^{-1} was used for protein concentration determination.

1.2 Size exclusion chromatography

Analytical size-exclusion chromatography (SEC) experiments were performed to investigate the oligomeric state of wild-type (WT) and R27L mutant OCP and to test whether it depends on the bound chromophore. The protein samples were analyzed on a Superdex 200 Increase 10/300 GL column (Cytiva) equilibrated with 50 mM Tris-HCl pH 8.0, 150 mM NaCl buffer at a flow rate of 0.5 ml/min. 50 µl of each sample at three different protein concentrations (10, 50 and 200 µM) were injected and the UV absorbance at 280 and 260 nm was monitored. Gel Filtration Standard (Bio-Rad) was used to calibrate the column and determine the apparent molecular weight. Figure S30 shows the elution profiles obtained for WT proteins (with His-tag removed) and R27L mutants functionalized with ECN or CAN chromophores. A strong dependence of the elution volume on the protein concentration was observed for both OCP WT proteins. This indicates that the WT proteins form dimers and are in monomer-dimer equilibrium with an exchange rate faster than the experimental timescale, resulting in a single broader peak. By contrast, the elution volume of the R27L proteins was unaffected by protein concentration and corresponds to a stable monomer independent of the chromophore. These results show that the monomer/dimer ratio of the R27L mutant OCP is not protein concentration dependent in the 10 µM to 200 µM concentration range, in contrast to the wild-type OCP. Essentially, there is no difference between the CAN and ECN chromophores, demonstrating that the observed two photon photoconversion mechanism is not a consequence of the different monomer/dimer ratios for different chromophores.

1.3 Multi-pulse millisecond transient absorption spectroscopy setup

The experimental setup is based on two noncollinear beams, one used as probe and the second serving as excitation pulse (Figure S1 shows the geometry of the setup.). Continuous probing light is generated by a Thorlabs MBB1F1 fiber-coupled LED (spectral range 470 - 850 nm), fed through a 200 μm SMA fiber (M92L02, Thorlabs), recollimated using a Thorlabs RC12SMA collimator, cut by an aperture, and focused to a 134×124 μm spot (H×V, FWHM) in the cuvette. The aperture blocks the majority of the probe light before the sample, ensuring very low intensity probing conditions. Figure S2B shows an intensity spectrum of the probing light. The measured probe power was about 1.5 μW (measured with a Gentec PH100-Si-HA detector head, measurement wavelength setting at 530 nm); however, note that only a fraction of this light overlaps with the OCP^{O} absorption spectrum, ensuring accurate measurement without perturbing the photo-activation dynamics (Figure S2B). The probe light is collected and recollimated through a AC127-030-A-ML lens after the sample and fed to a thick multi-fiber SMA optical fiber using a F810SMA-543 collimator. This fiber is terminated at the entrance slit of an Andor spectrometer coupled with a Newton Peltier-cooled camera operating in unamplified mode (1600 pixels, 150 groves/mm grating). The probe light is efficiently collected, and its intensity adjusted such that the camera operates much below saturation threshold. The camera is operated in full vertical binning mode.

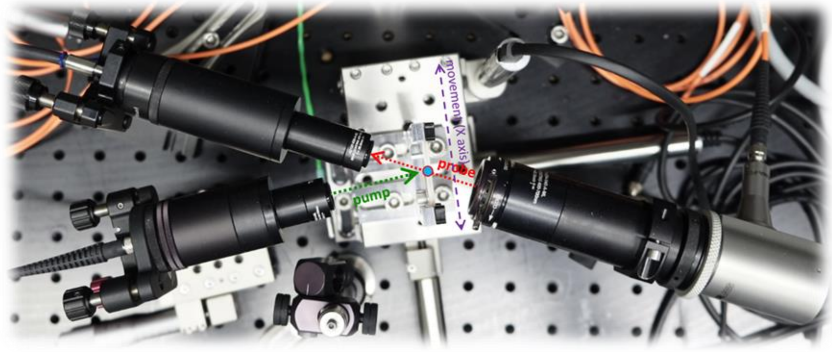

Figure S1. Top-view of the experimental setup geometry.

Pump pulses are produced by an Oxxius LBX-520-800-HPE-PP laser diode module, fed to the SMA 200 μm fiber (M92L02, Thorlabs), then recollimated using a F810SMA-543 collimator and focused in the sample using an AC127-050-A-ML lens, yielding a 193×185 μm spot (H×V, FWHM). These pulses are triggered by a TTL trigger; the length determines the pulse energy. The temporal shape of this pulse is fast-growing followed by an exponential decay with a time constant of ≈ 290 μs, resulting in a pulse duration of about 200 μs (FWHM).

A 100 μm pathlength quartz cuvette (Hellma) is mounted using a custom-made aluminum magnet-secured holder (see Figure 1A in the main text) which guarantees that the sample is always in the plane of the XY stage motion (the third axis is always fixed at the probe/pump focal plane) and that the sample mounting repeatability is very high. The experiment is performed in complete darkness at 22±1°C, stabilized by air conditioning.

All triggers (camera trigger, laser pulse trigger and probing LED trigger) are generated by the ATmega32U4 microcontroller located on an Arduino board. A special procedure written in C uses internal counters in order to generate a proper trigger sequence for the camera and laser module with sufficient temporal

precision dictated by onboard quartz oscillator (16 MHz). A custom Python-written procedure executes the experimental sequence, including movements of the XY axes and communicating the proper trigger setting to the Arduino board before each kinetic is being recorded. The Andor Solis software runs independently in a loop and after each camera trigger saves the acquired kinetic into a *.sif file marked by incremental number.

The experimental sequence consists of 4 cycles executed repeatedly for a sequence of delays t_{delay} (see Fig. S2A). Delays of 3 ms, 10 ms, 30 ms, 100 ms, 300 ms, 1 s, 3 s, 10 s, 30 s, 100 s were used. In the first cycle, the probing light is switched on and a kinetic time course is collected (spectra recorded with sampling period 2.61 ms). In the second cycle, a kinetic time series with one pump pulse is collected (the pump pulse is generated 2.5 s after the probe is switched on). In the third cycle, two pulses with predetermined t_{delay} between them are used to pump the sample. The second of these pulses is aligned such that it occurs 2.5 s after switching on the probe light, meaning that for long t_{delay} delays only the evolution after the second pulse will be recorded. This is necessary, otherwise, there would be a large effect of probe exposure for long t_{delay} delays. Last, the fourth cycle is dark, no pump or probe light is present. All generated pulses are identical.

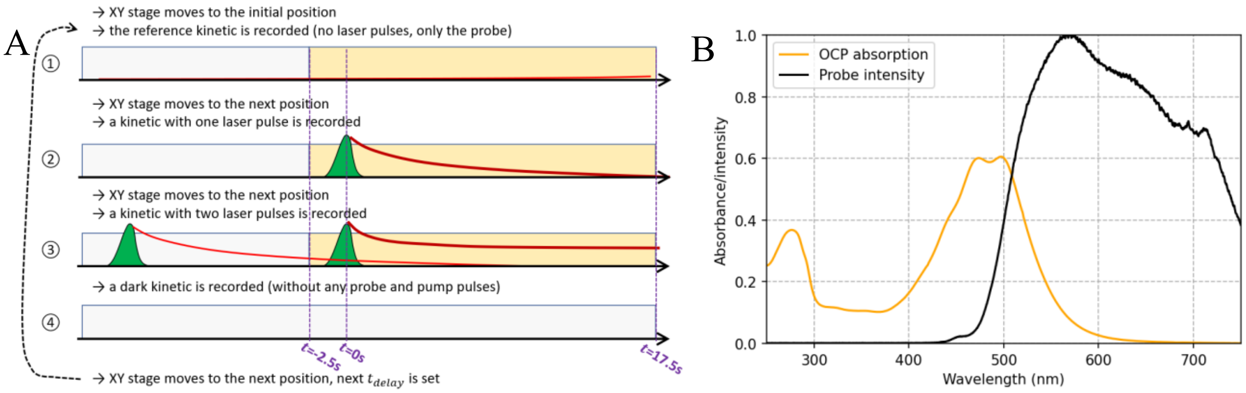

Figure S2. A) The experimental sequence consists of 4 steps performed repeatedly for a grid of t_{delay} values. Yellow background marks the presence of the probing light, grey background marks moments when the probe is off. t_{delay} is defined as the time between both excitation pulses (green) in cycle ③. B) Normalized spectrum of the probing light and typical stationary absorption spectrum of OCP (in the 100 μm cuvette).

These 4 cycles are repeated multiple times for each of the 10 delays investigated (3 ms, 10 ms, 30 ms, 100 ms, 300 ms, 1 s, 3 s, 10 s, 30 s, 100 s) such that after the last delay the system returns to the first one, and continues until a sufficient amount of data is accumulated (usually 1000-2500 cycles in total). After each cycle, the probe and pump are-switched off and the sample XY stage is translated by 1 mm (or to the next row separated vertically by about 500 μm) in order to always probe a fresh sample aliquot (except after the 3rd cycle because the sample does not need to be refreshed to record a correct dark kinetics). Because the sample surface is limited, the stage will revisit the same place after a very long time (typically slightly more than 3 hours and 30 minutes).

This sequence was designed so that a large amount of control data is recorded; in particular 10 times more data is recorded for 1-pulse excited kinetics than for 2-pulse kinetics, which is important in data post-processing.

Each kinetics dataset starts 2.5 s before the first pulse (or the second pulse in case of step ③) and ends 17.5 s after this pulse, so that the entire time window is 20 s. We limited the experiment to this time period because the absence of a probe influence on the data cannot be guaranteed for longer time spans. The probe intensity was carefully minimized; however, it is still a focused beam, and it must have non-negligible intensity to obtain good enough spectra. Unfortunately, when using continuous probing, the probe effects accumulate over time (especially in a pulse-insensitive system like OCP) whereas pulse-induced effects are imprinted only once or twice on the sample. The probe light spectrum was selected such that it has a very small amount of light below 520 nm to avoid interaction with the sample. The disadvantage of this approach is the poor quality of the spectra at the carotenoid bleaching region.

The recorded data are binned and averaged to calculate: i) one dark kinetic from step ④, ii) one reference kinetic from step ① (only probe no pump), iii) one single pulse kinetic from step ② (probe and one pump pulse), iv) 10 kinetics recorded for each t_{delay} in step ③. The following grid of t_{delay} values was used 3 ms, 10 ms, 30 ms, 100 ms, 300 ms, 1 s, 3 s, 10 s, 30 s, 100 s. Before averaging, the intensities were median-filtered (exemplary diagnostics are shown in Figure S3 in Supporting Information).

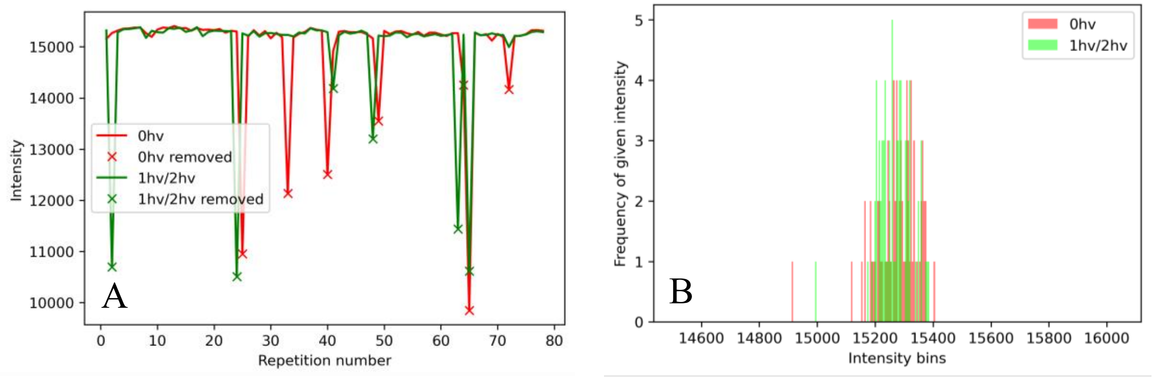

Figure S3. Exemplary diagnostics graphs (routinely checked for all datasets) demonstrating the effect of median filtering. The graphs show superimposed data obtained from cycles with one excitation pulse and cycles with the probe only. The plotted probe intensity was recorded at 700 nm, about 8 ms after triggering the camera acquisition (*i. e.* before the excitation pulse is generated). A) Negative spikes are due to small air-bubbles formed within the cuvette that act like a spherical lens and defocus the probe beam, causing a signal drop. Data points removed from the kinetic series are marked by “X”. B) Histogram of signal intensities centered at the median (distant outliers fall outside of the graph window).

The averaging procedure takes probe light intensity at 700 nm, composes the distribution of this value for all kinetics collected in given bin and calculates the median value. Then, averaging is done using all kinetics except those that deviate by more than 5% from the median. This kind of filtering allows to discard data from air bubbles that form after some time in the cuvette. Usually no more than 10% of the kinetic time

points were discarded this way. After binning, the averaged intensities of the light are used to calculate the transient absorption signal after a single and two or more pulses using the formula:

$$\Delta A_{1hv}(\lambda, t) = -log_{10}\left(I_{1\ laser\ pulse}(\lambda, t)/I_{no\ laser}(\lambda, t)\right) \quad (1)$$

$$\Delta A_{2hv}(\lambda, t, t_{delay}) = -log_{10}\left(I_{2\ laser\ pulses}(\lambda, t, t_{delay})/I_{no\ laser}(\lambda, t)\right) \quad (2)$$

The above formula assumes that dark intensities were already subtracted. The transient absorption obtained for t_{delay} delays up to 10 s are zeroed at 100 ms before the first pulse in a given cycle. Zeroing is done to compensate for long-term probe drifts occurring during the experiment and random differences in sample absorption in the different parts of the cuvette. In kinetics involving two pulses (step ③), both pulses are within the probing window only for t_{delay} < 2.5 s. Therefore, for longer t_{delay} delays, the 1-pulse kinetic (recorded in step ②) is used to correct the two-pulse kinetic properly, to ensure that ΔA = 0 is set 100 ms before the 1st pulse. Unfortunately, for t_{delay} >17.5 s (t_{delay}=30 s and 100 s) such correction is not possible, because the 1-pulse kinetics is too short. Therefore, for these two kinetics ΔA = 0 was exceptionally set 100 ms before the 2nd pulse. In other words, in those two cases we used the absorbance before the 2nd pulse as approximation of the absorbance before the 1st pulse. Based on the one pump pulse data, one can see that the error introduced by this approximation is virtually zero for probe > 525 nm in ECN-OCP and hECN-OCP proteins. Therefore, this correction does not affect absorbance at 585 nm which is the most relevant parameter. There is some residual negative signal for wavelengths < 525 nm remaining at 17.5 s after the pump pulse, which slightly affects the shape of the calculated spectra below 525 nm produced for t_{delay}=30 s and 100 s. In CAN proteins, there are long-living products even after a single pulse. Therefore, only kinetics with t_{delay}≤10 s are presented.

Finally, the signal derived from buffer is subtracted from the ΔA OCP sample kinetics to remove any effects unrelated to the protein itself. In conclusion, the experiment was designed to consider all tradeoffs and fulfill all criteria necessary to obtain reliable kinetics for OCP in the temporal window investigated (long sample relaxation time, practical absence of sample diffusion and sufficient S/N ratio).

1.4 Absorbance changes upon continuous irradiation determined in a stationary UV-vis spectrometer

Experiments with continuous light irradiation of OCP were performed as described in Niziński et al.[2]. The differential quantum yield[3] was calculated using the following formula[2]:

$$\phi_d \approx \frac{dA_{probe}}{dt} \frac{1}{I_0\ l_{probe}\ \Delta\epsilon_{OCP^R}\ F_{t=0}\ A_{irr,t=0}} \quad (3)$$

Where the photokinetic factor F is defined as:

$$F = (1 - 10^{-A_{irr}})/A_{irr} \quad (4)$$

A_{irr} is the sample absorbance at the irradiation (LED) wavelength (with 4 mm irradiation pathlength), $\Delta\epsilon_{OCP^R}$ is the differential molar absorption coefficient defined as $\Delta\epsilon_{OCP^R} = \epsilon_{OCP^R} - \epsilon_{OCP^O}$, l_{probe} is the probing path length (10 mm), I_0 is the photon flux density of the irradiation LED, A_{probe} is the absorbance measured using a 10 mm probing path length. Because the used LED (Thorlabs M470L5) has a broad

spectrum, the $I_0\, F_{t=0}\, A_{irr,t=0}$ term was integrated over the LED spectrum and the OCP^O absorption spectrum, respectively, to take into account different absorbances at different spectral components of the irradiation light. Therefore, the above equations can be rewritten as:

$$\phi_d \approx \frac{dA_{probe}}{dt} \frac{1}{l_{probe}\, \Delta\epsilon_{OCP^R}\, I_0 \int \; (1 - 10^{-A_{irr}(\lambda)})\, S_{LED}(\lambda) d\lambda} \quad (5)$$

Where $S_{LED}(\lambda)$ represents the normalized spectrum of the Thorlabs M470L5 LED, so that $\int \; S_{LED}(\lambda) d\lambda = 1$. Nevertheless, the difference in correction obtained using eq. 5 vs eq. 3 is very minor. A quartz cell of 4 × 10 mm optical path lengths was utilized (applicable for irradiation and probing, respectively), the sample temperature was stabilized at 22±1 °C. The irradiation LED light was collimated and directed towards the sample (perpendicular to the probing beam). The lower irradiation path length (4 mm) results in lower absorbance in the irradiation beam, while keeping a higher absorbance in the probing beam, so that the observed absorbance amplitudes are also high, but light intensity gradient along the irradiation beam is low (so sample mixing does not affect the kinetics). The geometry of the irradiation was set so that the whole sample volume is uniformly irradiated by the LED (however, only a fraction of the volume is being probed). A Jasco V-650 spectrophotometer was used for probing with an additional 550 nm bandpass filter placed after the sample cuvette to remove scattered irradiation light. 2 nm probe bandwidth was used in a low stray light mode. The reference beam has been blocked from the irradiation light, and no reference cuvette was placed there. Each kinetics was recorded with 30 s irradiation time, with 10 Hz sampling frequency. A fresh aliquot of the dark-adapted sample was used for each experiment repetition. The full stationary absorption spectrum (in the 250 nm - 750 nm range) was recorded without the bandpass filter in the probing beam, before each kinetic to ensure that initial conditions for each experiment repetition are well defined. A linear regression was performed using the linear part of each kinetics (see Fig. S4). In case of high irradiation intensity, the recorded absorption signal starts to saturate for some kinetics after only a few seconds of irradiation (see Figure S4D). Therefore, in this case only the initial datapoints were used. In case of lower irradiation intensities, for a few seconds after the irradiation started, the absorbance was growing linearly. Linear fits were performed using the lmfit Python package, and the slope obtained (dA_{probe}/dt) was used to calculate ϕ_d using equation 5. The irradiation intensity was determined using a Gentec PH100-Si-HA detector head. Normalized stationary spectra of the samples used are shown in Figure S5A.

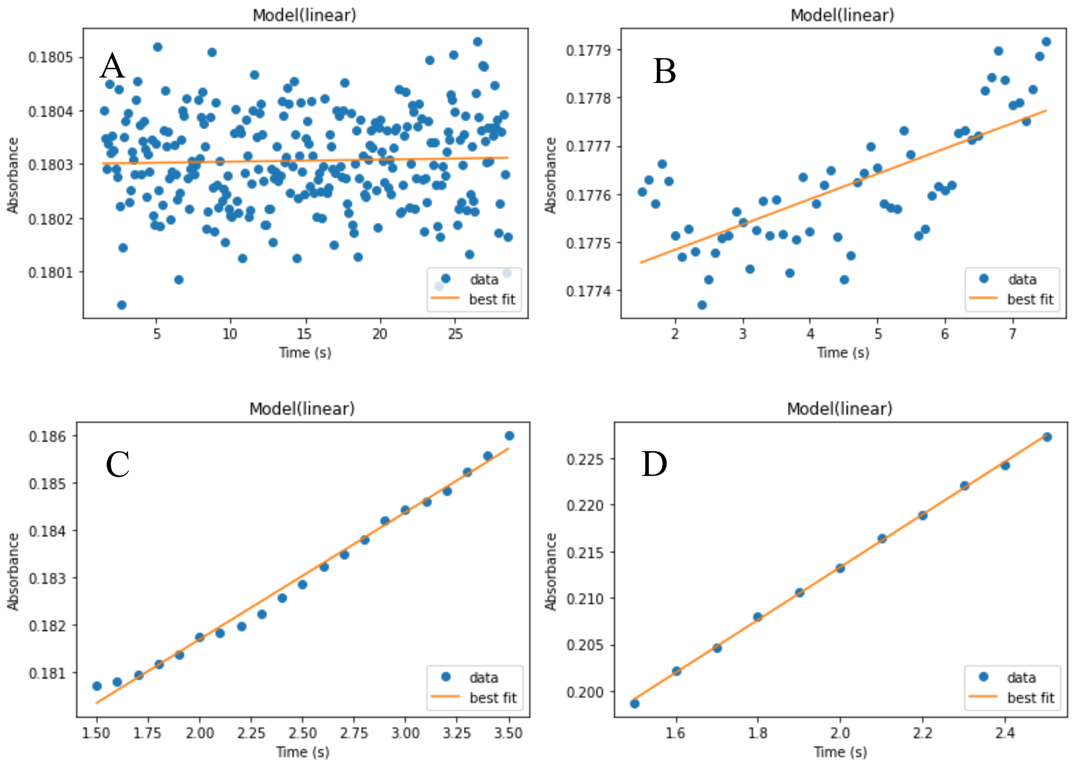

Figure S4. Exemplary linear regression fits of the absorbance during the first seconds of irradiation of a Syn/ECN sample. The irradiation intensities are A) 5.22, B) 50.3, C) 348, D) 2289 µmol photons s^{-1} m^{-2}.

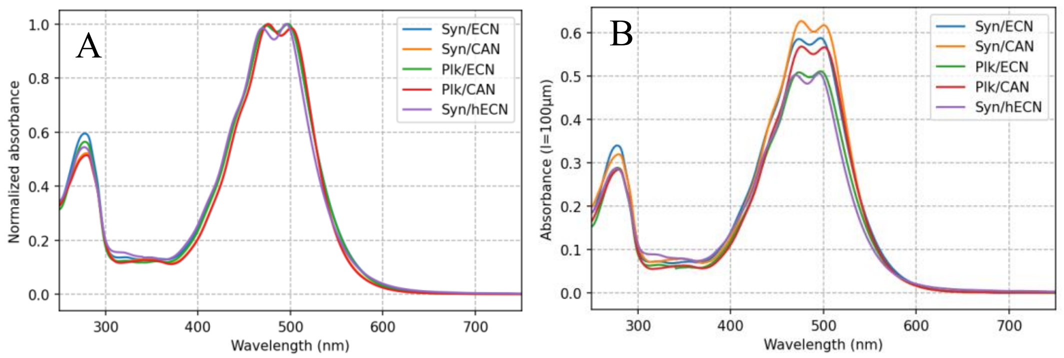

Figure S5. UV-vis absorption spectra. A) Normalized UV−vis stationary absorption spectra of OCP samples used to calculate their respective differential quantum yields[2]. The measurement was performed using a sample in a 4×10 mm cuvette. B) Unnormalized spectra of the samples as used in the two-pulse excitation experiment, measured in a 100 µm pathlength cuvette (just before the experiment).

Maksimov *et al.*[4] reported a molar absorption coefficient of 87 270 M^{-1} cm^{-1} (495 nm) for ECN-functionalized OCP from *Synechocystis* (using a triple mutant devoid of tryptophans except one, Trp-288). Using this molar absorption coefficient, we derive a differential molar absorption coefficient of $\Delta\varepsilon$(550 nm) = 29525 M^{-1} cm^{-1} (taken from Figure 1 in the main text).

1.5 Conversion between PAR and monochromatic photon flux density

In the cyanobacteria literature, the light intensity is quantified using the unit of photosynthetic photon flux density (PPFD) which is a measure of photosynthetic active radiation (PAR). It takes into account only sunlight in the 400-700 nm spectral range. In the experiments described here we used a 472 nm LED to irradiate the sample. Its spectrum is much narrower (however not monochromatic) compared to the spectrum of sunlight in the PAR range. Thus, because the majority of the PAR radiation falls outside the carotenoid absorption spectrum, a direct comparison between the PPFD values reported in the literature and the photon flux densities given here is not meaningful. In order to calculate a conversion factor between these values, we first calculated the photosynthetic photon flux density (assuming PAR spectral range) that results in absorption of 1 photon per carotenoid per second on average. Then, we calculated the photon flux density assuming 472 nm LED light, resulting in the same condition of 1 photon absorption per carotenoid per second. The ratio between these values yields the desired conversion factor. It allows to compare the literature values describing cyanobacteria functioning with the values obtained here describing OCP functioning. For PAR, we assume a standard AM1.5G spectrum[5] (given in W m^{-2} nm^{-1} as a function of wavelength) truncated to the 400-700 nm range and recalculated into mols photons m^{-2} nm^{-1} and normalized to unity, so that:

$$S_{PAR}(\lambda) = \frac{AM1.5G(\lambda)\frac{\lambda}{h\,c\,N_A}}{\int_{400nm}^{700nm} AM1.5G(\lambda)\frac{\lambda}{h\,c\,N_A}\,d\lambda} \tag{6}$$

Where h is the Planck constant, c the velocity of light, N_A the Avogadro number. A similar normalized spectrum $S_{LED}(\lambda)$ was calculated for the 472 nm LED source (Thorlabs M470L5). Both normalized spectra have units of nm^{-1}. The number of photons absorbed per molecule per second is calculated using formula:

$$N^{PAR} = \int_{400nm}^{700nm} ln\,ln\,10\, I^{PAR}\, S_{PAR}(\lambda)\, \varepsilon(\lambda)\, d\lambda \tag{7}$$

Note that the photosynthetic photon flux density I^{PAR} (mol m^{-2} s^{-1}) and molar absorption coefficient of the carotenoid $\varepsilon(\lambda)$ (mol^{-1} dm^3 cm^{-1}) are defined using different units, so an additional factor of 0.1 is necessary to ensure proper unit conversion. Using these equations one can calculate a conversion factor between PPFD and the photon flux density of the 472 nm LED by assuming $N^{PAR} = N^{LED}$:

$$\frac{I^{PAR}}{I^{LED}} = \frac{\int_{400nm}^{700nm} S_{LED}(\lambda)\,\varepsilon(\lambda)\,d\lambda}{\int_{400nm}^{700nm} S_{PAR}(\lambda)\,\varepsilon(\lambda)\,d\lambda} \tag{8}$$

It allows to convert between the photon flux densities given for both types of radiation, ensuring that the considered quantities are effectively the same from the carotenoid's perspective, *i. e.* they result in the same number of photons absorbed. It can be calculated that for a PAR spectrum and photosynthetic photon flux density of I^{PAR}=148 µmol s^{-1} m^{-2} there is one photon absorbed per molecule per second on average (so that N^{PAR}=1 s^{-1}). For the 472 nm LED spectrum (Thorlabs M470L5), and photon flux density of I^{LED}=54 µmol

s^{-1} m^{-2} there is also one photon absorbed per molecule per second on average (so that N^{LED}=1 s^{-1}). The conversion factor calculated using formula (8) is equal to I^{PAR}/I^{LED}=2.8. It means that a 2.8-fold lower photon flux density is needed when using the LED compared to sunlight to result in the same number of photons absorbed.

1.6 Sample heating by the pump laser pulses

Strong irradiation pulses deposit a lot of energy in the sample which may result in sample heating. Ignoring the Gaussian spatial profile of the pump pulse, an area of 193 µm · 185 µm ≈ 0.036 mm^2 is excited. The sample layer thickness is 100 µm, therefore the excited volume is approximately 0.0036 mm^3 = 0.0036 µL. Assuming that the sample's density is close to water's density, this corresponds to a weight of 3.6 · 10^{-9} kg. When assuming a pulse energy density of 50 mJ/cm^2 and no pulse transmission through the sample, 1.8 · 10^{-5} J will be deposited in the excited volume. Assuming a specific heat capacity of 4184 J kg^{-1} K^{-1} (ignoring the non-water content of the sample), the temperature increase of the sample is about 1.2 °C after one excitation pulse. This value is not negligible, but much too small to significantly alter the sample properties, including its kinetics. It also cannot explain the observed "memory" effect of OCP, in particular when the excitation pulses are separated temporally by several seconds. The heated sample aliquot is surrounded by glass and "cold" sample solution, therefore efficient cooling will lead to fast heat dissipation. Both the ECN-OCP and CAN-OCP samples are almost identical from the thermal point of view, however they exhibit completely different behavior after the strong excitation pulse.

2. Synopsis of the transient absorption spectra shown in the Supplementary Information

The data shown in this section belong to experiments described in the main text, such as investigations on OCP from *Planktothrix* or mutant variants, but also control studies addressing the influence of the pump laser energy density or the wavelength of the pump pulse. In the following text, proteins from *Synechocystis/Planktothrix* will be abbreviated as Syn/Plk, followed by their chromophore; e.g. Syn/ECN is OCP from *Synechocystis* complexed with echinenone.

Similar to the figures shown in the main text, the figures depict transient absorption spectra recorded 500 ms and 5 s after the single pump pulse (dashed lines) and after the second of the two pulses (continuous lines, always for a specific t_{delay}). For each dataset, we also show kinetics probed at 585 nm after two pump pulses and transient absorption spectra recorded after a single pump pulse for various delays. The probing time window extends to 17.5 s after the laser pulse. We show only data up to 5 s, for which we are certain that no significant probe effect is present. However even in the full 20 s probing time window, effects of the probe are rather minor (see Figure S6). Since the probe is continuous, its effects accumulate in the sample over an extended period of time (t >> 5 s). The goal of these experiments was to capture effects of the photoconversion, not the decay kinetics. In order to record the full decay kinetics, it may be advantageous to use single probe pulses to completely avoid probe effects. This, however, will require significantly more repetitions of the experiment, which is why we used continuous probing.

2.1 Influence of pump pulse parameters – energy density, wavelength, number of pulses

Figures S11, S12 and S13 present data obtained for Plk/ECN using pump laser energy densities of ~50 mJ/cm^2, ~12 mJ/cm^2 and ~3 mJ/cm^2, respectively. One can see that when the laser energy density increases from 3 mJ/cm^2 to 12 mJ/cm^2, the 490 nm band intensity only doubles and stays almost the same when increasing the energy density from 12 mJ/cm^2 to 50 mJ/cm^2. This indicates that either the OCP^O population is almost completely depleted by the 50 mJ/cm^2 excitation pulse, or, alternatively, that there is a limiting process that prevents the full transition of the OCP^O population to the OCP^{1hv} form. Note that for all excitation energies, the second pulse is always capable of doubling the 490 nm signal amplitude present after the first pulse. This observation supports the second possibility – there must be some process limiting the number of molecules photoconverted to the OCP^{1hv} form per time unit. It is clear that this process acts on the submillisecond time scale (the laser pulse duration), but ceases to occur at longer timescales ($t \geq$ 3ms – the shortest used t_{delay}). This could be an additional inactive dark form in which the majority of the photoexcited OCP is trapped which needs time to decay and that otherwise cannot productively photoconvert towards OCP^{1hv}. Alternatively there may be a preferred back photoconversion pathway between an intermediate preceding OCP^{1hv} (which we denote as OCP^X) and OCP^O, so that the OCP$^X \overset{hv}{\rightarrow}$ OCP^O photoconversion is much more probable than the OCP$^O \overset{hv}{\rightarrow}$ OCP^X photoconversion. Even if the observed nonlinearity is due to the full depletion of the OCP^O population caused by a strong irradiation pulse, the second light-triggered step must be characterized by an extremely low quantum yield. The reason is that regardless of the excitation

energy used, the band at 550 nm never significantly exceeds 2 mOD. Due to the negative feature at 545 nm, it is not easy to observe the OCP^R signature (expected at about 550 nm) when using low excitation energy density. At 12 mJ/cm^2, not much of the OCP^R form is observed, and for 3 mJ/cm^2 it is not detectable. We are convinced that this is due to the combination of the low quantum yields of both photoconversion steps and the existence of the aforementioned limiting processes. Therefore, we conclude that OCP has evolved to be insensitive to short light perturbations (there are more than one possible implementation of such effect). The practical consequence is that the possible number of "productive" photoconversion effects per time unit is finite and independent of the irradiation intensity.

Figures S14 and S15 show datasets acquired using a 488 nm excitation pulse with an energy density of approximately 50 mJ/cm^2 and 12 mJ/cm^2, respectively. The signals obtained with 488 nm excitation have comparable spectral shapes and intensities to ones obtained with 512 nm excitation. This excludes an effect of the OCP^O heterogeneity on the photoconversion mechanism[6, 7]; different OCP dark-adapted subpopulations photoconvert to OCP^R form in the same way.

Figure S16 demonstrates how a larger number of pulses leads to buildup of the OCP^R spectral signature in Plk/ECN observed after irradiation with continuous light. It shows that with an increasing number of excitation pulses, the peculiar negative spectral band observed after only one excitation pulse becomes more and more obscured by the well-known symmetrical OCP^R signature (with both negative and positive transient absorption contributions). Note that the negative 490 nm and positive 550 nm bands grow differently with the number of excitation pulses. This graph closes the gap between discrete and continuous light regimes, demonstrating the advantage of the two-pulse excitation approach.

Figures S8, S17 and S18 present data obtained for Syn/CAN using pump laser energy densities of ~50 mJ/cm^2, ~12 mJ/cm^2 and ~3 mJ/cm^2, respectively. As discussed in the main text, when using excitation pulses with the lowest energy density of ~3 mJ/cm^2, the second excitation pulse results in the same absorbance change as the first pulse, regardless of t_{delay}. This, however, does not hold for higher excitation energy densities. After ~12 mJ/cm^2 and ~50 mJ/cm^2, the second excitation pulse results in a higher absorbance change compared to the first pulse, and there is a small but consistent dependency of t_{delay}. The quantification of the amount of OCP^R product as a function of t_{delay} is visualized in Figure S20. Note also, the signal magnitude scales less than linearly with the excitation pulse energy density, indicating saturation and presence of some "limiting processes" similar to ones already discussed above for ECN-functionalized OCPs. Another effect present only for excitation energy densities above ~3 mJ/cm^2 is the faster decay of signals observed after the pair of excitation pulses, compared to signals observed after only one pulse (Figure S19).

2.2 Results obtained on modified proteins – tags, mutations

The rationale behind the experiments as well as the choice of mutants is described in the main text. Figures S21 and S22 show the results obtained from tagged Syn/ECN variants (N- and C-terminal His-tags, respectively). The behavior of these samples differs from tag-free Syn/ECN (Figure S10): after one excitation pulse one can observe a spectral signature that resembles OCP^R (positive band close to 550 nm). Nevertheless, it is evident that the photoactivation mechanism still requires two photons because this band decays completely within 1 s, clearly indicating that it is not associated with an OCP^R form. The OCP^R signature lives only long enough to be considered as a bona fide OCP^R form, when a properly delayed second excitation pulse has been applied.

Figures S23 and S24 show the results obtained for the L37V mutants (Syn/ECN/L37V, Syn/CAN/L37V, respectively). These mutant variants display significantly lower signal amplitude than WT. Nevertheless, the mutation preserves the type of photoconversion mechanism, indicating that the steric interaction between the β2-keto group of the carotenoid and the L37 residue is not a factor that decides whether the photoconversion mechanism is two or single photon. This conclusion is further confirmed by the results obtained for the L37A mutant (Syn/CAN) shown in Figure S25. Crystal structure analysis of the L37V and L37A mutants complexed with ECN/CAN, respectively, show no structural changes compared to wildtype (data not shown). Figure S26 shows the mutation site in the protein structure and the dependence of the OCP^R product yield of t_{delay}.

Figure S27 shows the results obtained for the Plk/ECN/R27L mutant. The R27L mutant protein is known to be monomeric due to disruption of the R27-D19$^{\#}$ interaction at the dimerization interface[8] ($^{\#}$ indicates a residue in the other monomer in the OCP dimer). Compared to WT (Figure S11), the signal is about two times higher (note that an accurate comparison of the yield is not possible because the signal depends also on the accuracy of the pump-probe spatial overlap and the sample absorbance which is not always exactly identical). The R27L mutation does not result in an opening of the single-photon channel, demonstrating that the two-photon effect is not rooted directly in the dimeric state of OCP. The dimeric WT OCP needs to dissociate into monomers upon photoexcitation to enable the CTD-NTD domain separation required for OCP^R formation[9, 10]. As this dissociation process requires energy, it is expected that the OCP^R yield is reduced to some extent in a dimeric versus monomeric protein. Nevertheless, since R27L mutant requires two properly timed excitation pulses in order to photoconvert to the OCP^R form, it is clear that the two-photon mechanism must be rooted in something else than the requirement of dimer dissociation.

Figure S28 shows results obtained for the Plk/CAN/R27L mutant. It has the same photoconversion characteristics and thus mechanism as WT (Figure S7). The main difference is an increase of the signal magnitude, analogously to the Plk/ECN/R27L sample.

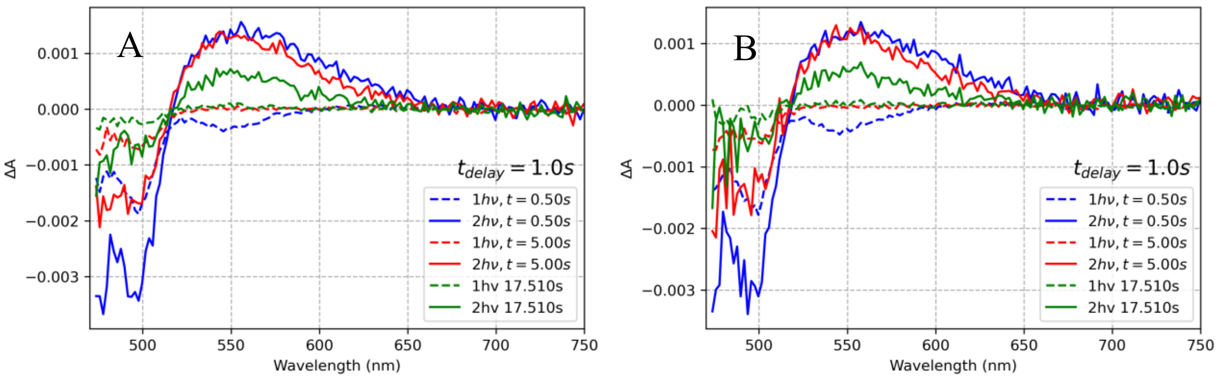

Figure S6. Comparison of two different probing intensities used in a two-pulse experiment on *Planktothrix* OCP functionalized with ECN, with His-tag removed (two different cuvette fillings, therefore the concentration may slightly differ). Excitation at 512 nm, energy density about 50 mJ/cm^2. A) Standard probe used in all experiments, B) the same probe attenuated by the factor of two.

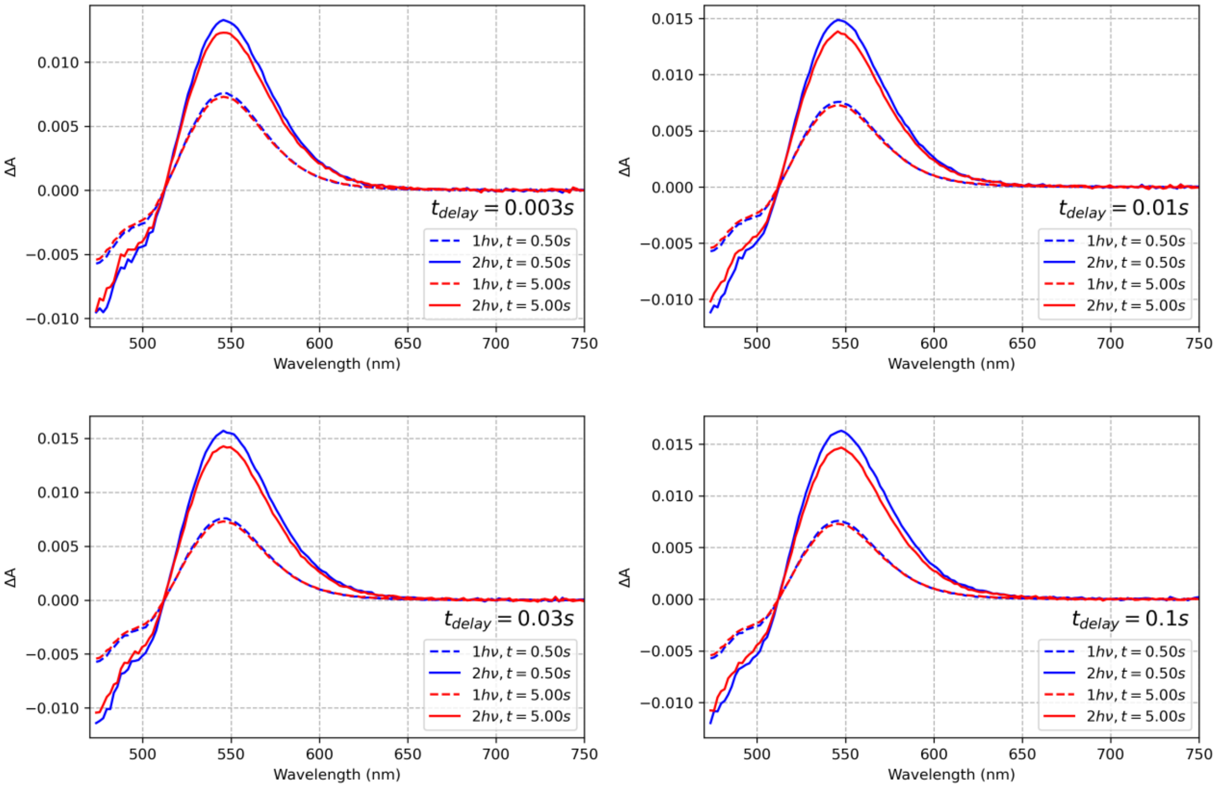

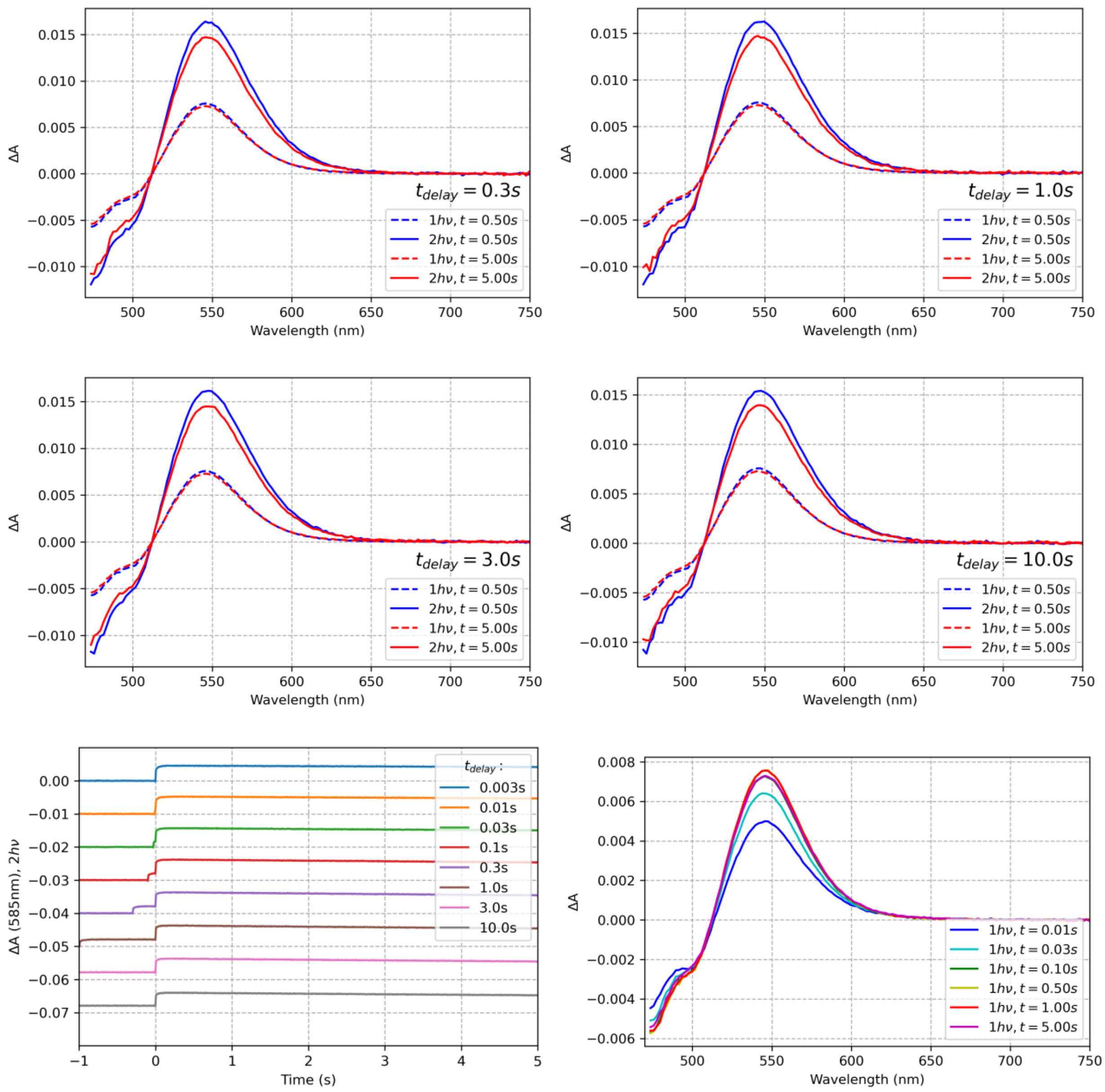

Figure S7. Two pulse experiment results obtained for OCP from *Planktothrix* functionalized with CAN, with the His-tag removed. Excitation at 512 nm, energy density about 50 mJ/cm^2.

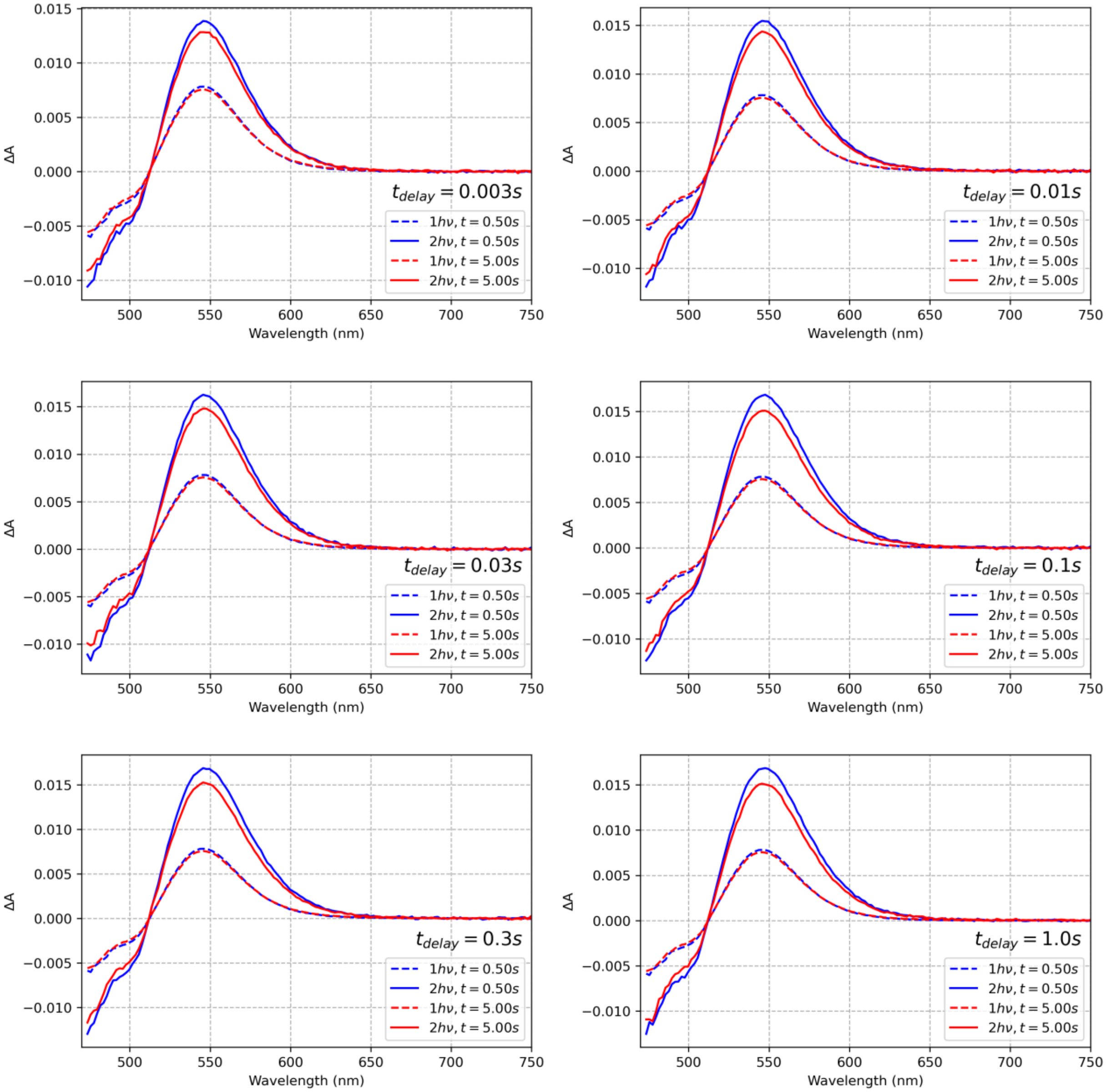

ΔA
Wavelength (nm)
$t_{delay} = 0.003s$
$t_{delay} = 0.01s$
$t_{delay} = 0.03s$
$t_{delay} = 0.1s$
$t_{delay} = 0.3s$
$t_{delay} = 1.0s$
$1h\nu, t = 0.50s$
$2h\nu, t = 0.50s$
$1h\nu, t = 5.00s$
$2h\nu, t = 5.00s$

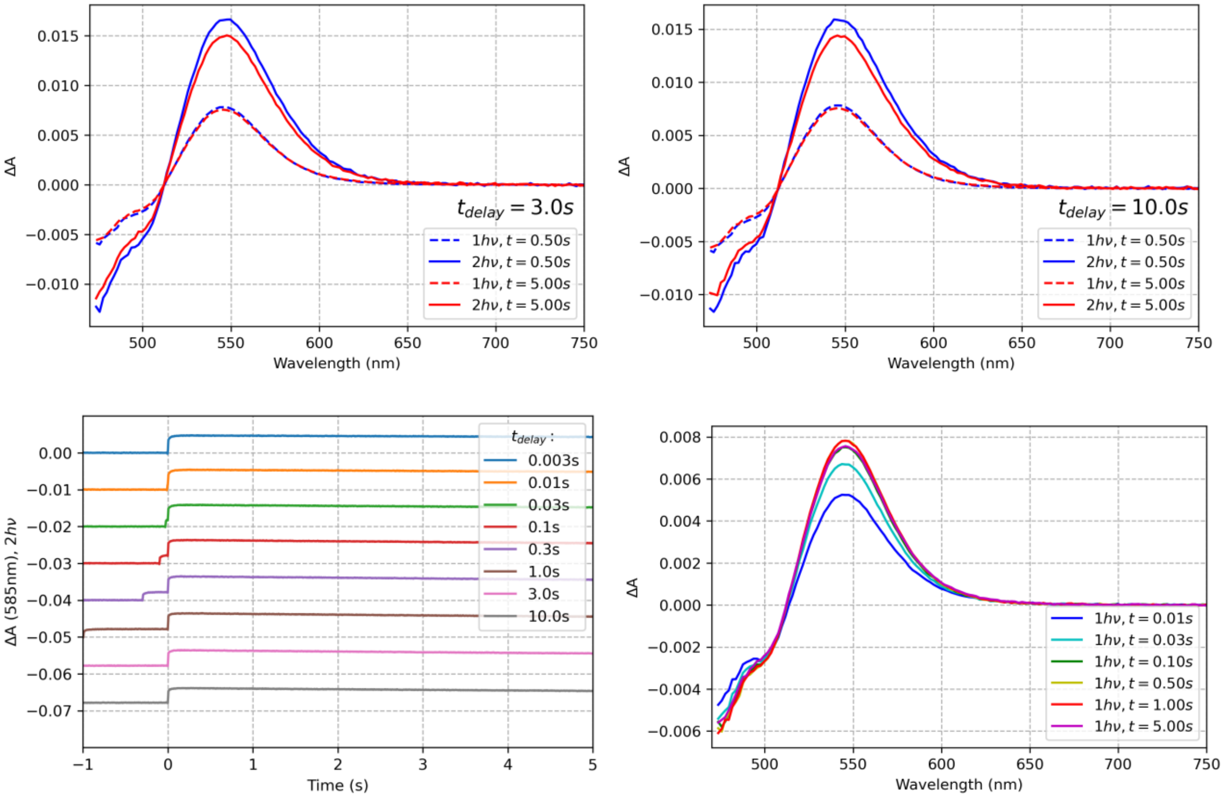

Figure S8. Two pulse experiment results obtained for OCP from *Synechosystis* functionalized with CAN, with His-tag removed. Excitation at 512 nm, energy density about 50 mJ/cm^2.

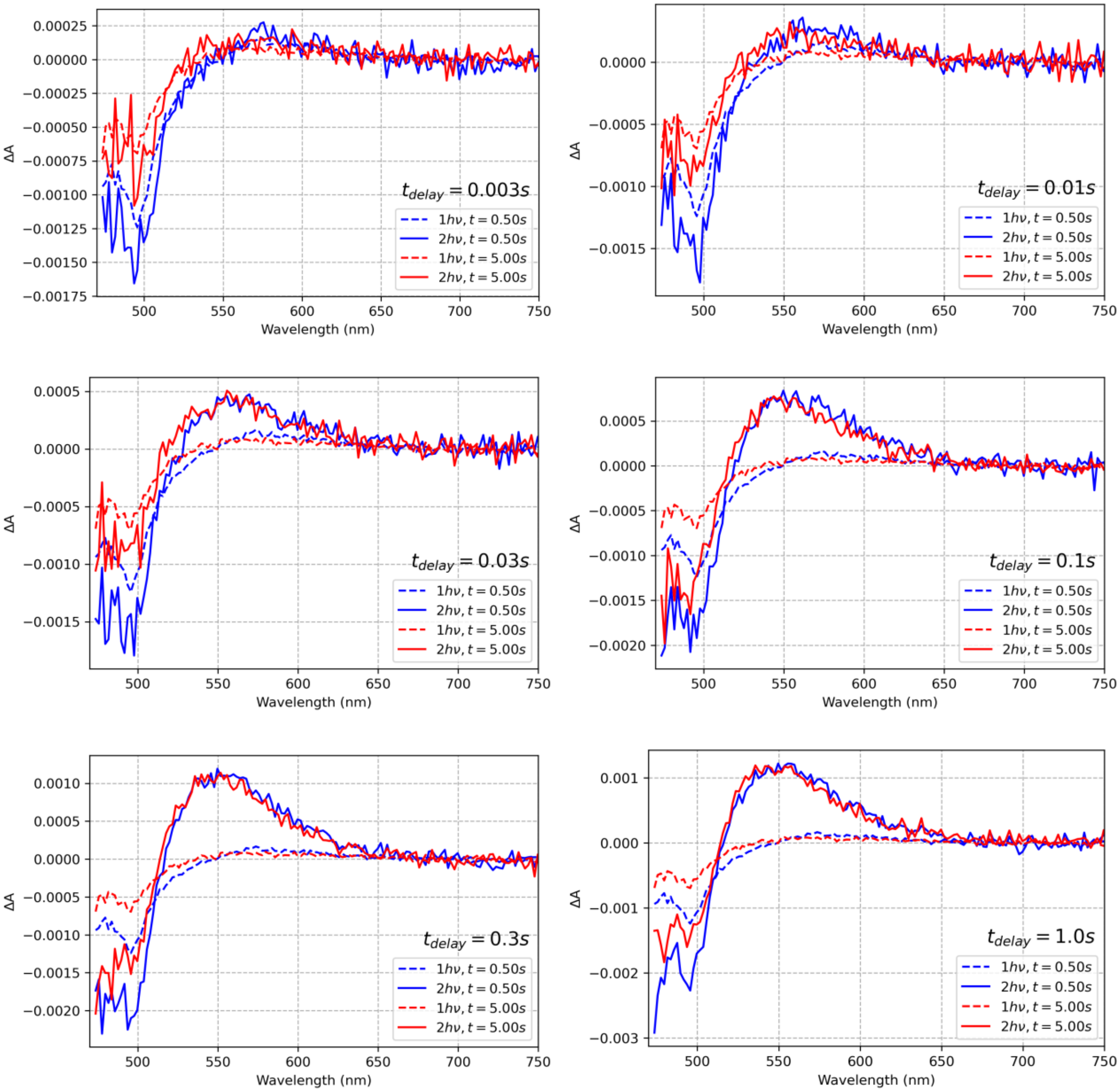

ΔA
Wavelength (nm)
$t_{delay} = 0.003s$
$t_{delay} = 0.01s$
$t_{delay} = 0.03s$
$t_{delay} = 0.1s$
$t_{delay} = 0.3s$
$t_{delay} = 1.0s$
$1h\nu, t = 0.50s$
$2h\nu, t = 0.50s$
$1h\nu, t = 5.00s$
$2h\nu, t = 5.00s$

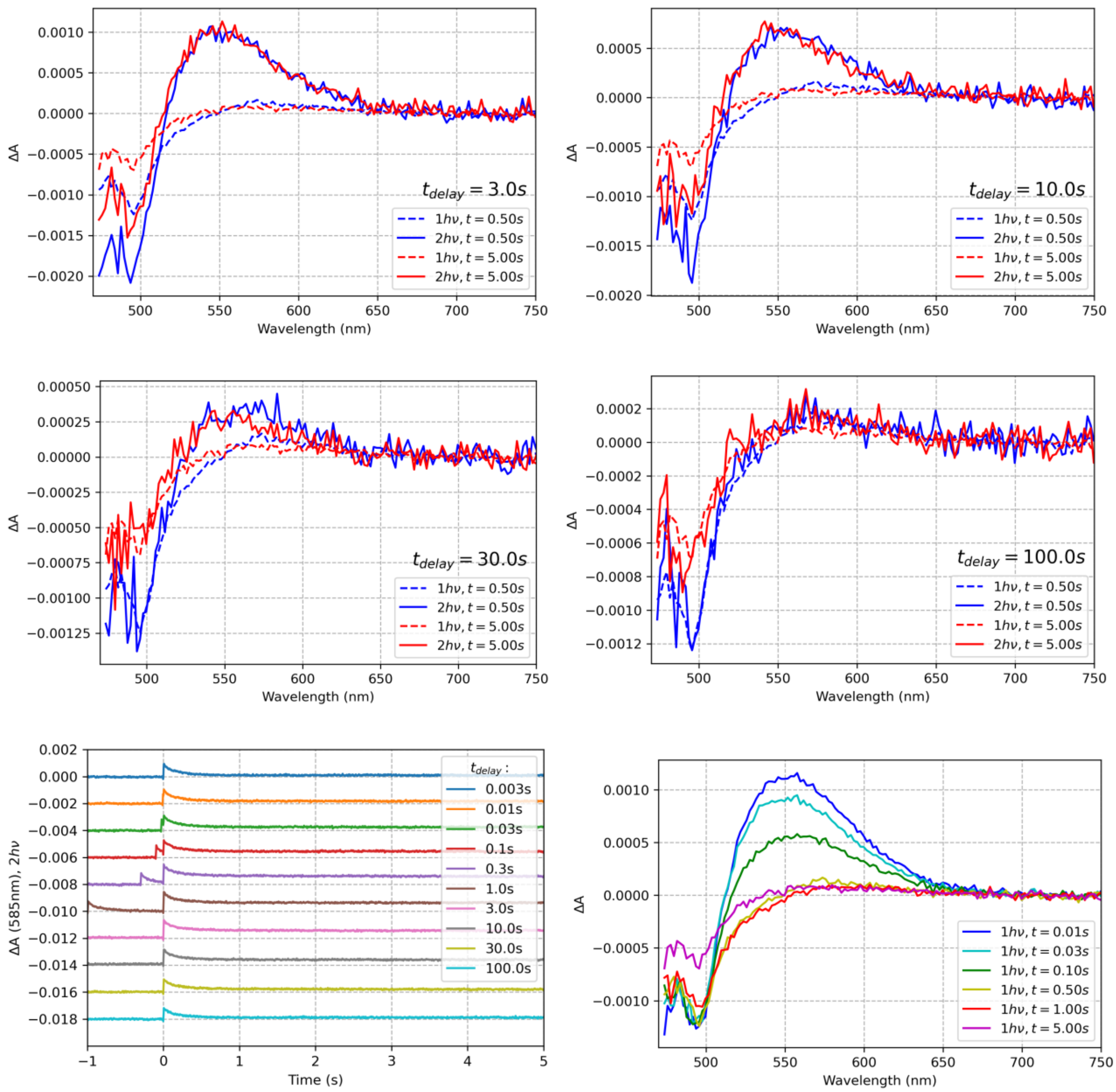

Figure S9. Two pulse experiment results obained for OCP from *Synechosystis* functionalized with hECN, with His-tag removed. Excitation at 512 nm, energy density about 50 mJ/cm^2.

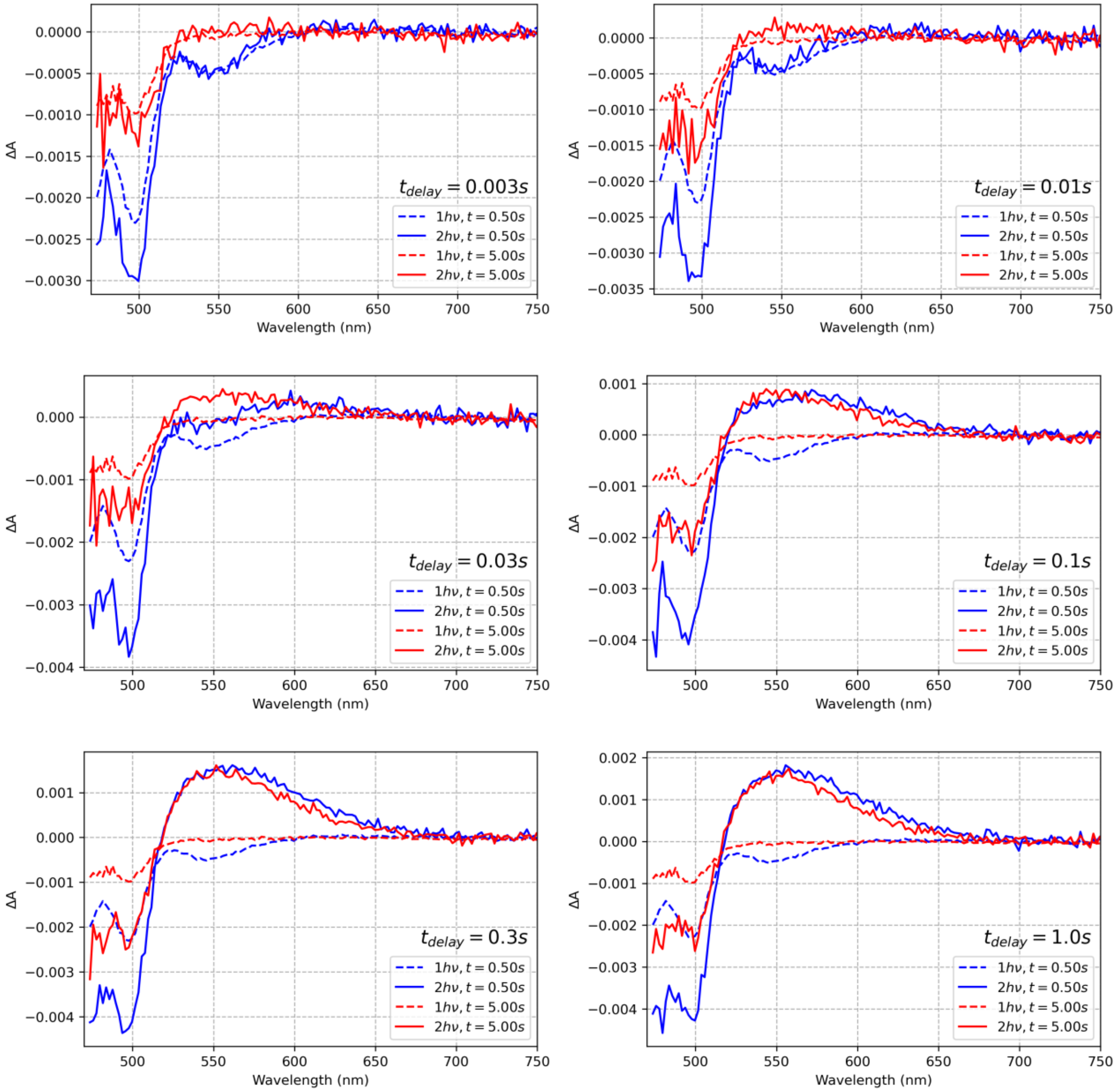
ΔA
Wavelength (nm)
$t_{delay} = 0.003s$
$t_{delay} = 0.01s$
$t_{delay} = 0.03s$
$t_{delay} = 0.1s$
$t_{delay} = 0.3s$
$t_{delay} = 1.0s$
$1h\nu, t = 0.50s$
$2h\nu, t = 0.50s$
$1h\nu, t = 5.00s$
$2h\nu, t = 5.00s$

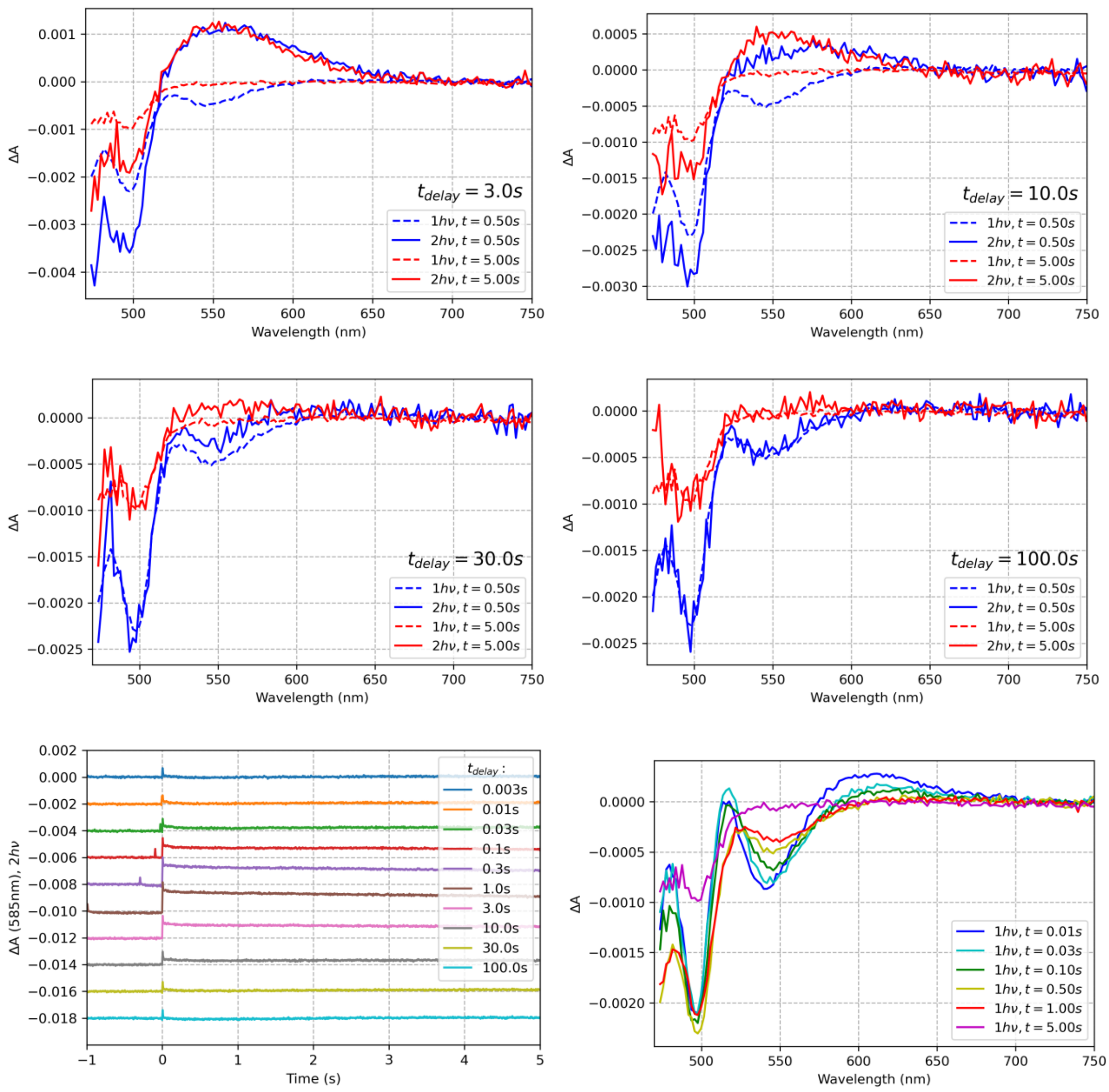

Figure S10. Two pulse experiment results obtained for OCP from *Synechocystis* functionalized with ECN, with His-tag removed. Excitation at 512 nm, energy density about 50 mJ/cm^2.

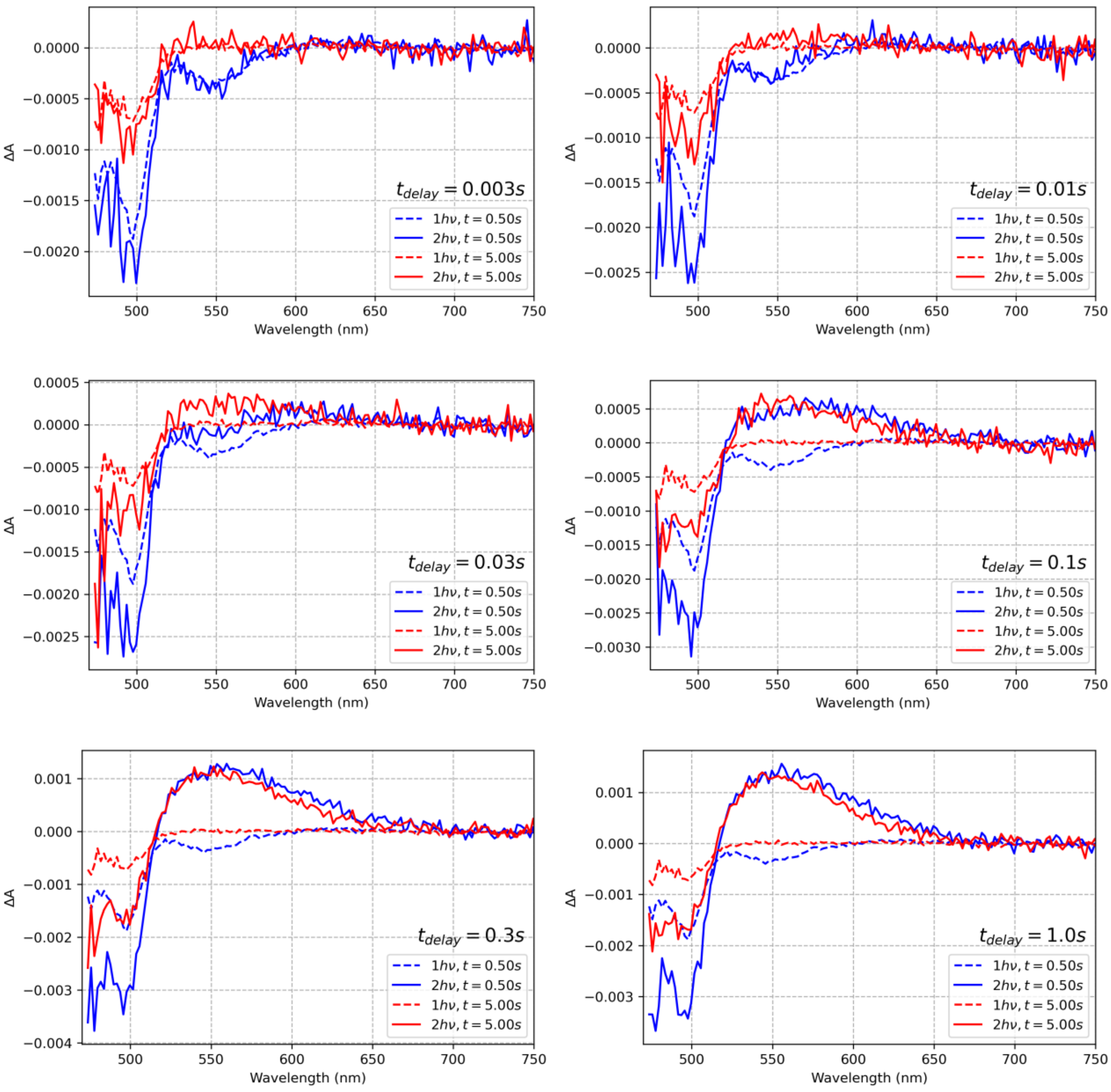

$t_{delay} = 0.003s$
$t_{delay} = 0.01s$
$t_{delay} = 0.03s$
$t_{delay} = 0.1s$
$t_{delay} = 0.3s$
$t_{delay} = 1.0s$
$1h\nu, t = 0.50s$
$2h\nu, t = 0.50s$
$1h\nu, t = 5.00s$
$2h\nu, t = 5.00s$
ΔA
Wavelength (nm)

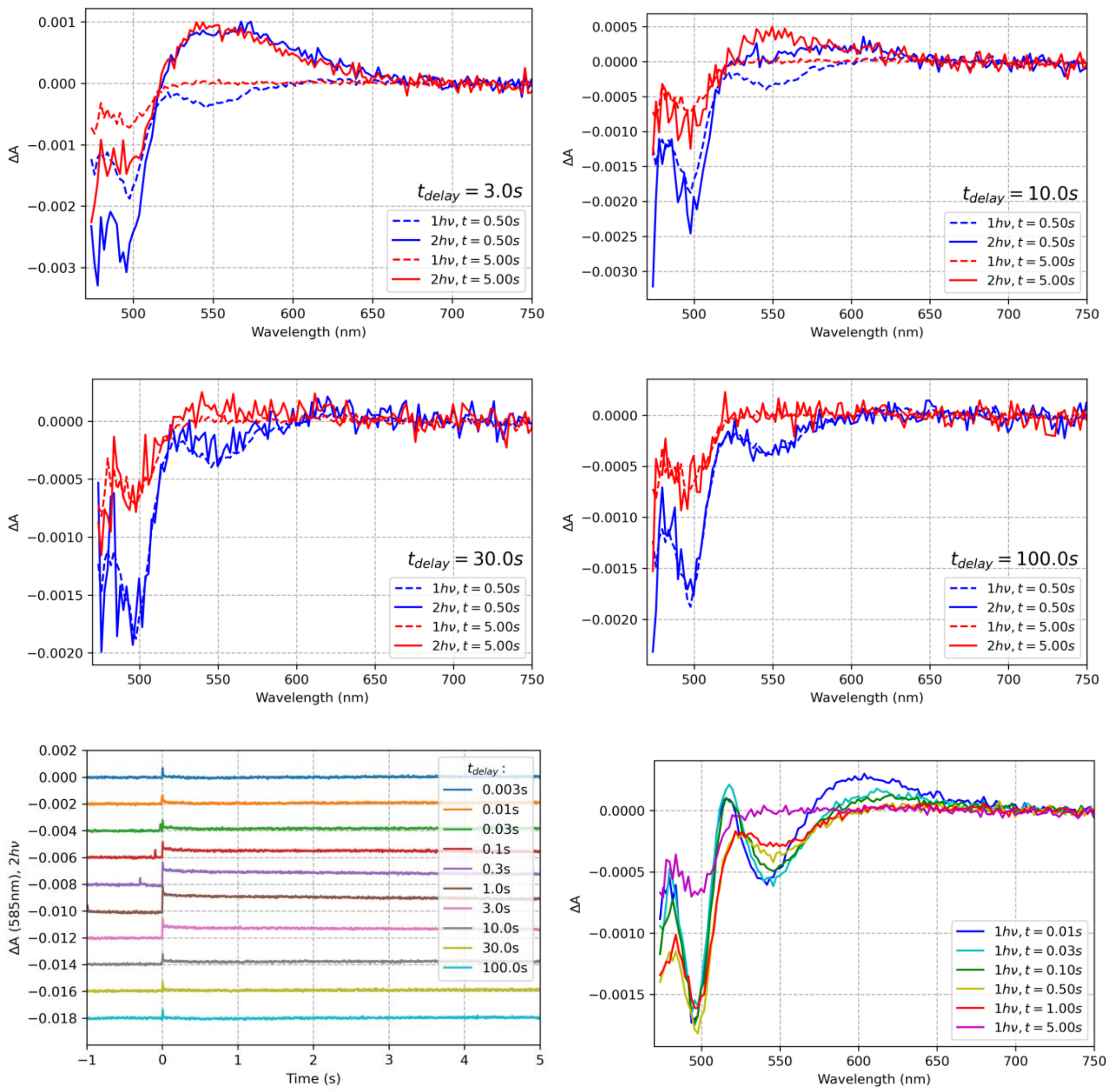

Figure S11. Two pulse experiment results obtained for OCP from *Planktothrix* functionalized with ECN, with His-tag removed. Excitation at 512 nm, energy density about 50 mJ/cm^2.

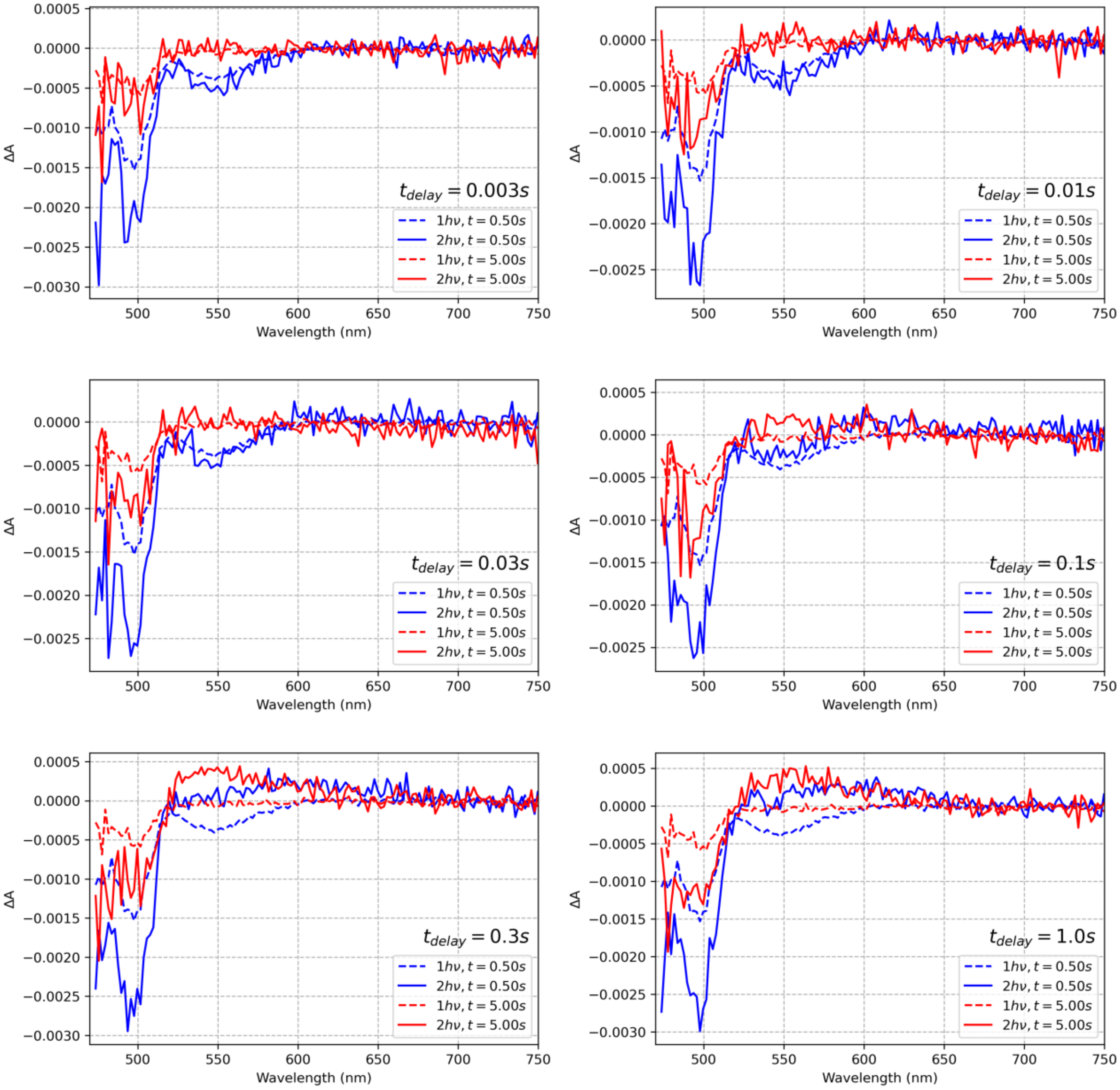
ΔA
Wavelength (nm)
$t_{delay} = 0.003s$
$t_{delay} = 0.01s$
$t_{delay} = 0.03s$
$t_{delay} = 0.1s$
$t_{delay} = 0.3s$
$t_{delay} = 1.0s$
$1h\nu, t = 0.50s$
$2h\nu, t = 0.50s$
$1h\nu, t = 5.00s$
$2h\nu, t = 5.00s$

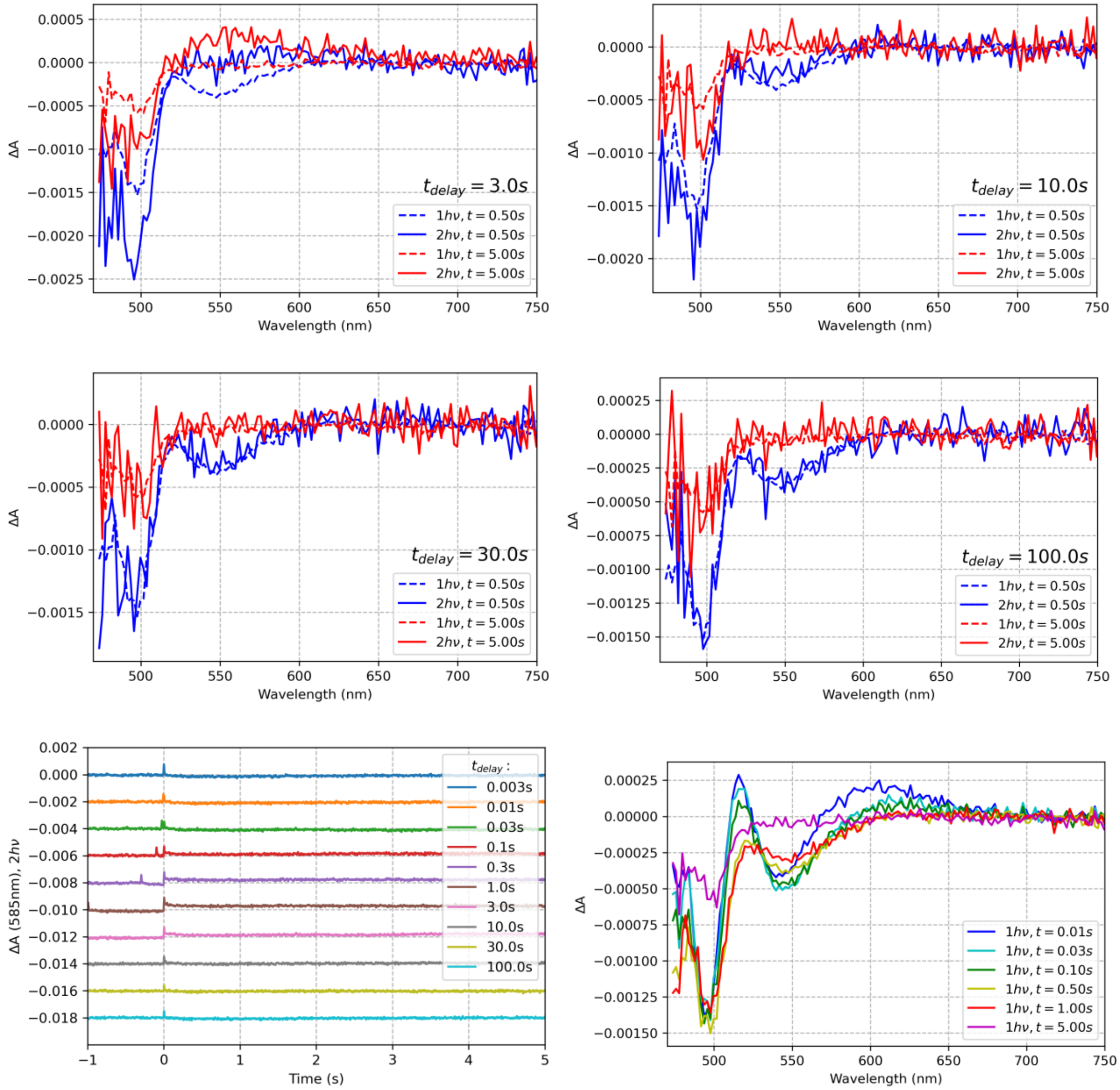

Figure S12. Two pulse experiment results obtained for OCP from *Planktothrix* functionalized with ECN, with His-tag removed. Excitation at 512 nm, energy density about 12 mJ/cm^2.

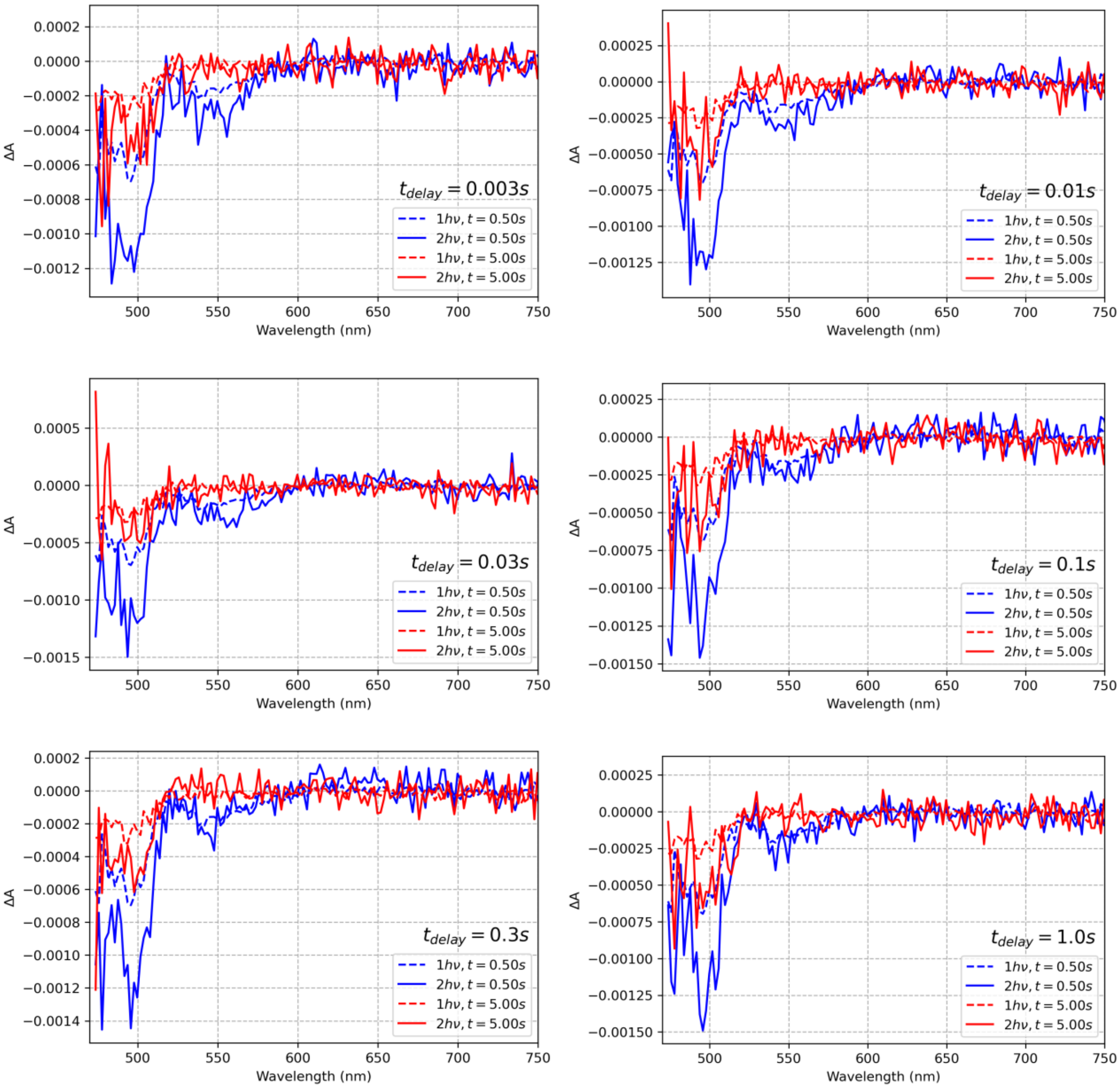

$t_{delay} = 0.003s$
$t_{delay} = 0.01s$
$t_{delay} = 0.03s$
$t_{delay} = 0.1s$
$t_{delay} = 0.3s$
$t_{delay} = 1.0s$
$1h\nu, t = 0.50s$
$2h\nu, t = 0.50s$
$1h\nu, t = 5.00s$
$2h\nu, t = 5.00s$
ΔA
Wavelength (nm)

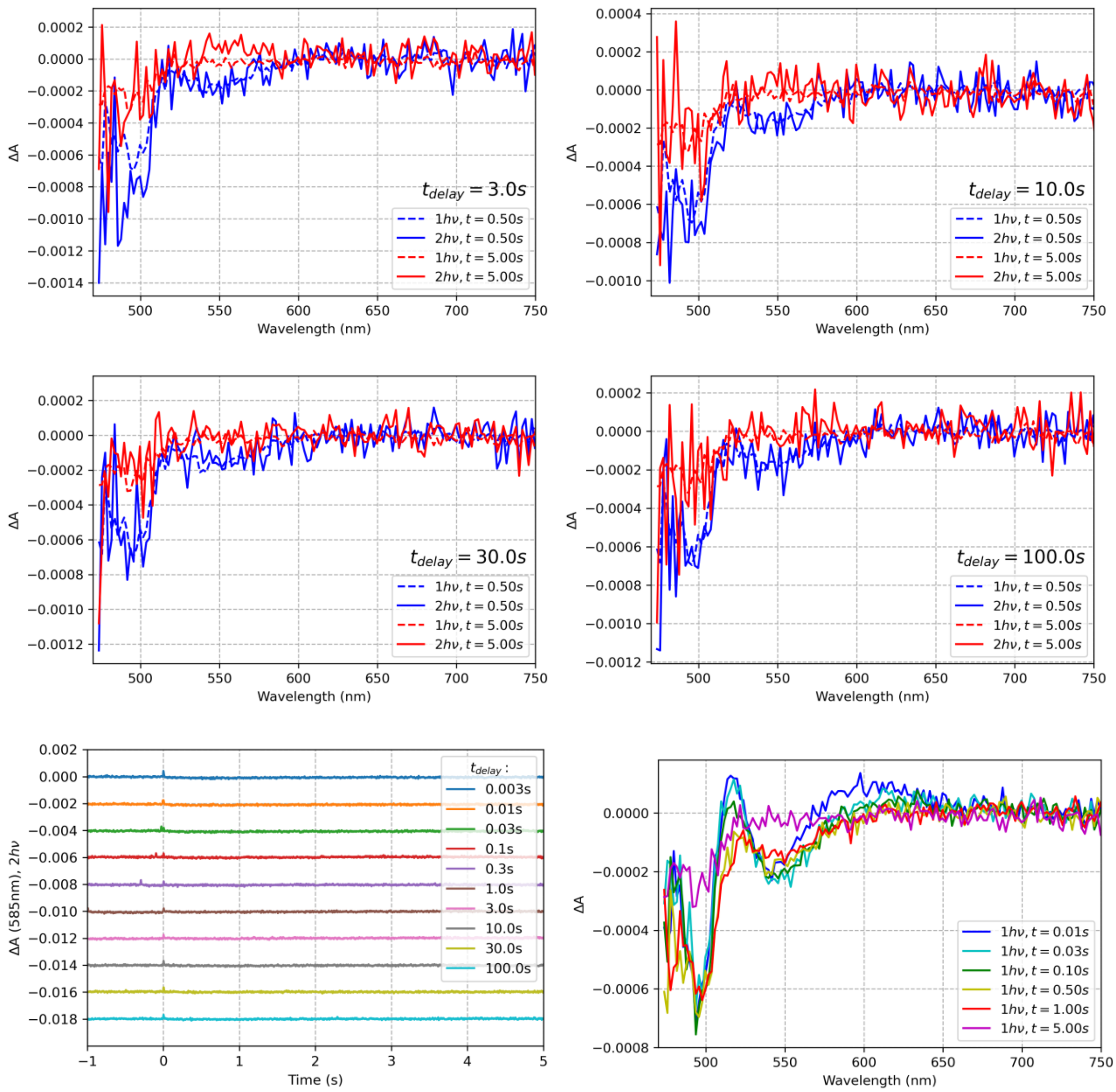

Figure S13. Two pulse experiment results obtained for OCP from *Planktothrix* functionalized with ECN, with His-tag removed. Excitation at 512 nm, energy density about 3 mJ/cm^2.

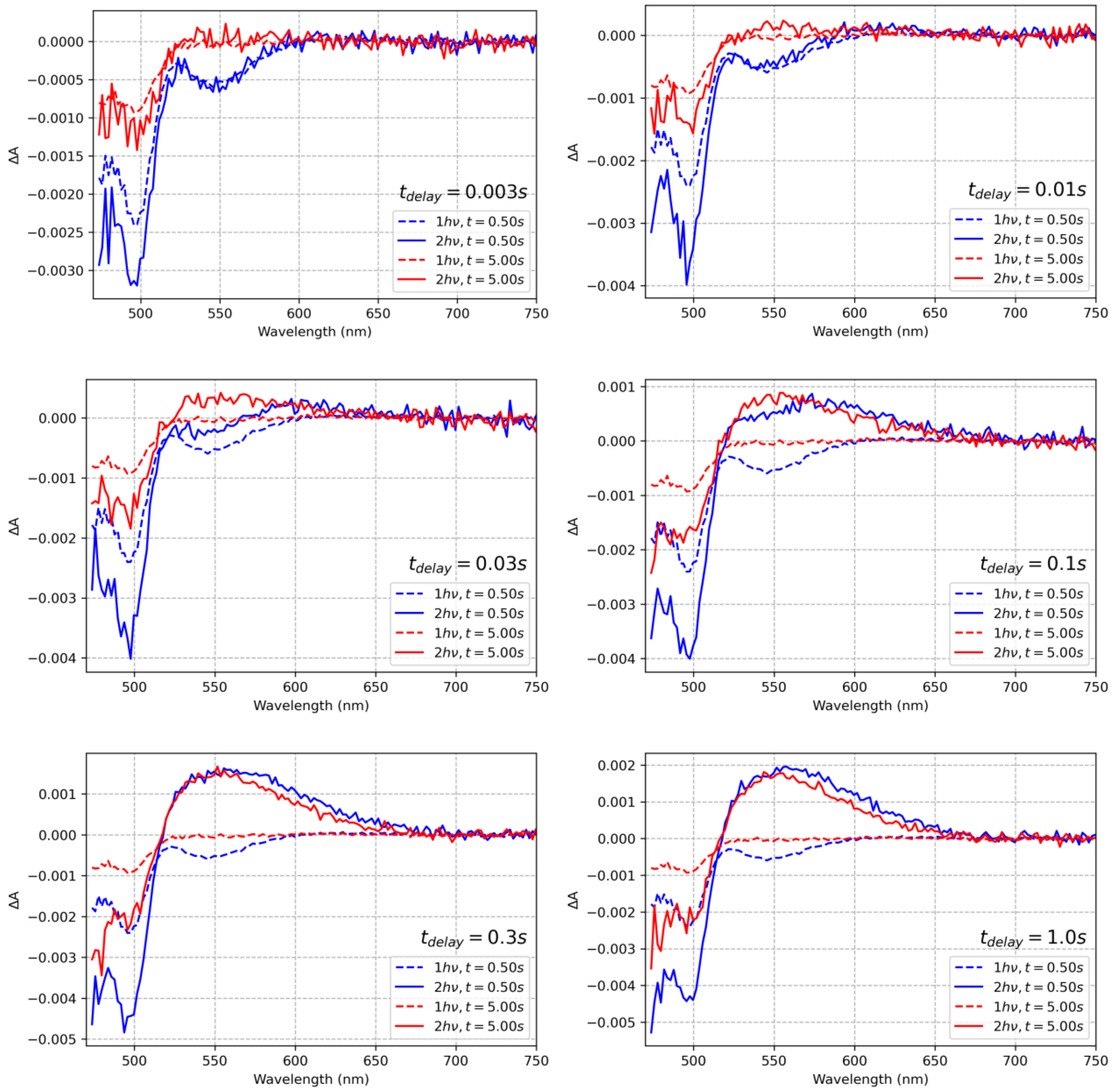

$t_{delay} = 0.003s$
$t_{delay} = 0.01s$
$t_{delay} = 0.03s$
$t_{delay} = 0.1s$
$t_{delay} = 0.3s$
$t_{delay} = 1.0s$
$1h\nu, t = 0.50s$
$2h\nu, t = 0.50s$
$1h\nu, t = 5.00s$
$2h\nu, t = 5.00s$
ΔA
Wavelength (nm)

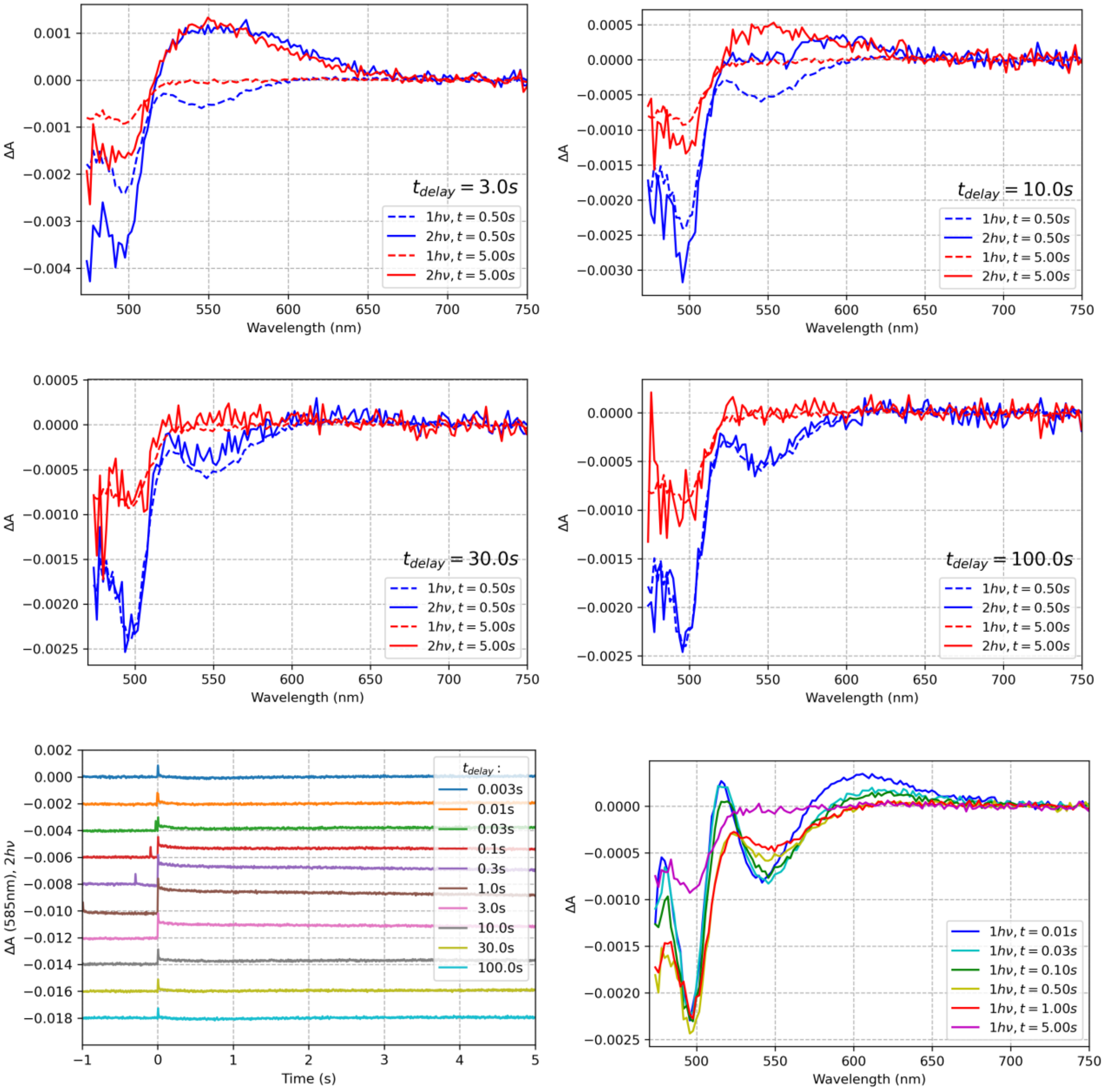

Figure S14. Two pulse experiment results obtained for OCP from *Planktothrix* functionalized with ECN, with His-tag removed. Excitation at 488 nm, energy density set very roughly to 50 mJ/cm^2.

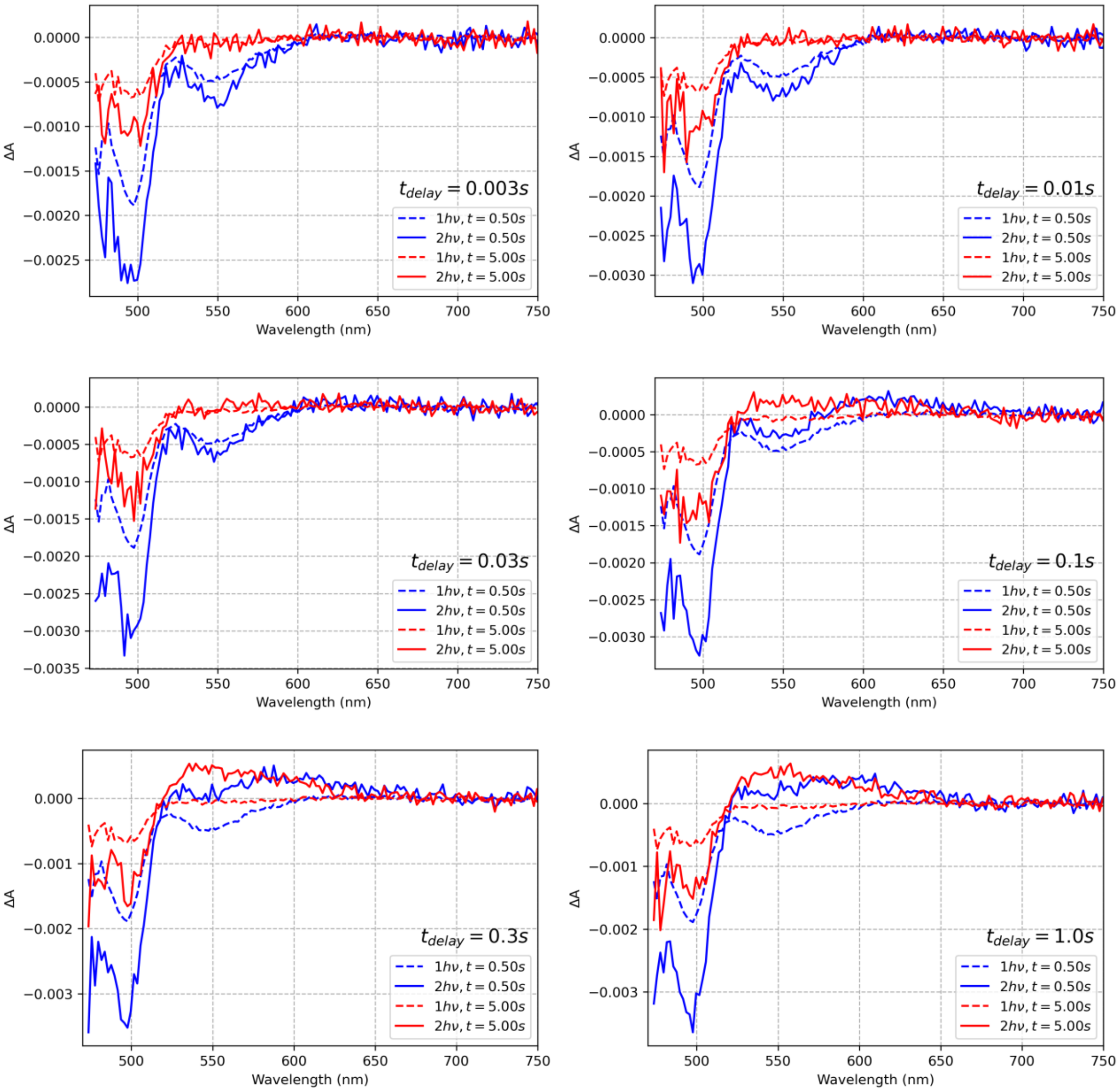
ΔA
Wavelength (nm)
$t_{delay} = 0.003s$
$t_{delay} = 0.01s$
$t_{delay} = 0.03s$
$t_{delay} = 0.1s$
$t_{delay} = 0.3s$
$t_{delay} = 1.0s$
$1h\nu, t = 0.50s$
$2h\nu, t = 0.50s$
$1h\nu, t = 5.00s$
$2h\nu, t = 5.00s$

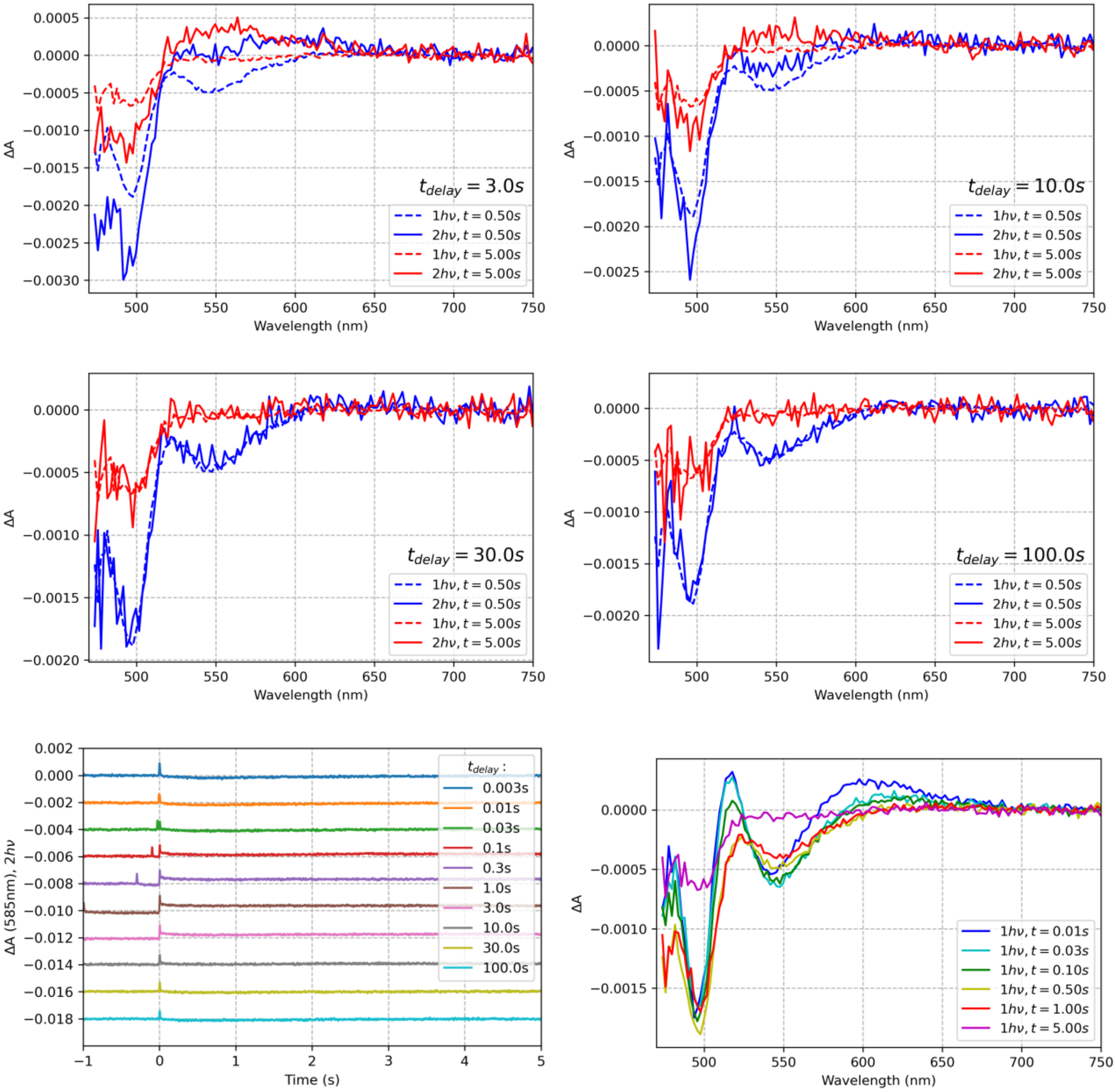

Figure S15. Two pulse experiment results obtained for OCP from *Planktothrix* functionalized with ECN, with His-tag removed. Excitation at 488 nm, energy density set very roughly 12 mJ/cm^2.

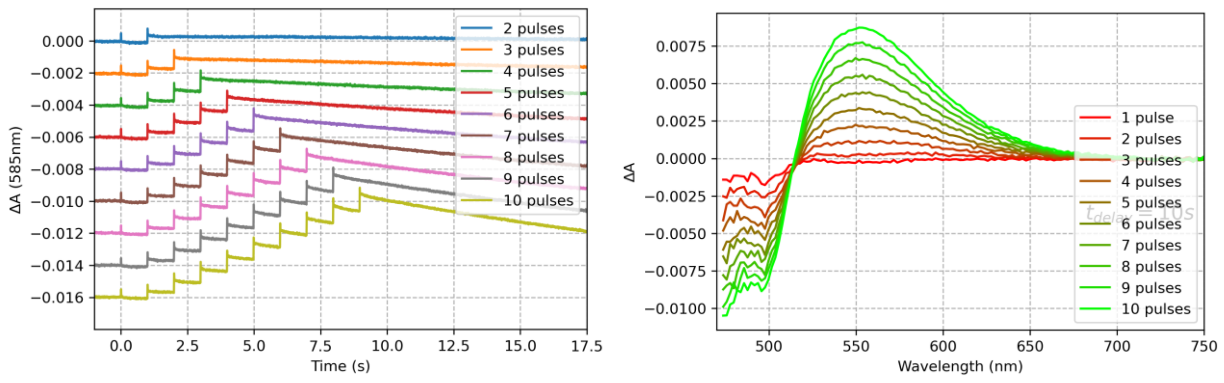

Figure S16. Photoconversion triggered by pulse trains comprising different number of pulses in the train. Experiment results obtained for OCP from *Planktothrix* functionalized with ECN, with His-tag removed. Excitation at 512 nm, pulse energy density about 12 mJ/cm^2.

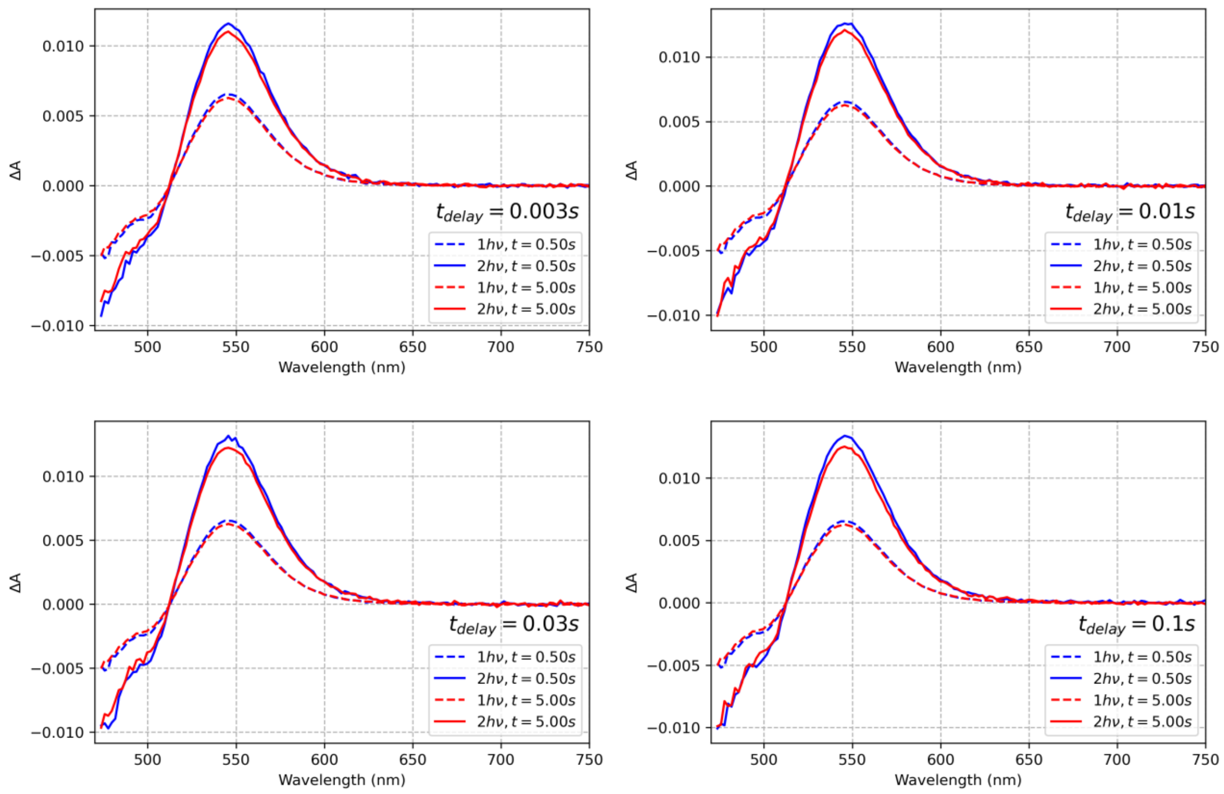

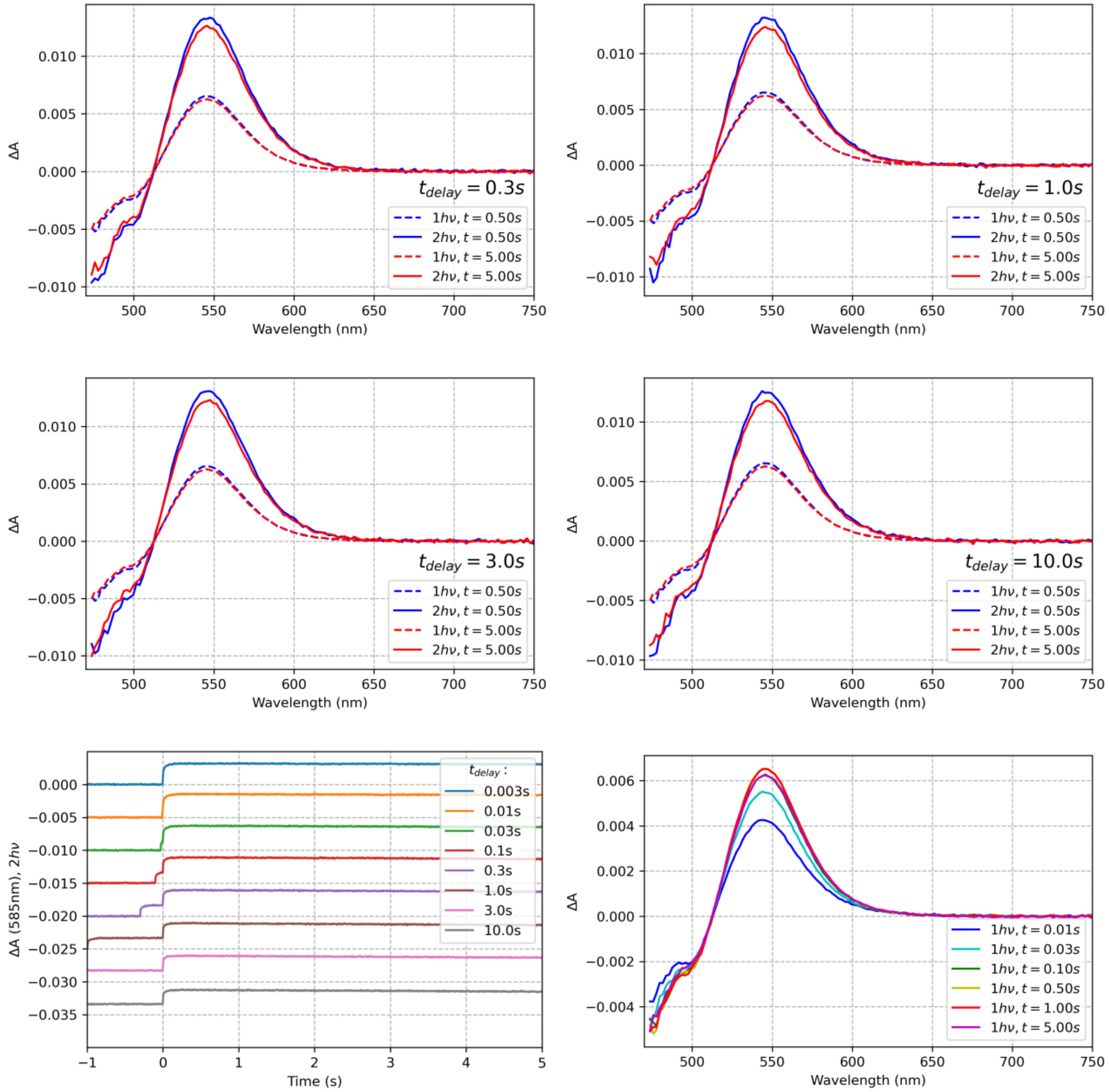

Figure S17. Two pulse experiment results obtained for OCP from *Synechocystis* functionalized with CAN, with His-tag removed. Excitation at 512 nm, energy density about 12 mJ/cm^2.

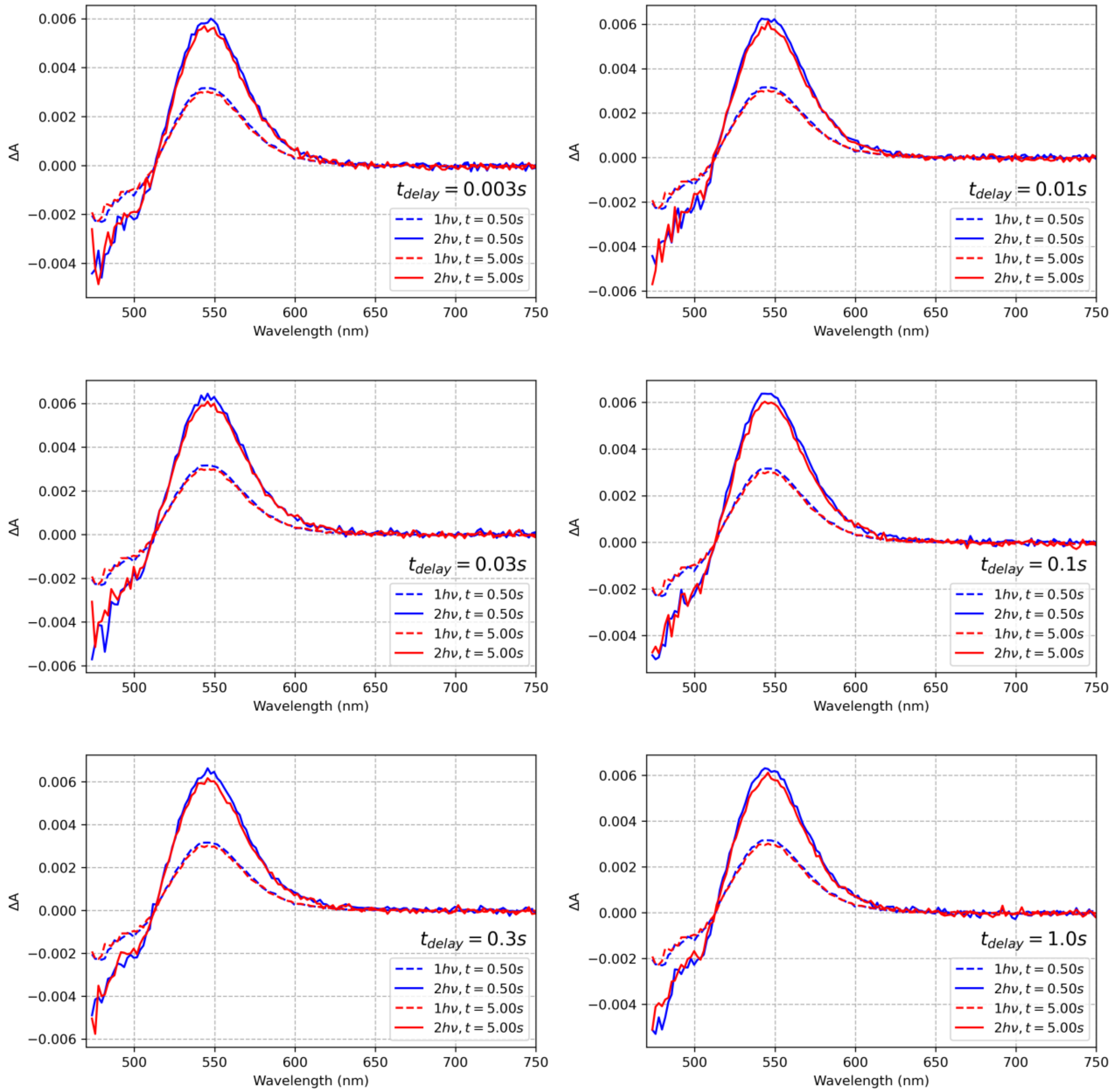

$t_{delay} = 0.003s$
$t_{delay} = 0.01s$
$t_{delay} = 0.03s$
$t_{delay} = 0.1s$
$t_{delay} = 0.3s$
$t_{delay} = 1.0s$
$1h\nu, t = 0.50s$
$2h\nu, t = 0.50s$
$1h\nu, t = 5.00s$
$2h\nu, t = 5.00s$
ΔA
Wavelength (nm)

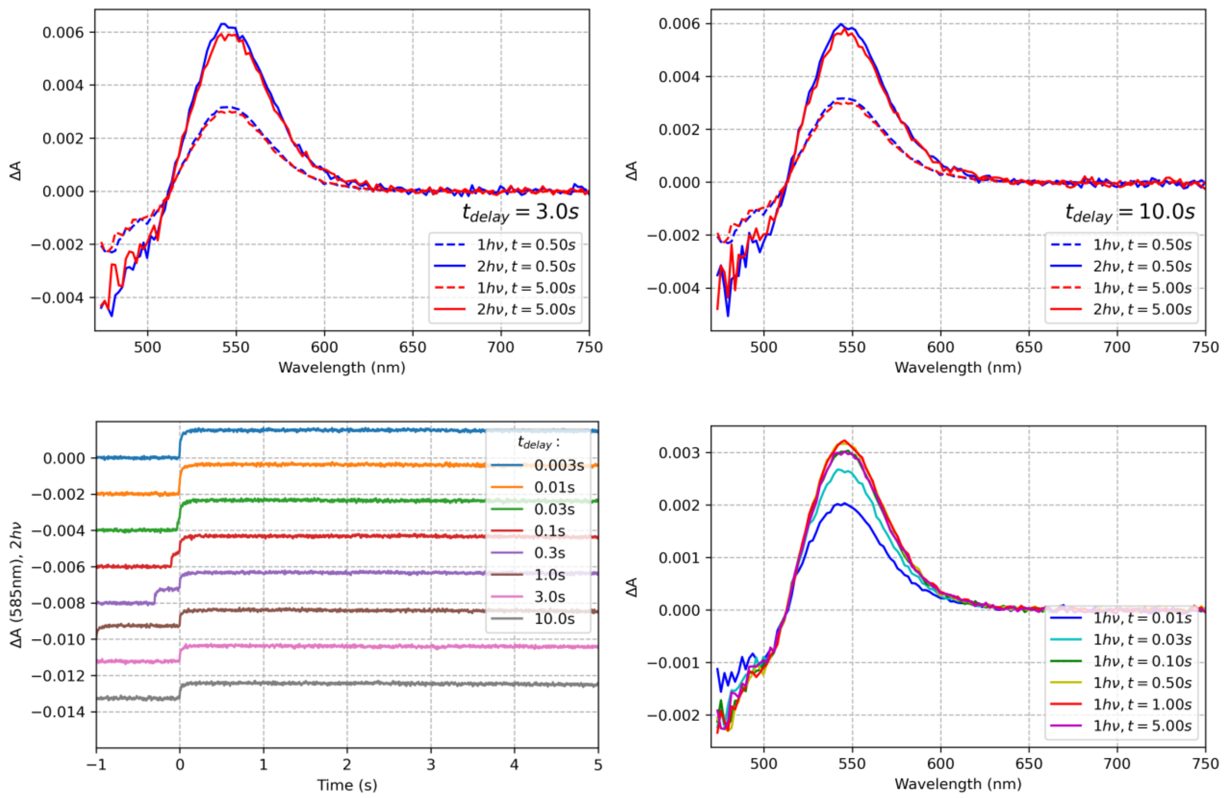

Figure S18. Two pulse experiment results obtained for OCP from *Synechocystis* functionalized with CAN, with His-tag removed. Excitation at 512 nm, energy density about 3 mJ/cm^2.

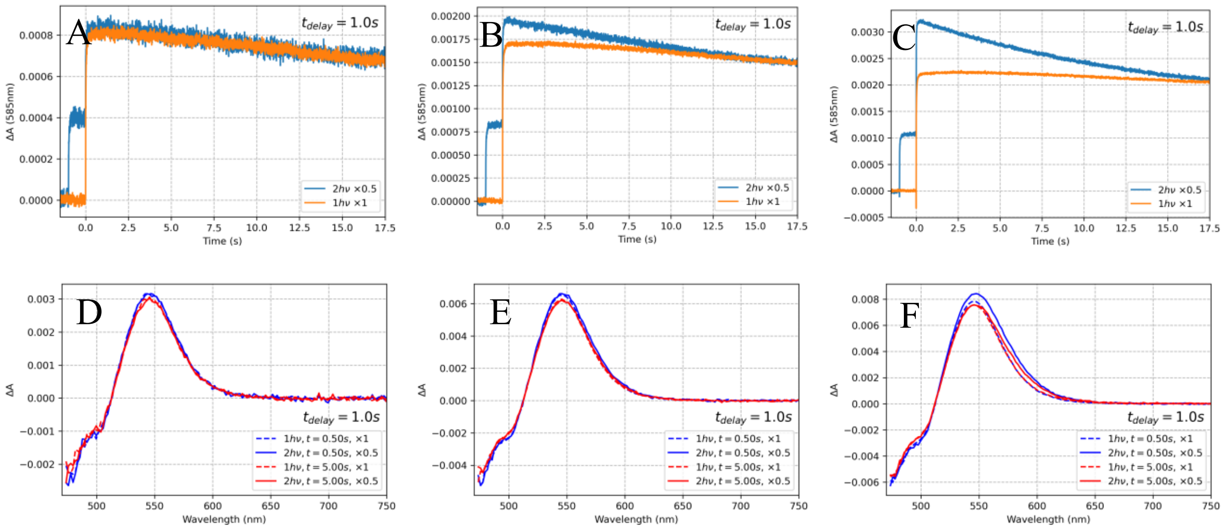

Figure S19. Two pulse experiment results obtained for OCP from *Synechocystis* functionalized with CAN, with His-tag removed. Excitation at 512 nm, energy density about 3 mJ/cm^2 (A, D), 12 mJ/cm^2 (B, E), 50 mJ/cm^2 (C, F). Spectra and kinetics are divided by the respective number of pulses used, t_{delay} is 1 s.

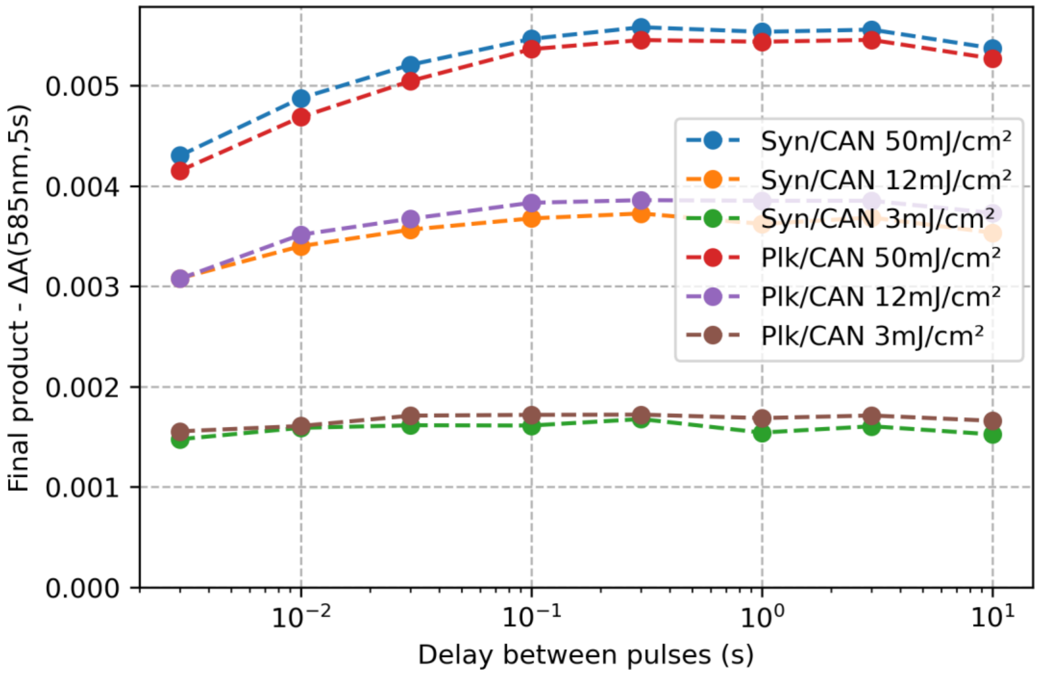

Figure S20. The change in absorption at 585 nm (5 s after the second pulse), representing the yield of OCP^R, plotted against t_{delay} for CAN-functionalized OCPs and various excitation energy densities (512 nm).

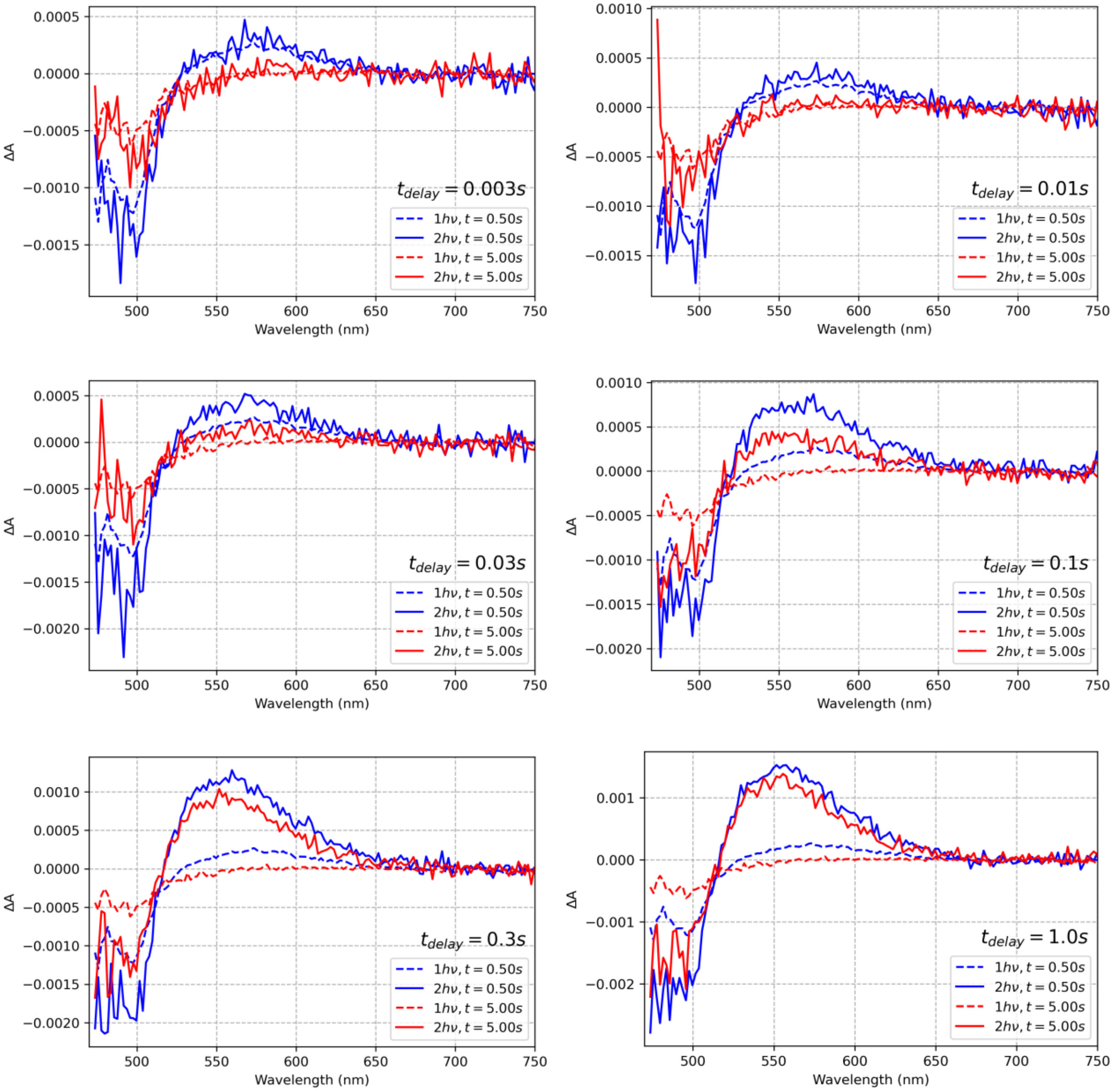

ΔA
Wavelength (nm)
$t_{delay} = 0.003s$
$t_{delay} = 0.01s$
$t_{delay} = 0.03s$
$t_{delay} = 0.1s$
$t_{delay} = 0.3s$
$t_{delay} = 1.0s$
$1h\nu, t = 0.50s$
$2h\nu, t = 0.50s$
$1h\nu, t = 5.00s$
$2h\nu, t = 5.00s$

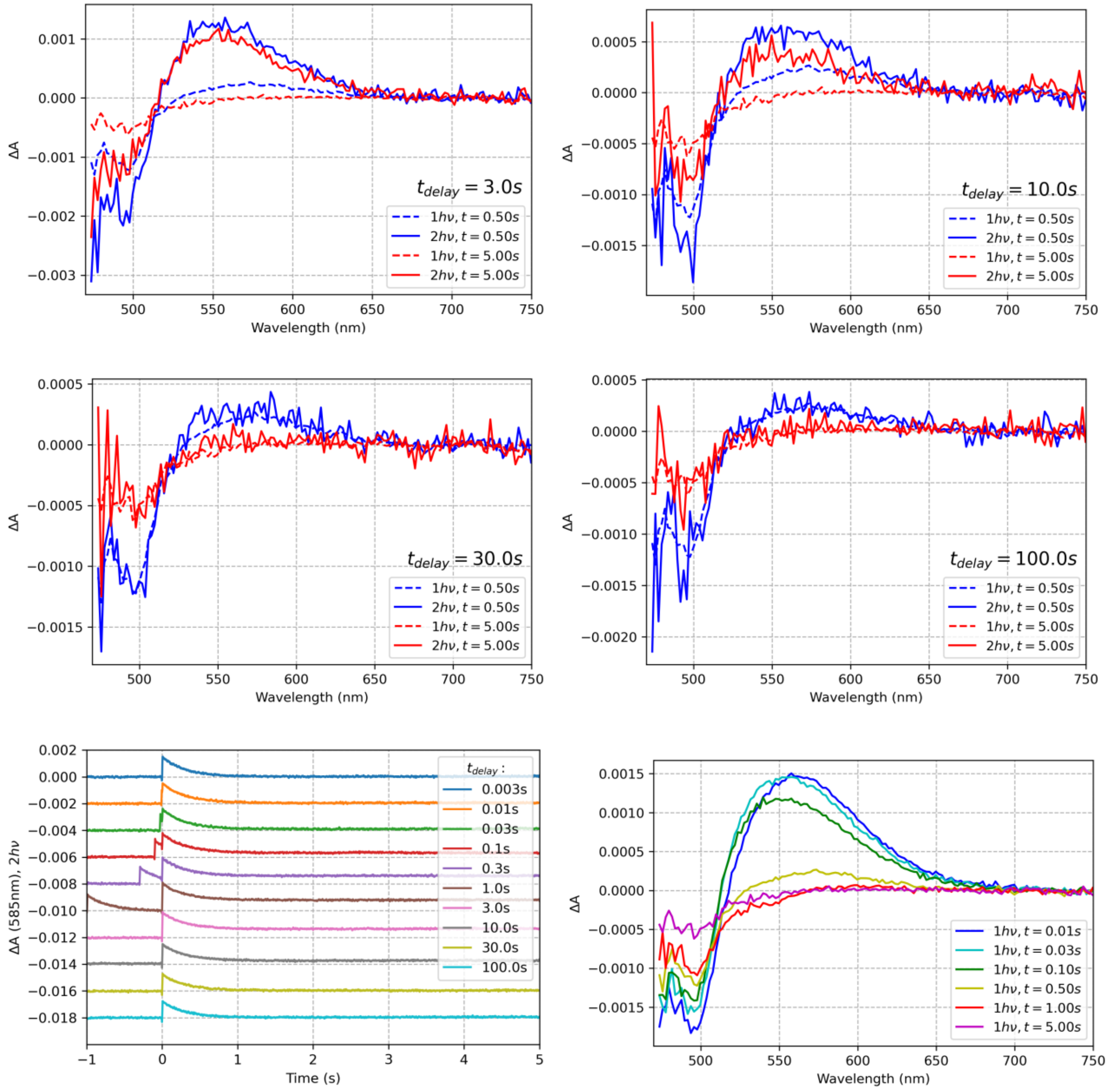

Figure S21. Two pulse experiment results obtained for OCP from *Synechocystis* functionalized with ECN with a His-tag at the N-terminus. Excitation at 512 nm, energy density about 50 mJ/cm^2.

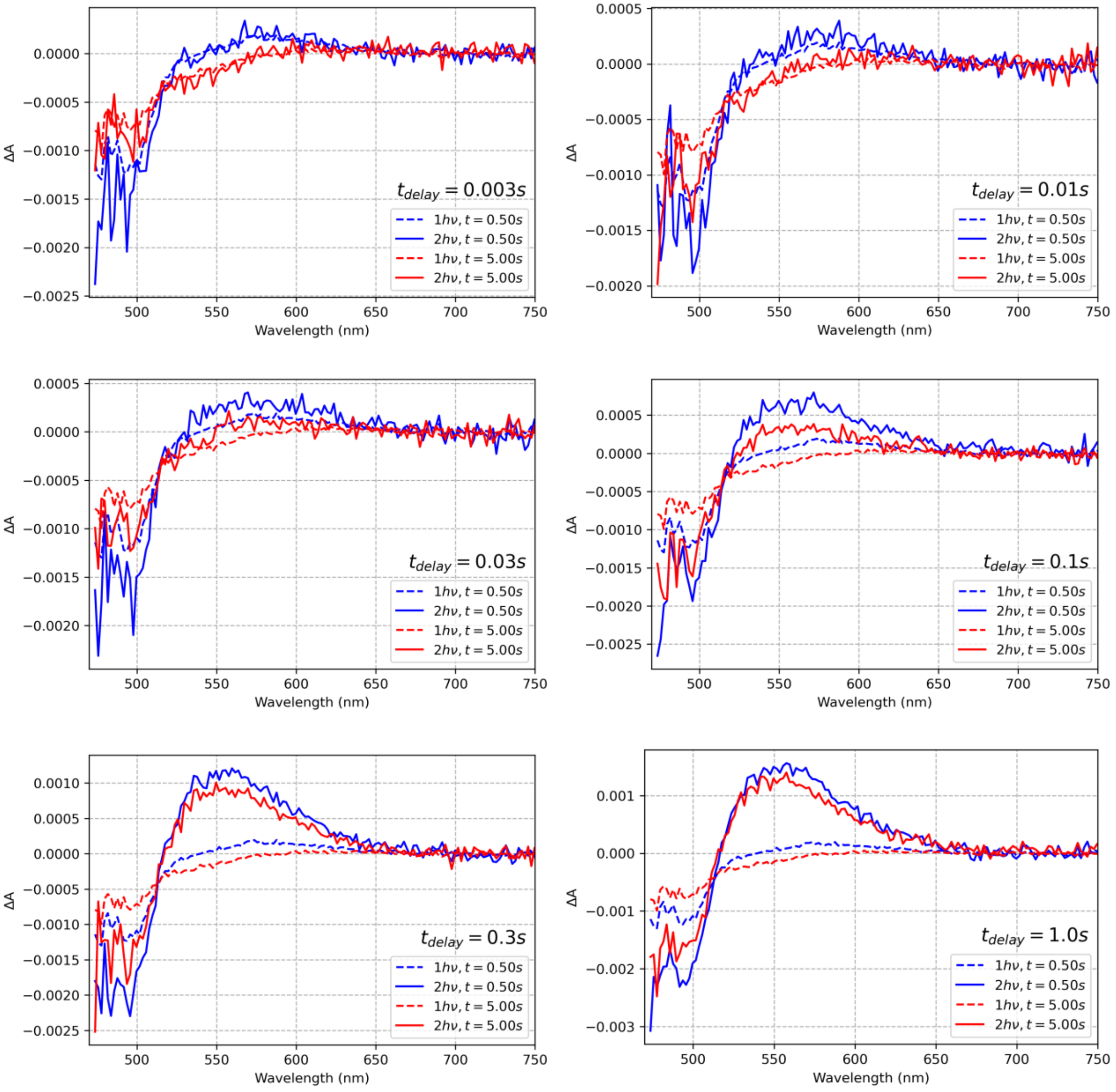
$t_{delay} = 0.003s$
$t_{delay} = 0.01s$
$t_{delay} = 0.03s$
$t_{delay} = 0.1s$
$t_{delay} = 0.3s$
$t_{delay} = 1.0s$
$1h\nu, t = 0.50s$
$2h\nu, t = 0.50s$
$1h\nu, t = 5.00s$
$2h\nu, t = 5.00s$
ΔA
Wavelength (nm)

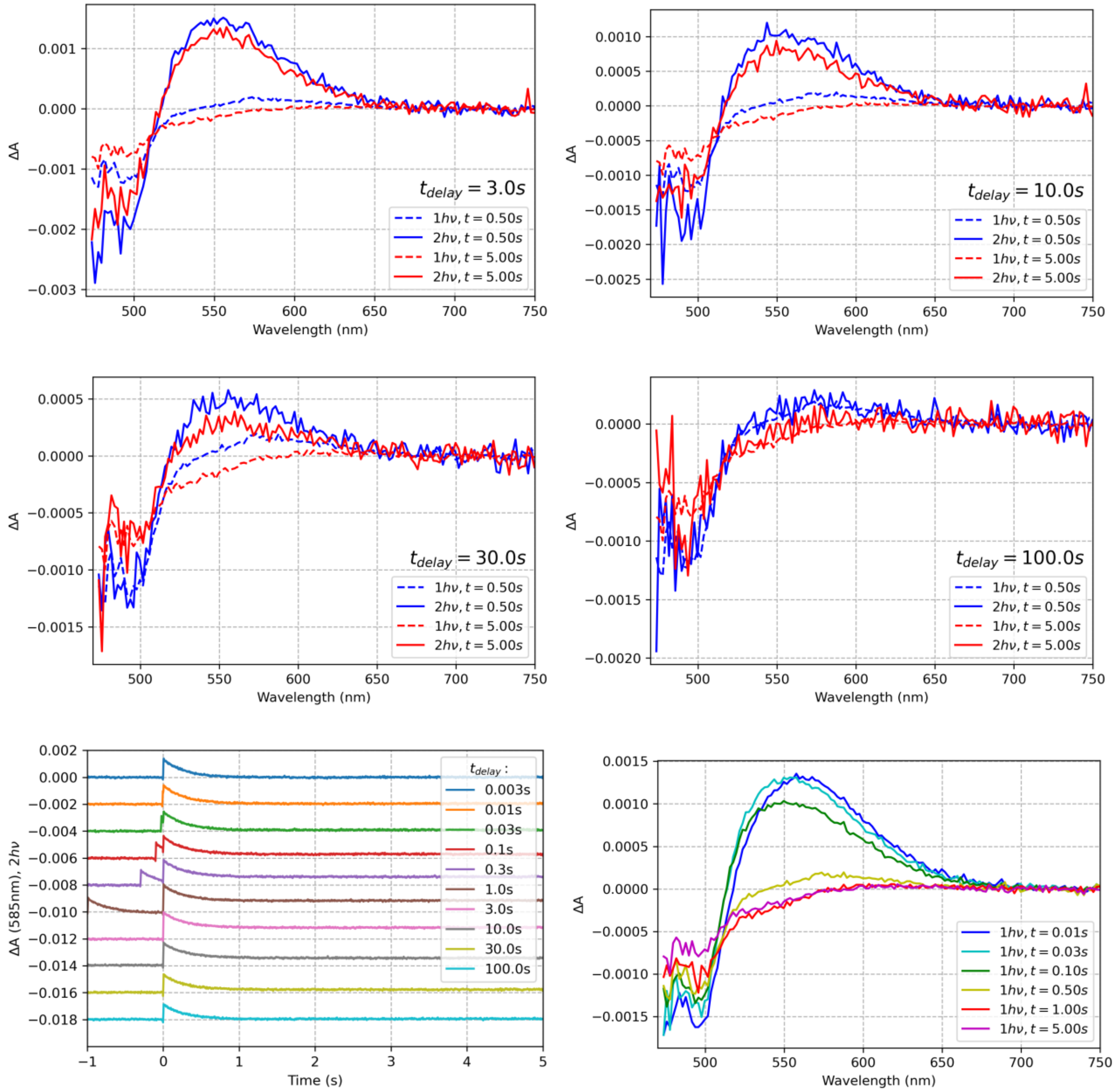

Figure S22. Two pulse experiment results obtained for OCP from *Synechocystis* functionalized with ECN with a His-tag at the C-terminus. Excitation at 512 nm, energy density about 50 mJ/cm^2.

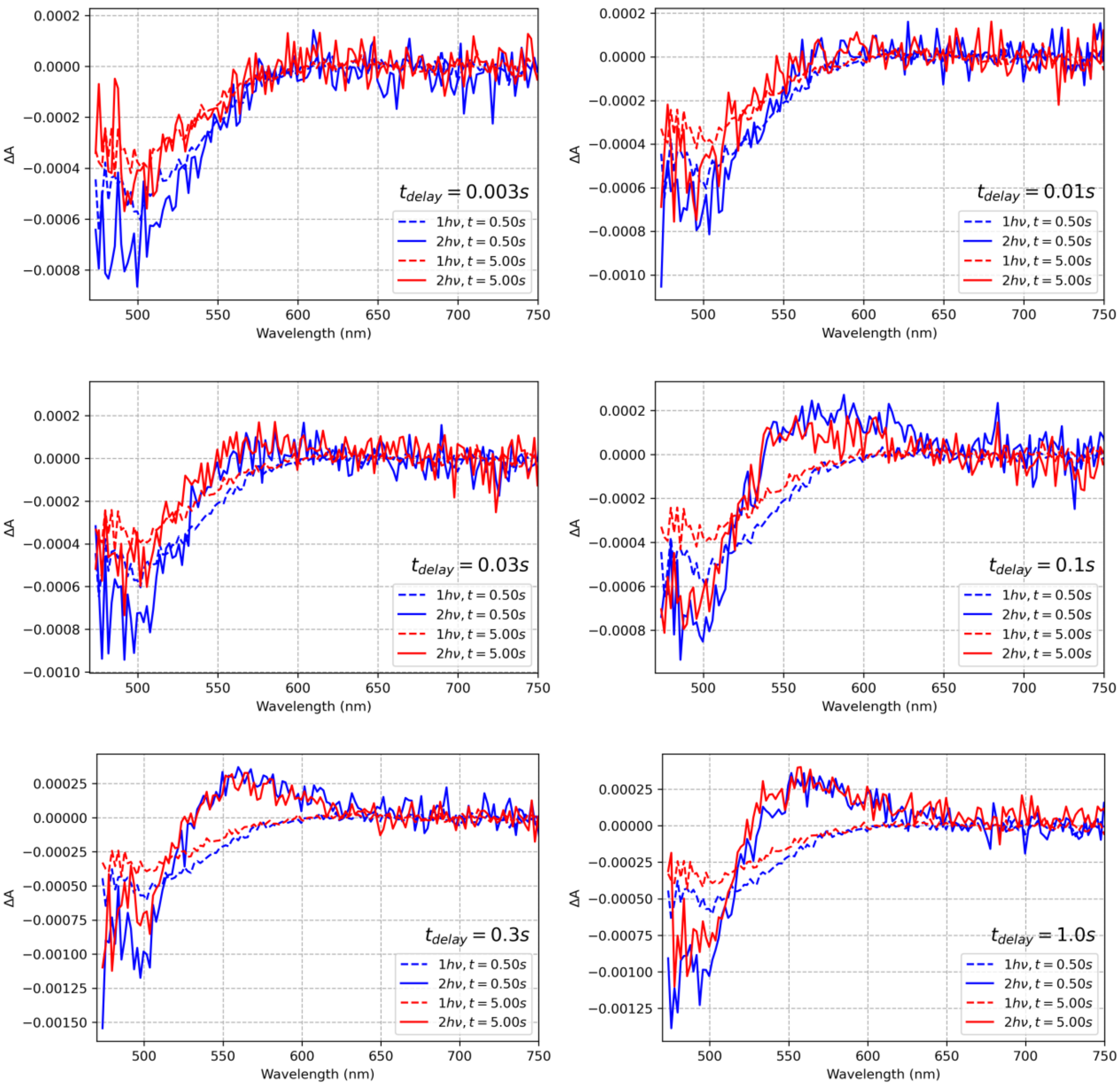

ΔA
Wavelength (nm)
$t_{delay} = 0.003s$
$t_{delay} = 0.01s$
$t_{delay} = 0.03s$
$t_{delay} = 0.1s$
$t_{delay} = 0.3s$
$t_{delay} = 1.0s$
$1h\nu, t = 0.50s$
$2h\nu, t = 0.50s$
$1h\nu, t = 5.00s$
$2h\nu, t = 5.00s$

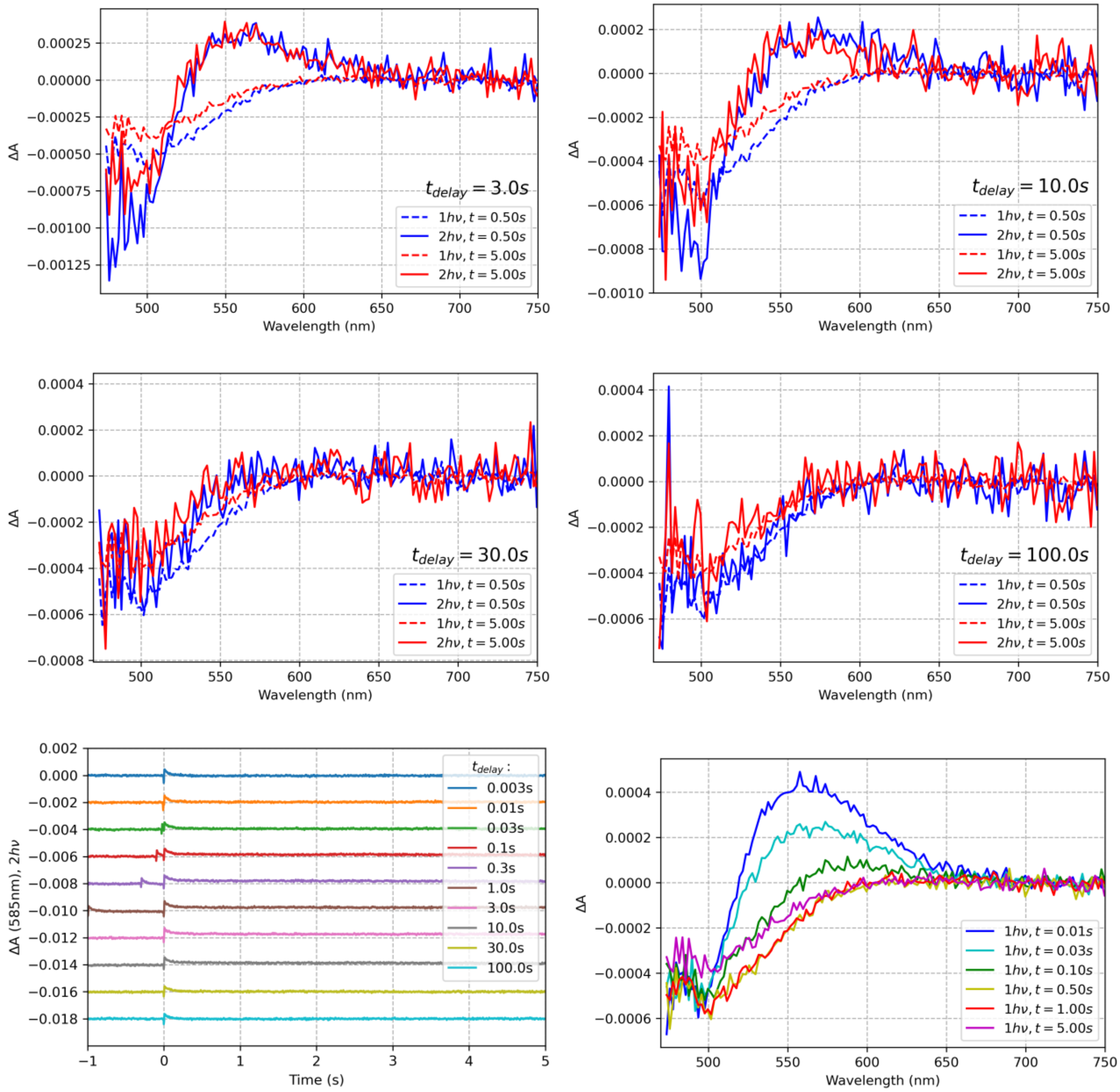

Figure S23. Two pulse experiment results obtained for the OCP L37V mutant from *Synechocystis* functionalized with ECN, with His-tag removed. Excitation at 512 nm, energy density about 50 mJ/cm^2.

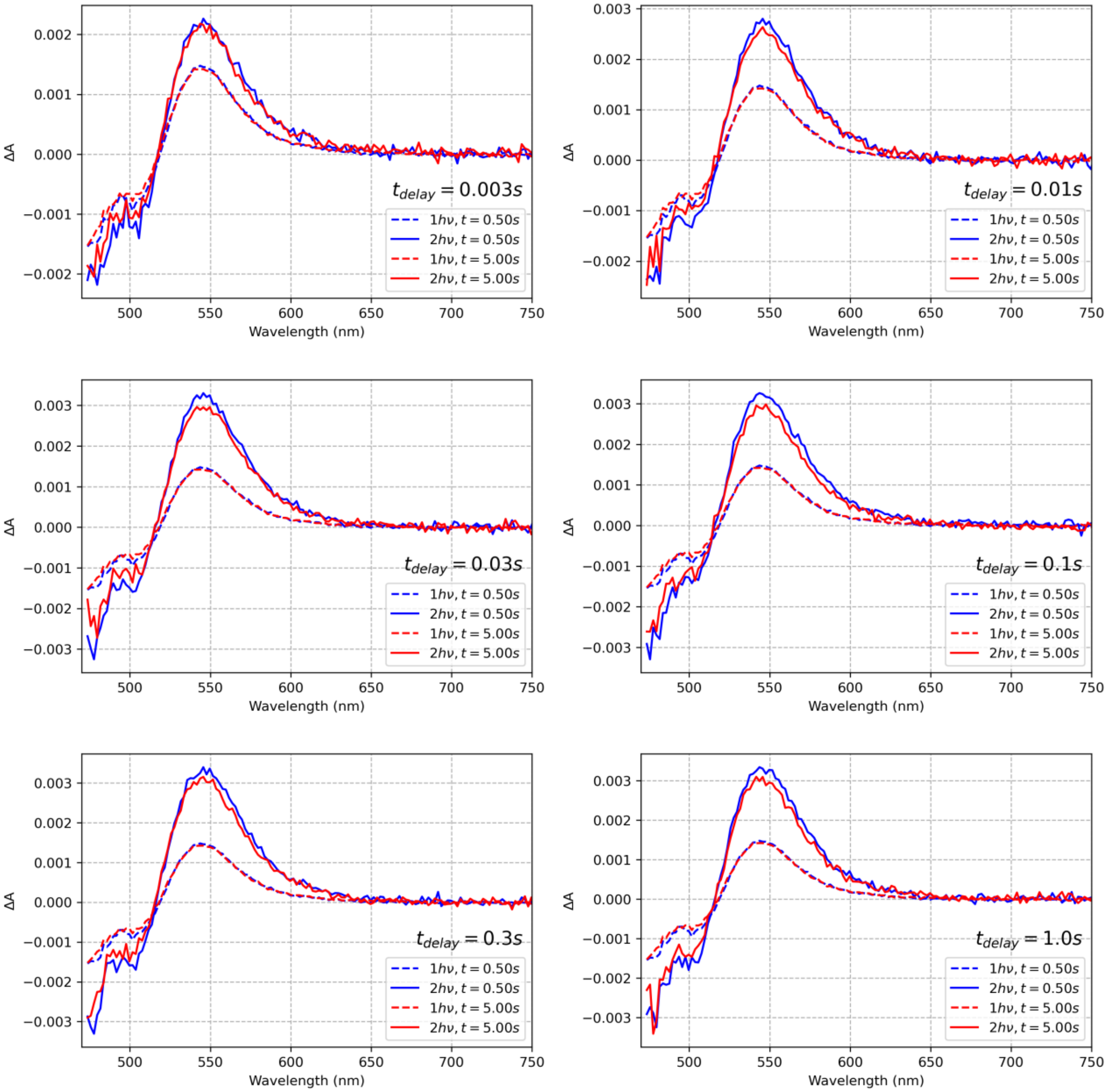
ΔA
Wavelength (nm)
$t_{delay} = 0.003s$
$t_{delay} = 0.01s$
$t_{delay} = 0.03s$
$t_{delay} = 0.1s$
$t_{delay} = 0.3s$
$t_{delay} = 1.0s$
$1h\nu, t = 0.50s$
$2h\nu, t = 0.50s$
$1h\nu, t = 5.00s$
$2h\nu, t = 5.00s$

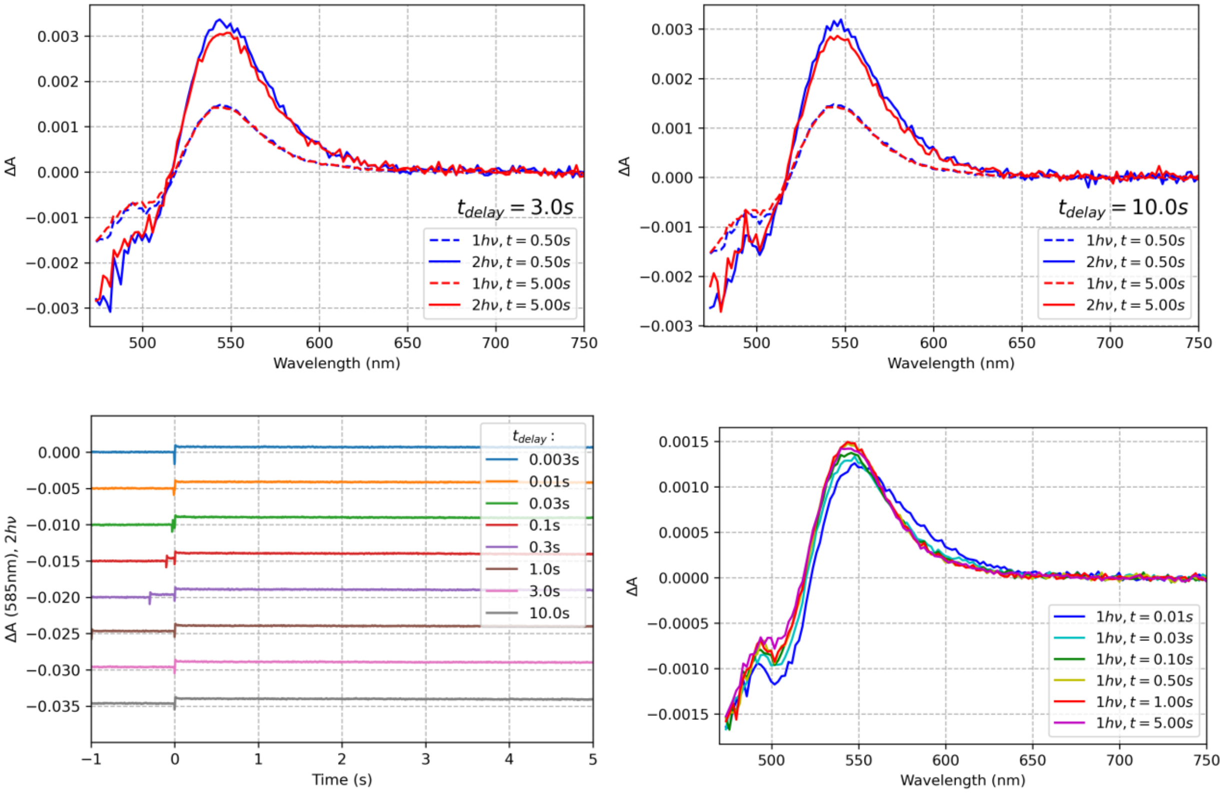

Figure S24. Two pulse experiment results obtained for the OCP mutant L37V from *Synechocystis* functionalized with CAN, with His-tag removed. Excitation at 512 nm, energy density about 50 mJ/cm^2.

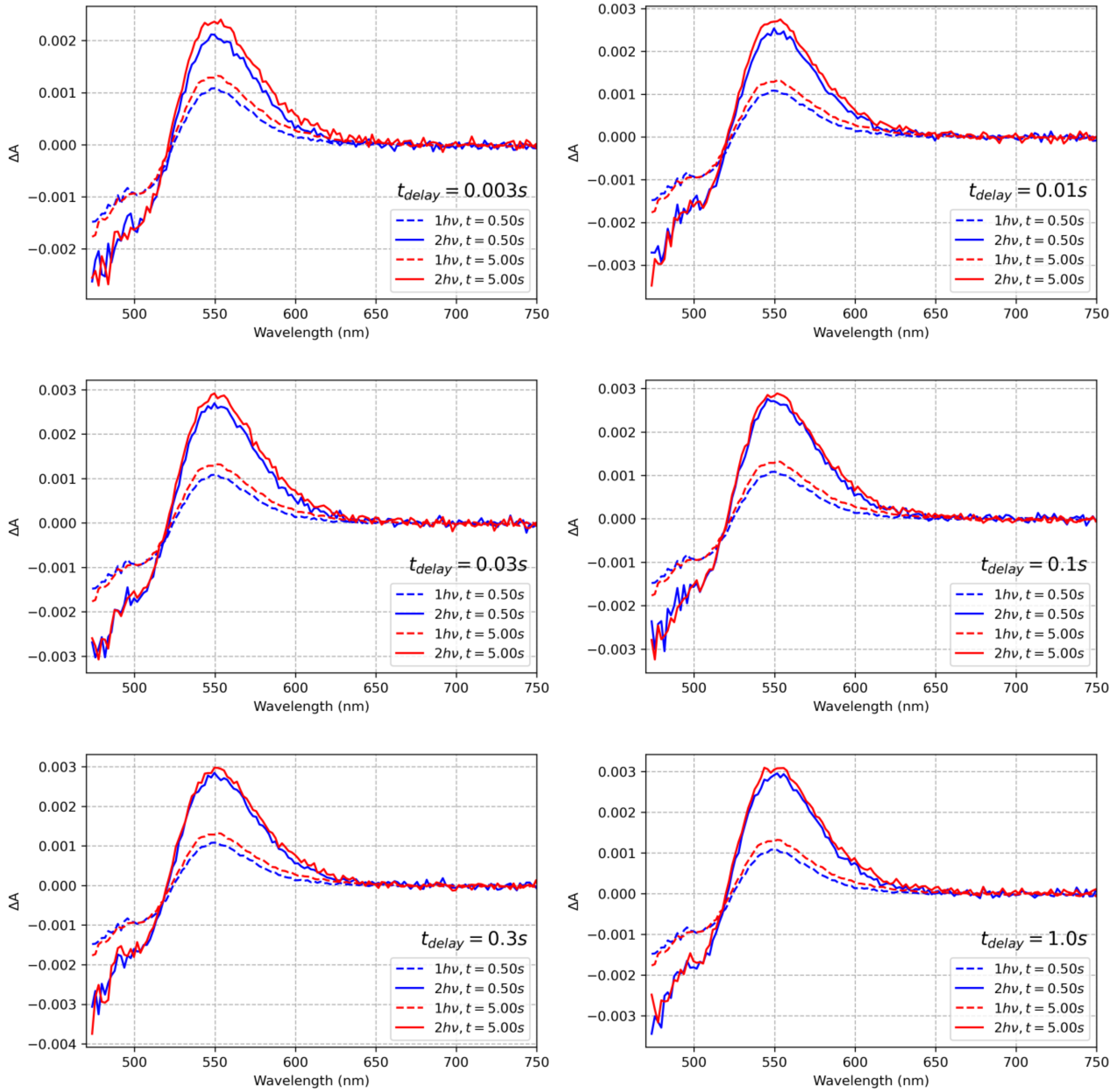

$t_{delay} = 0.003s$
$t_{delay} = 0.01s$
$t_{delay} = 0.03s$
$t_{delay} = 0.1s$
$t_{delay} = 0.3s$
$t_{delay} = 1.0s$
$1h\nu, t = 0.50s$
$2h\nu, t = 0.50s$
$1h\nu, t = 5.00s$
$2h\nu, t = 5.00s$
ΔA
Wavelength (nm)

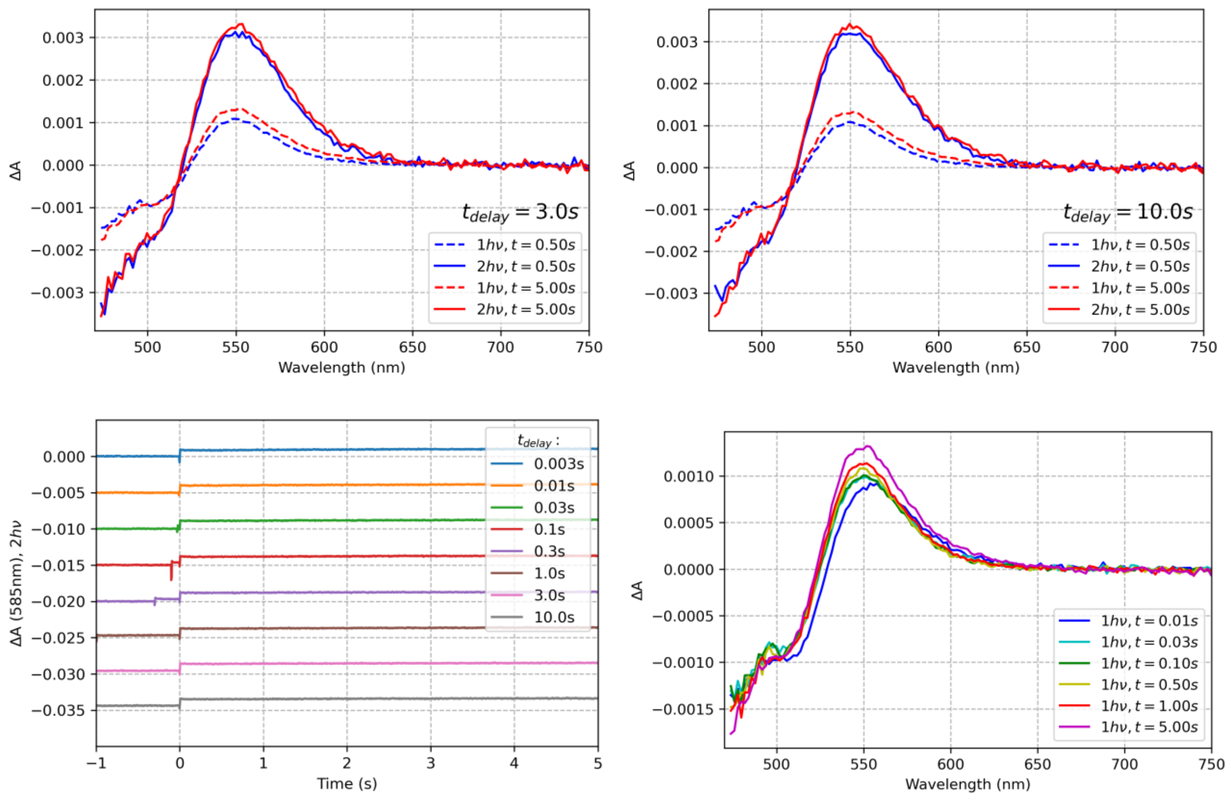

Figure S25. Two pulse experiment results obtained for the OCP mutant L37A from *Synechocystis* functionalized with CAN, with His-tag removed. Excitation at 512 nm, energy density about 50 mJ/cm^2.

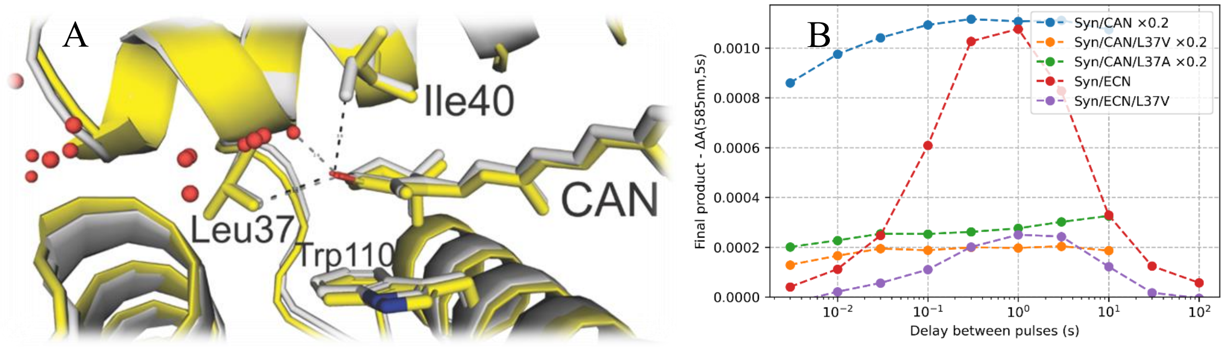

Figure S26. Environment of the carotenoid β2 ionone ring. A) Overlay of structures of CAN complexed OCP. In the *Synechocystis* variant (PDB 7ZSF, yellow, similar for PDB 4XB5)) the methyl group of Ile40 points away from the CAN keto-group, making room for a water molecule (which was not modeled in the structure, distance 2.6 Å). In the *Planktothrix* variant (PDB 7QD2, grey), the methyl group of Ile40 points towards the CAN keto-group (distance 3.6 Å), preventing binding of a water molecule. The distance between the keto-group and the methyl group of Leu37 is ~3.5 Å in OCP_{Syn} and 3.2 Å in OCP_{Plk}. Water molecules are shown as red spheres. B) The change in absorption at 585 nm (5 s after the second pulse), representing the yield of OCP^R, plotted against t_{delay}. Excitation at 512 nm, energy density about 50 mJ/cm^2.

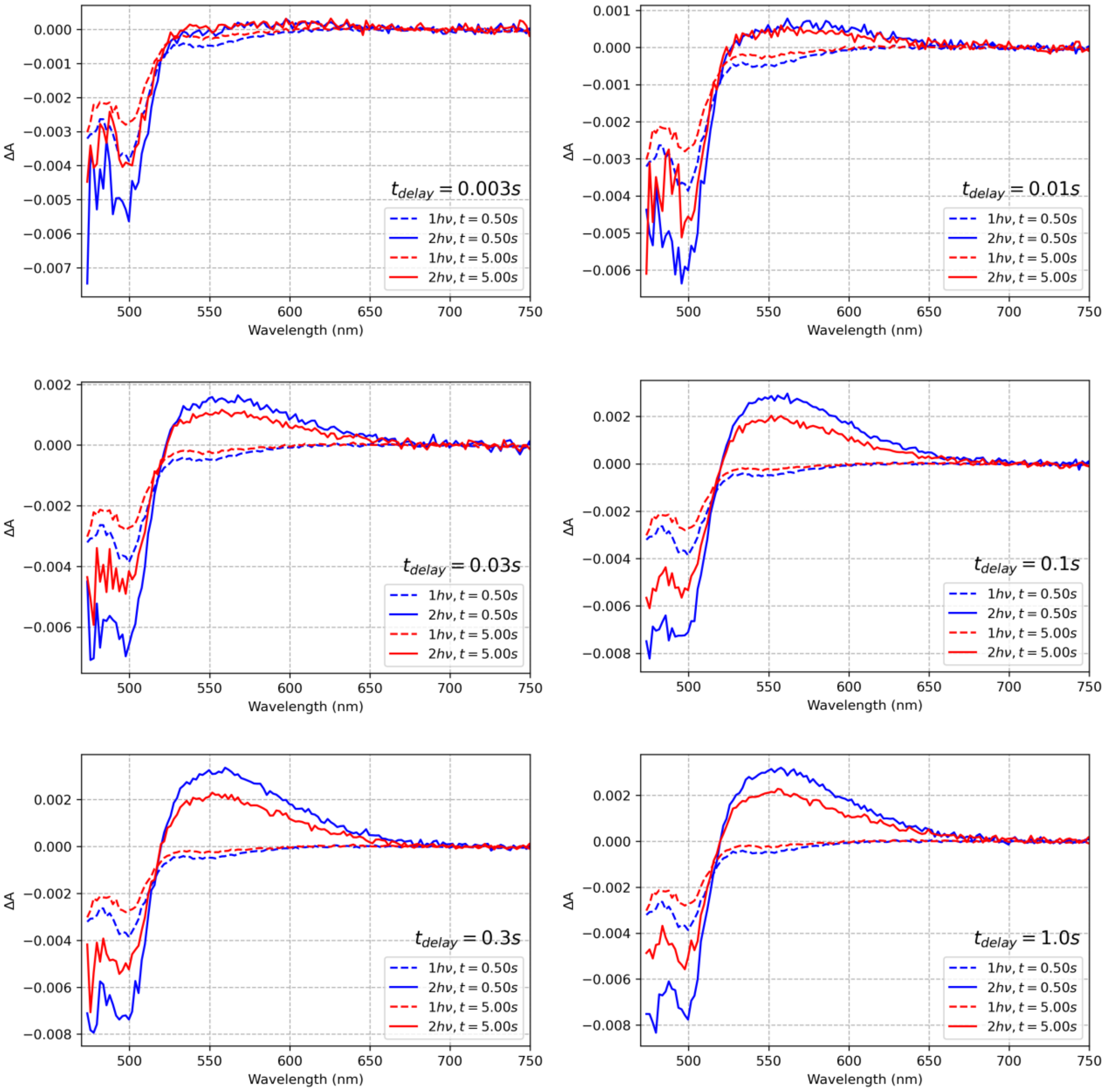
ΔA
Wavelength (nm)
$t_{delay} = 0.003s$
$t_{delay} = 0.01s$
$t_{delay} = 0.03s$
$t_{delay} = 0.1s$
$t_{delay} = 0.3s$
$t_{delay} = 1.0s$
$1h\nu, t = 0.50s$
$2h\nu, t = 0.50s$
$1h\nu, t = 5.00s$
$2h\nu, t = 5.00s$

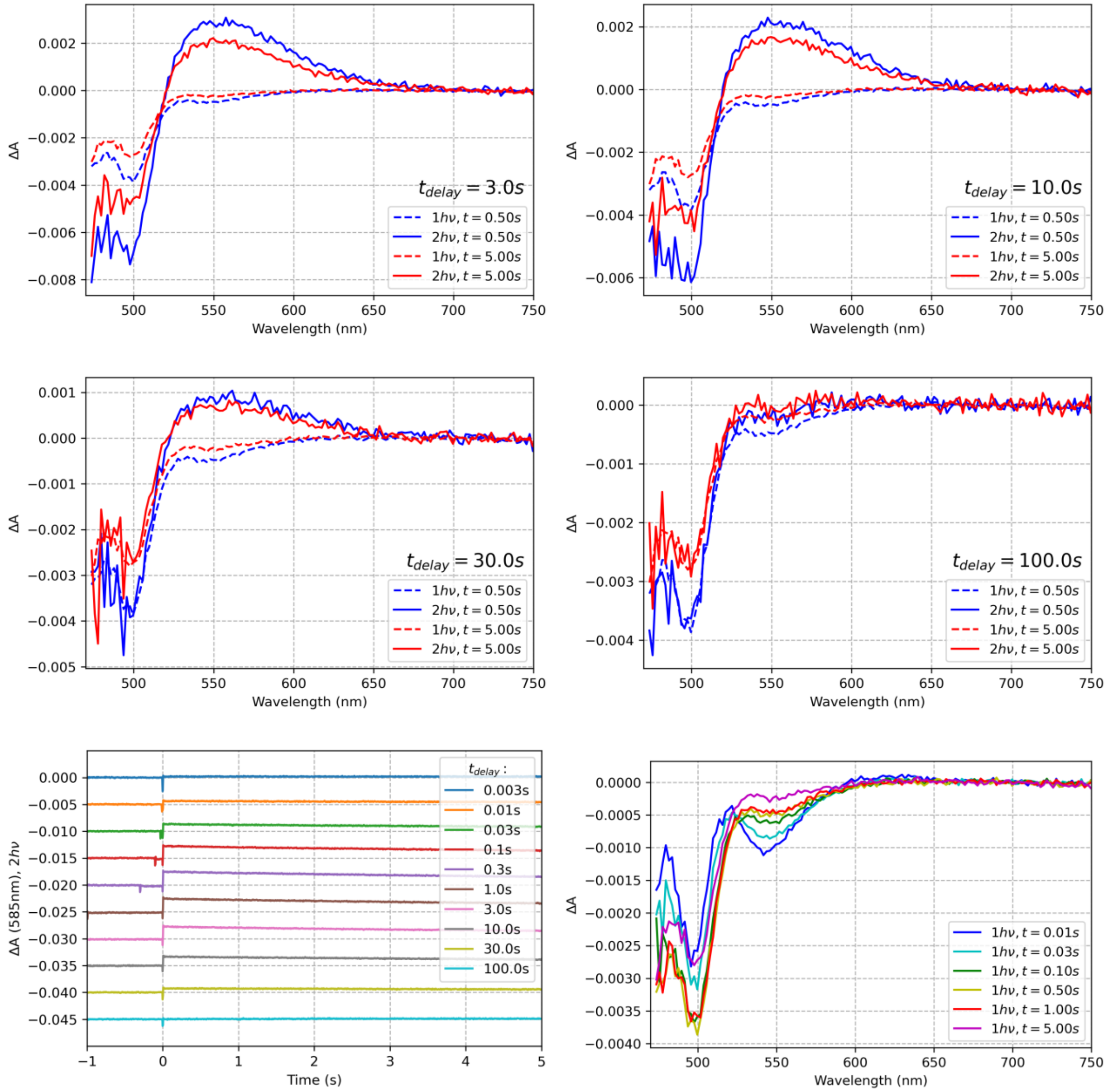

Figure S27. Two pulse experiment results obtained for the OCP mutant R27L from *Planktothrix* functionalized with ECN, with His-tag removed. Excitation at 512 nm, energy density about 50 mJ/cm^2.

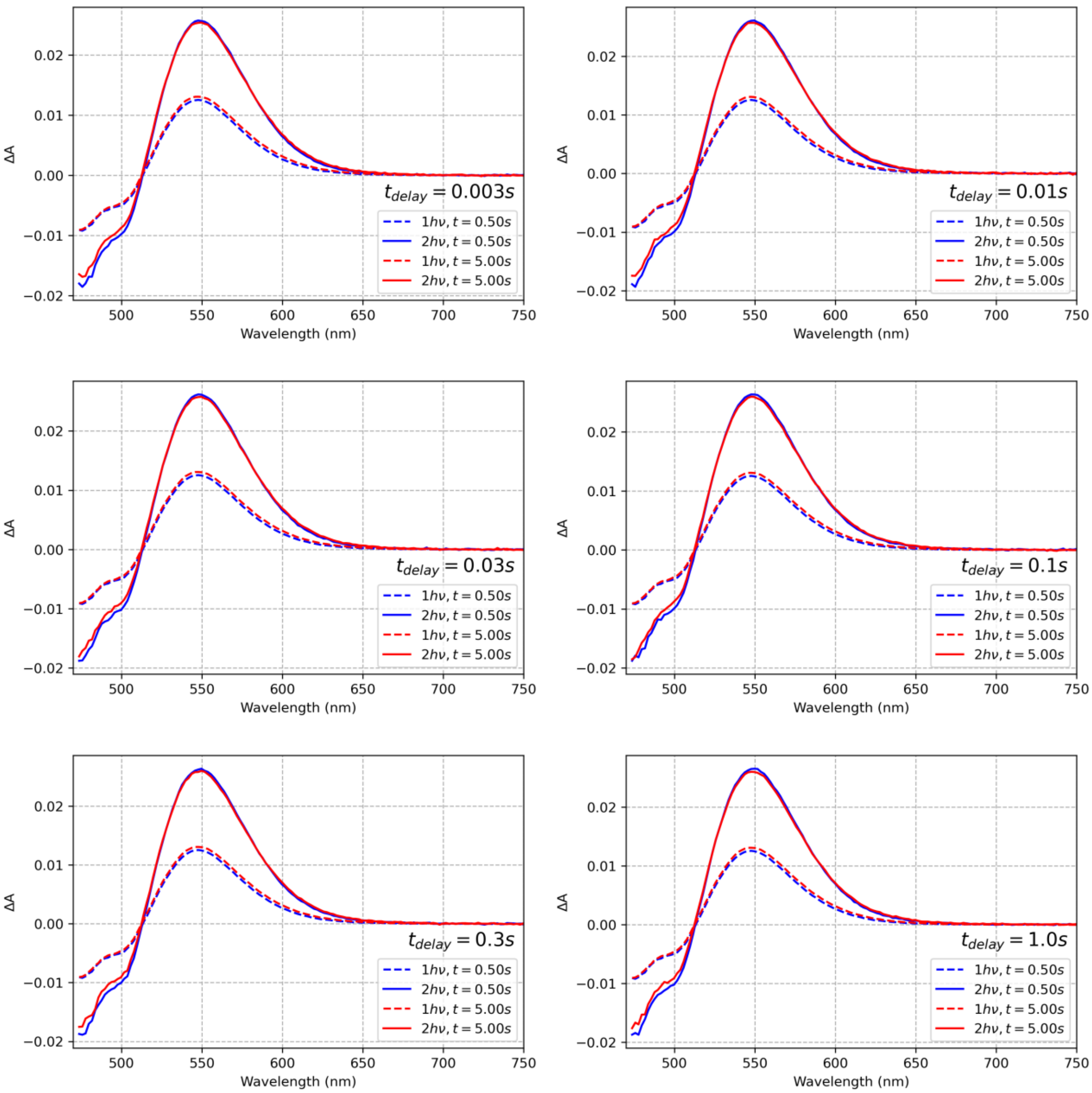
ΔA
Wavelength (nm)
$t_{delay} = 0.003s$
$t_{delay} = 0.01s$
$t_{delay} = 0.03s$
$t_{delay} = 0.1s$
$t_{delay} = 0.3s$
$t_{delay} = 1.0s$
$1h\nu, t = 0.50s$
$2h\nu, t = 0.50s$
$1h\nu, t = 5.00s$
$2h\nu, t = 5.00s$

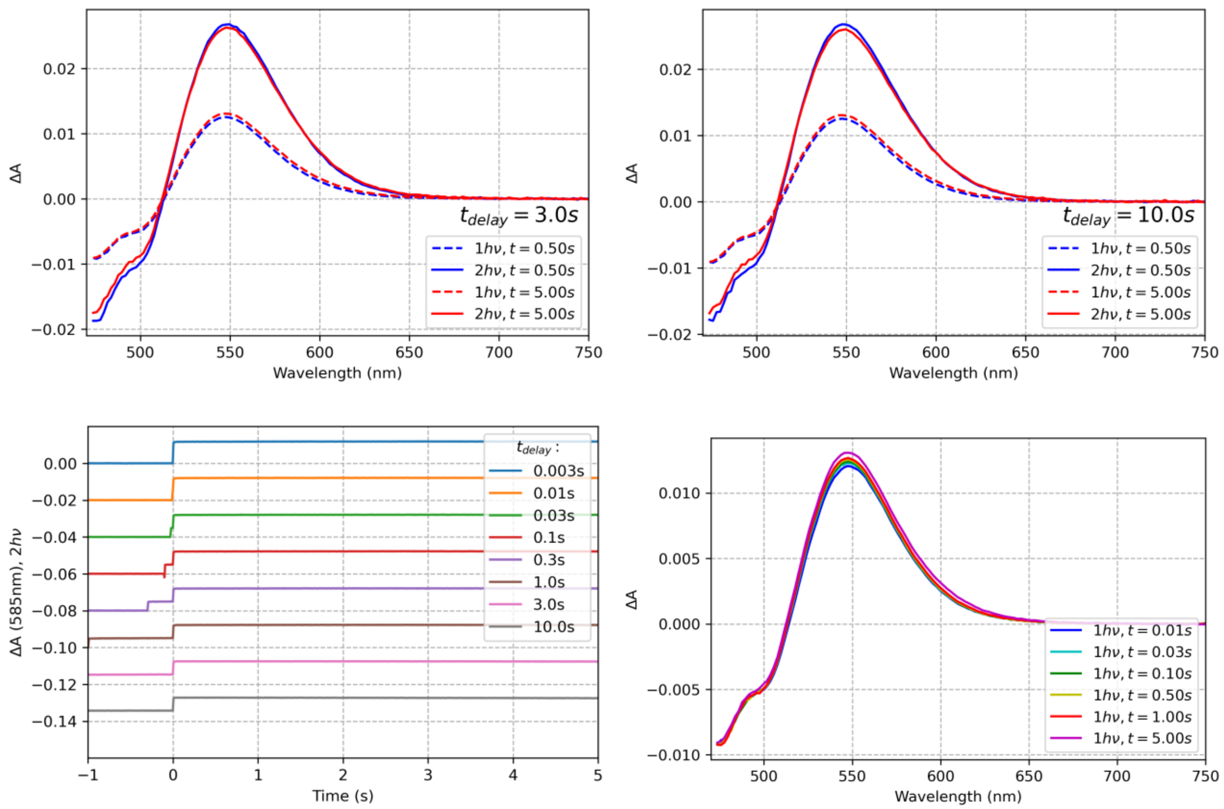

Figure S28. Two pulse experiment results obtained for the OCP mutant R27L from *Planktothrix* functionalized with CAN, with His-tag removed. Excitation at 512 nm, energy density about 50 mJ/cm^2.

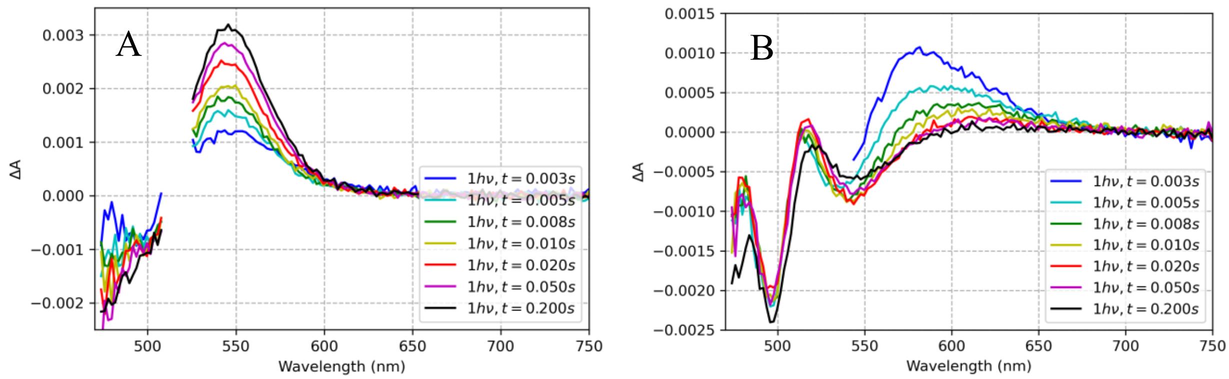

Figure S29. Spectra obtained for shortest delays available in our setup for A) OCP from *Synechocystis* functionalized with CAN, with His-tag removed, B) OCP from *Synechocystis* functionalized with ECN, with His-tag removed.

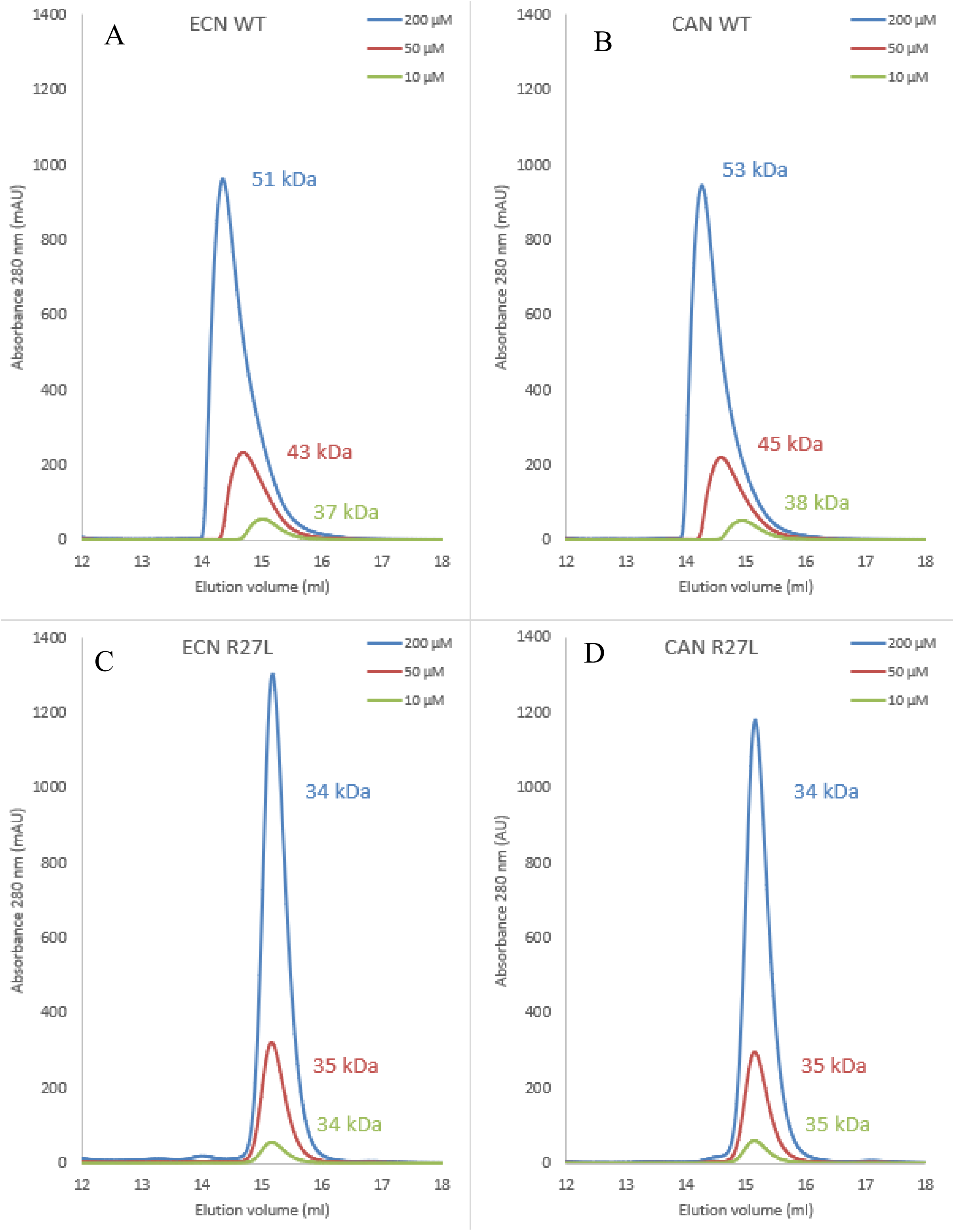

Figure S30. Size exclusion chromatography chromatograms done with 280 nm probe. A) WT OCP functionalized with ECN and with B) CAN. C) R27L OCP mutant functionalized with ECN and with D) CAN. All samples are from *Planktothrix*.

3. Decomposition of the experimental datasets

In order to better understand data obtained after the single excitation laser pulse, the decomposition was performed using the TIMP package implemented in R.[11] A three exponential sequential decaying model was used, with a third component set to infinity. This represents the scheme $\mathrm{OCP^O} \xrightarrow{h\nu} \mathrm{OCP^X} \xrightarrow{k_X} \mathrm{OCP^{1h\nu}} \xrightarrow{k_{return}} \mathrm{OCP^O}$. The time constants visible in Figure S31 are $\tau_1 = 1/k_X$ and $\tau_2 = 1/k_{return}$. Note the experimental time window used in these experiments ends 17.5 s after the laser pulse, when the evolution is not fully completed.

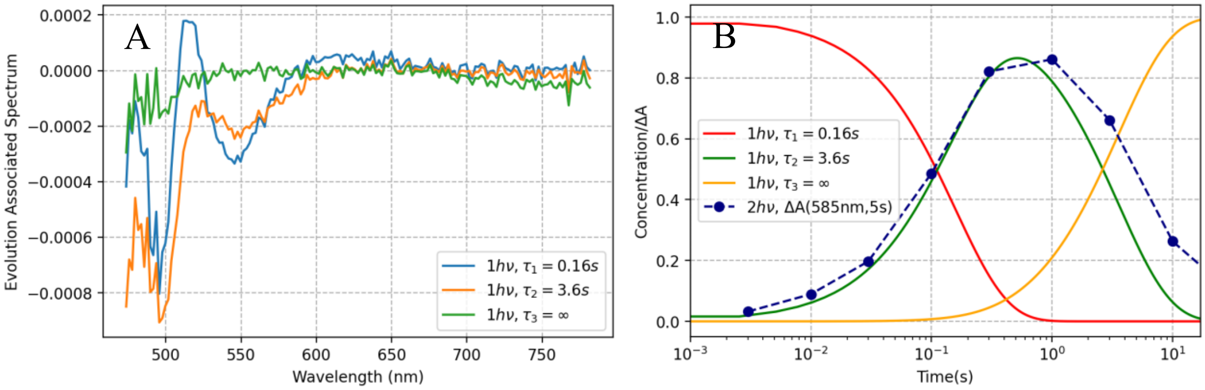

Figure S31. A) Evolution Associated Spectra (EAS), obtained for OCP from *Synechocystis* functionalized with ECN, with His-tag removed, after one laser pulse of energy density of about 3 mJ/cm^2. B) Concentration profiles superimposed with a rescaled curve representing the $\mathrm{OCP^R}$ population after two laser pulses (ΔA signal at 585 nm, 5 s after the first laser pulse) plotted as a function of t_{delay}.

4. Supplementary References

1. Wilson, A.; Andreeva, E. A.; Nizinski, S. J.; Talbot, L.; Hartmann, E.; Schlichting, I.; Burdzinski, G.; Sliwa, M.; Kirilovsky, D.; Colletier, J. P., Structure-function-dynamics relationships in the peculiar *Planktothrix* PCC7805 OCP1: Impact of his-tagging and carotenoid type. *Biochim Biophys Acta Bioenerg* **2022,** *1863* (7), 148584.
2. Niziński, S.; Schlichting, I.; Colletier, J. P.; Kirilovsky, D.; Burdziński, G.; Sliwa, M., Is Orange Carotenoid Protein photoactivation a single-photon process? *Biophys. Rep.* **2022,** *2* (3), 100072.
3. Chalk, S. J. IUPAC. Compendium of Chemical Terminology, 2nd ed. (the "Gold Book"). https://goldbook.iupac.org/terms/view/Q04991.
4. Maksimov, E. G.; Protasova, E. A.; Tsoraev, G. V.; Yaroshevich, I. A.; Maydykovskiy, A. I.; Shirshin, E. A.; Gostev, T. S.; Jelzow, A.; Moldenhauer, M.; Slonimskiy, Y. B.; Sluchanko, N. N.; Friedrich, T., Probing of carotenoid-tryptophan hydrogen bonding dynamics in the single-tryptophan photoactive Orange Carotenoid Protein. *Sci. Rep.* **2020,** *10* (11729), 1-12.
5. ASTM G-173. https://www.nrel.gov/grid/solar-resource/spectra-am1.5.html (accessed 2024-09-20).
6. Kish, E.; Pinto, M. M.; Kirilovsky, D.; Spezia, R.; Robert, B., Echinenone vibrational properties: From solvents to the Orange Carotenoid Protein. *Biochim Biophys Acta* **2015,** *1847* (10), 1044-1054.
7. Šlouf, V.; Kuznetsova, V.; Fuciman, M.; Bourcier de Carbon, C.; Wilson, A.; Kirilovsky, D.; Polívka, T., Ultrafast spectroscopy tracks carotenoid configurations in the orange and red carotenoid proteins from cyanobacteria. *Photosynth. Res.* **2017,** *131* (1), 105–117.
8. Muzzopappa, F.; Wilson, A.; Kirilovsky, D., Interdomain interactions reveal the molecular evolution of the orange carotenoid protein. *Nat. Plants* **2019,** *5*, 1076–1086.
9. Andreeva, E. A.; Nizinski, S.; Wilson, A.; Levantino, M.; De Zitter, E.; Munro, R.; Muzzopappa, F.; Thureau, A.; Zala, N.; Burdzinski, G.; Sliwa, M.; Kirilovsky, D.; Schirò, G.; Colletier, J. P., Oligomerization processes limit photoactivation and recovery of the Orange Carotenoid Protein. *Biophys J.* **2022,** *121* (15), 2849-2872.
10. Zhang, H.; Liu, H.; Niedzwiedzki, D. M.; Prado, M.; Jiang, J.; Gross, M. L.; Blankenship, R. E., Molecular mechanism of photoactivation and structural location of the cyanobacterial Orange Carotenoid Protein. *Biochemistry* **2014,** *53* (1), 13–19.
11. Mullen, K. M.; van Stokkum, I. H. M., TIMP: An R package for modeling multi-way spectroscopic measurements. *J. Stat. Softw.* **2007,** *18* (3), 1-46.
